# Spatial Analysis of Malaria in Bangladesh: Insights from Bayesian Disease Mapping Models

**DOI:** 10.1371/journal.pone.0353483

**Published:** 2026-08-24

**Authors:** Ummehani Mimi, Md. Rezaul Karim, Sultana Begum, Khadiza Akter Eme

**Affiliations:** 1 Department of Statistics and Data Science, Jahangirnagar University, Savar, Dhaka, Bangladesh; 2 Department of Physical Sciences, Independent University, Dhaka, Bangladesh; Bahir Dar University, ETHIOPIA

## Abstract

**Background and aims:**

Malaria remains a public health concern in Bangladesh, despite a notable decline in reported cases since 2012 and a brief resurgence in 2014. Aligned with the United Nations Sustainable Development Goal (SDG) 3.3, which targets the elimination of malaria by 2030, Bangladesh has implemented multiple control and prevention strategies. This study assesses the significance of the decline in malaria cases and applies Bayesian hierarchical models to capture spatial dynamics of malaria-related vulnerability, map district-level risk, and inform targeted interventions.

**Methods:**

A nationwide spatial analysis was conducted using district-level malaria data from the Bangladesh Disaster-related Statistics (BDRS) 2021, which report cumulative counts of “population suffering from malaria due to disaster” for 2015–2020. These data were used as a proxy indicator of relative malaria vulnerability. National malaria time-series data were obtained from Bangladesh’s National Strategic Plan for Malaria Elimination (2021–2025) published by the Asia Pacific Malaria Elimination Network (APMEN), together with malaria surveillance summaries from the World Malaria Reports (2024,2025) published by the World Health Organization (WHO). District-level rainfall data were obtained from the Bangladesh Water Development Board. Bayesian hierarchical disease mapping models were used to assess spatial dependence and district-level malaria risk. Spatial visualization and autocorrelation analyses (Global Moran’s I, Geary’s C, and Local Moran’s I) were performed using R (version 4.4.0), and Bayesian model estimation was carried out using WinBUGS via Markov Chain Monte Carlo methods.

**Results:**

The analysis showed evidence of a trend in decreasing malaria cases by a value of −0.691 in the Mann-Kendall trend test. However, spatial analysis revealed significant clustering and geographic heterogeneity. Persistent high-risk clusters were identified in the southeastern hilly regions. Additionally the presence of excess zeros in the data justified the use of zero-inflated models.

**Conclusion:**

The findings show significant national decline and offer valuable insights into the geographical variability in malaria-related vulnerability in Bangladesh. Rather than being direct indicators of malaria transmission, the results should be understood as representing relative risk patterns based on data related to disasters. Under current data limitations, these results provide evidence to boost spatially informed malaria control methods and assist geographically focused interventions.

## 1. Introduction

Malaria has been an age-old disease in Bangladesh [1]. Malaria was nearly eliminated in the country by the 1970s; however, it has never completely disappeared [2]. In the 1990s, malaria re-emerged and the Government of Bangladesh (GoB) declared malaria as a public health problem due to high morbidity and mortality and continues to present an important challenge till 2012 indicating to take necessary step and action [2]. However there has been a decrease in malaria cases in Bangladesh from 2012 to now with an exception in 2014. After successful interventions, malaria's Years of Life Lost (YLLs) burden decreased from rank 18th in1990–44th in 2010 [1]. Being a United Nation (UN) member, along with other Sustainable Development Goals (SDGs) Bangladesh is committed to fulfill target 3.3: “End the epidemics of AIDS, tuberculosis, malaria, and neglected tropical diseases (NTDs), and combat hepatitis, water-borne diseases, and other communicable diseases by 2030” under SDG-3: “Good Health and Well-Being.” The National Malaria Elimination Programme (NMEP) launched by the GoB focusses on malaria prevention, diagnosis, treatment, surveillance, and monitoring in an effort to eradicate the disease. The goal of NMEP's 2024–2030 plan is to achieve and maintain zero malaria-related deaths by 2027 while eradicating malaria and preventing its resurgence by 2030 [1]. The objectives of the plan place a high priority on universal access to timely diagnosis, efficient treatment, and focused prevention through 2030 [1]. Additionally, it highlights enhanced surveillance, preparedness for outbreaks, community involvement, and communication about social and behavioural change [1]. Strong program management, collaborations, research, and prevention of malaria re-establishment in malaria-free areas are further highlighted in the strategy [1].

Female Anopheles mosquitoes carrying parasites that cause malaria, which they can transmit to humans through their bites, leading to illness [3]. *Plasmodium vivax*, *P. Falciparum*, *P. Ovale, P. Malariae* and *P.knowlesi* are the five parasite species that cause malaria [4]. A patient is considered malaria positive when any of the plasmodium parasites are detected in the blood. Anopheles mosquitoes typically do not fly more than 1.2 miles (2 km) from their larval habitats, resulting in nearby areas potentially having similar malaria patterns [3]. Additionally, the reproduction of mosquitoes is significantly influenced by environmental factors, and adjacent areas tend to have similar meteorological conditions. So, from the perspective of spatial epidemiology and disease ecology, malaria transmission is inherently spatial. As these mechanisms lead to spatial dependency and risk clustering, spatial statistical approaches are essential for accurate disease mapping and inference.

Bayesian hierarchical disease mapping models are particularly useful for evaluating malaria data because they explicitly take spatial dependency and uncertainty in risk assessment into account [5]. By combining observed data with previous(prior) information, Bayesian methods—in contrast to frequentist methods—allow for steady estimate in areas with low case counts and more accurate identification of high-risk areas. These techniques have been widely applied in spatial epidemiology to support evidence-based public health decision making.

Epidemiological studies on malaria using spatial statistics have been used in different geographical regions like India [6], Nepal [7], and sub-Saharan Africa [8], consistently demonstrating spatial clustering, ecological heterogeneity, and persistent hotspots, thereby supporting the use of spatial statistical modeling for malaria risk assessment.

Prior spatial analyses of malaria in Bangladesh have mostly concentrated on particular high-endemic districts or border areas and some studies have further restricted their scope to infections caused by *P. Falciparum* infections [9–13]. While prioritizing high-endemic districts is operationally important, the absence of a simultaneous nationwide spatial assessment limits the ability to obtain a comprehensive understanding of malaria risk across Bangladesh. Consequently, recent nationwide spatial analyses covering all malaria species-that is- all reported malaria cases and using updated data remain scarce in Bangladesh, representing a critical gap in the literature. To overcome the gap, it is essential to conduct a nationwide analysis using the recent data.

Against this background, this study aims to provide a nationwide spatial assessment of malaria burden in Bangladesh using the only publicly available district-level data source. Specifically, the objectives are:

- examine whether the decline in malaria cases in Bangladesh is statistically significant;
- assess the presence of spatial dependence and clustering in district-level malaria-related cases as captured by the available disaster-related malaria statistics; and
- identify relative high-risk and low-risk districts using Bayesian hierarchical disease mapping models.

This comprehensive national-level spatial analysis directly supports the SDG target 3.3 by pinpointing districts with higher or lower vulnerability. The study enables evidence-based prioritization of interventions and assesses whether the NMEP geographically targeted strategies align with malaria burden patterns. Ultimately, these findings provide actionable insights to strengthen targeted malaria elimination efforts in settings with available limited data.

## 2. Materials and methods

### 2.1 Data sources

**Fig 1:**
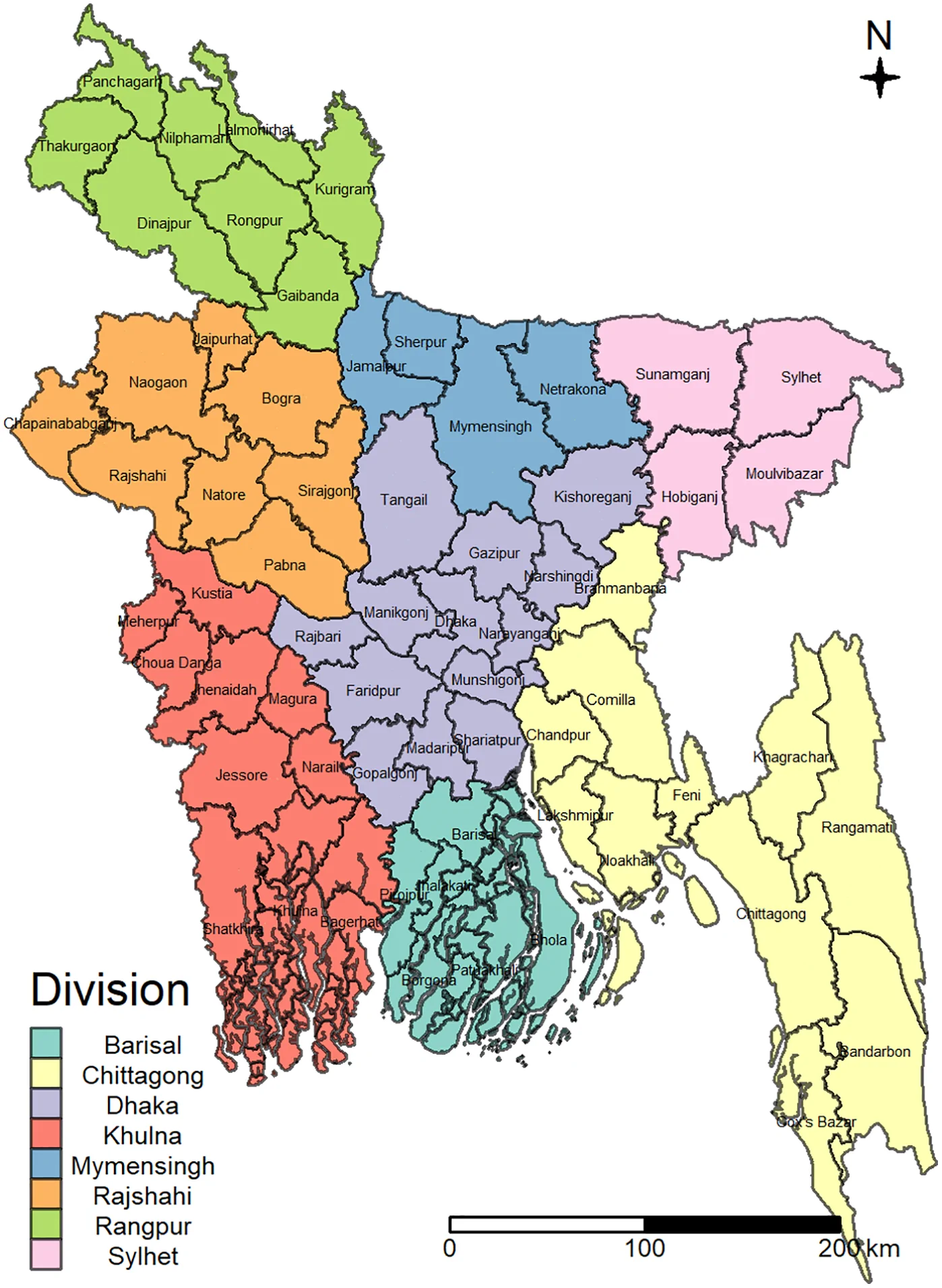
Study area of this study (map of Bangladesh by divisions and districts). Administrative boundary data were obtained from the GADM (www.gadm.org), which permits the use of derived maps in academic publications under open licenses. Maps were generated by the authors using R. The figure is an original visualization and contains no proprietary basemap layers; it is compliant with CC BY 4.0 licensing.

The area of this study is Bangladesh which is shown in Fig 1. The malaria affected case counts over the years are obtained from the Bangladesh’s National Strategic Plan for Malaria Elimination (2021–2025) published by the Asia Pacific Malaria Elimination Network (APMEN), which is justified from the World Malaria Reports (2024,2025) published by WHO.

District-level malaria surveillance data from the NMEP are not publicly accessible in Bangladesh. These data are managed through the DHIS2 platform, which is restricted to authorized personnel. Consequently, nationwide district-level malaria data are not readily available for independent research. For this study, we therefore rely on district-level counts of “malaria due to disaster” reported in the Bangladesh Disaster-related Statistics (BDRS) 2021, published by the Bangladesh Bureau of Statistics (BBS). To date, this dataset represents the only publicly available source providing district-wise malaria-related information covering the entire country.

In the BDRS 2021 report published by the BBS, malaria cases are reported under the category “distribution of population suffering from malaria due to disaster” as part of district-level assessments of disaster-related health impacts among affected households between 2015 and 2020. Importantly, the reported values represent a single cumulative (aggregated) statistic for the entire period 2015–2020. Therefore, each district is represented by one value reflecting the total burden over the period. This category reflects the increased health risks linked to environmental disruption brought on by floods, cyclones, waterlogging, salinity intrusion, and other climate-related hazards. In this study, “distribution of population suffering from malaria due to disaster” is treated as a proxy indicator of relative malaria burden and vulnerability at the district level. As it captures malaria-related illness reported by households due to disaster events, when transmission risk is known to increase. Disasters such as floods and cyclones worsen malaria risk by creating mosquito breeding sites, displacing populations, and disrupting access to healthcare. As a result, districts that repeatedly report malaria in disaster contexts are likely to be areas where underlying malaria transmission is already present and intensified during periods of environmental stress. As a result, these data may underestimate absolute malaria incidence and exhibit spatial bias toward disaster-prone districts; however, they remain appropriate for identifying relative malaria risk, comparing districts, and analyzing spatial patterns of malaria vulnerability, even though they do not measure routine incidence. The district wise malaria data (due to disaster) were extracted from the Bangladesh Disaster-related statistics 2021 report from BBS [14].

The average rainfall data is obtained from Bangladesh Water Development Board [15,16] which is also available in online.

The population of each district is sourced from the city population website [17], which is publicly available. Bangladesh’s population growth has remained relatively stable in recent years. Specifically, the annual growth rate was approximately 0.9% for 2015–2017 [18], remained around 0.8% in 2018–2019 [18], and was about 1% in 2020 [18]. This demonstrates that growth rates have been nearly constant over the short period. The study projects the population for 2020 using the 2022 census data, making a geometric growth assumption which is reasonable for projecting the population over this short interval. We used the following equation to project the population for the year 2020


$$\begin{array}{c} {\text{P}}_{\text{2020}}={\text{P}}_{\text{2022}}{\left(\text{1}+\text{r}\right)}^{-\text{2}} \end{array}$$ (1)

**Table:** 

r	The geometric growth rate.
P_2020_	The population of Bangladesh at 2020.
P_2022_	The population of Bangladesh at 2022 census.

The geometric growth rate is determined using the formula


$$\begin{array}{c} \text{r}=\sqrt[{\text{ n}}]{\frac{{\text{P}}_{\text{n}}}{{\text{P}}_{\text{0}}}}-\text{1} \end{array}$$ (2)

**Table:** 

r	The geometric growth rate.
n	The time interval between two successive censuses.
P_n_	The population of Bangladesh at 2022 census.
P_0_	The population of Bangladesh at 2011 census.

Using the projected population and malaria data, we estimated the malaria prevalence rate in each district. The formula to determine the prevalence rate is


$$\begin{array}{c} {\text{Prevalence rate}}_{\text{i}}=\;\frac{{\text{Number of people suffering from Malaria in district}}_{\text{i}}\;}{{\text{i}}^{\text{th }}\text{district population}},\text{ i}=\text{1},\text{2},\ldots ,\text{64} \end{array}$$ (3)

Then, an estimated number of affected individuals is calculated per 100,000 in each district.

For the purposes of modeling, it is necessary to have the expected number of malaria cases in each district. As this data is unavailable, we estimate the expected number of malaria cases for each district using


$$\begin{array}{c} {\text{E}}_{\text{i}}={\text{P}}_{\text{i}}\times \frac{\text{Y}}{\text{P}},\text{i}=\text{1},\text{2},\ldots ,\text{64} \end{array}$$ (4)

${\text{E}}_{\text{i}}\;$ is the expected malaria case in district i.

${\text{P}}_{\text{i}}$ is the population of district i.

$\text{P }(=\sum_{\text{i}=\text{1}}^{\text{64}}{\text{P}}_{\text{i}})$ is the total population.

$\text{Y }(=\sum_{\text{i}=\text{1}}^{\text{64}}{\text{Y}}_{\text{i}})$ is the total malaria case.

### 2.2 Distribution of response variable

A Poisson distribution is a discrete probability distribution that represents the likelihood of a countable outcome [19]. Specifically, it is used to model the number of times an event occurs within a fixed interval of time or space [20].

In this study the main variable is the number of malaria cases recorded at various time points and across different districts. Since this variable is discrete, using a Poisson probability distribution is appropriate for representing the malaria case data. The probability mass function of Poisson distribution is [19].


$$\begin{array}{c} \text{Pr }(\text{Y}=\text{y})=\;\frac{{\text{e}}^{-\lambda }\lambda^{\text{y}}}{\text{y}!};\;y\;=\;\text{0},\;\text{1},\;\text{2},\;\text{3},\;\ldots \end{array}$$ (5)

Here, e determines the Euler's number (e = 2.71828…), λ determine the expected number of malaria cases and y determine the observed number of malaria cases respectively. As we are suspecting malaria cases are declining, we may have a high number of districts with zero malaria. Therefore, we can also consider the Zero-Inflated Poisson (ZIP) likelihood for the posterior estimation as well [21]. Zero Inflated data refer to the count data that have more zeros than would be predicted by negative binomial distribution or Poisson distribution, i.e., that include a significant number of zero counts. So, if there is an excess number of zero case counts, Zero-Inflated Poisson (ZIP) is used. In order to anticipate excess zeros, the ZIP model consists of two components: a Poisson count model and a logit model [22]. The distribution is described as


$$\begin{array}{c} \text{Pr}(\text{Y}=\text{0})=\pi +{(\text{1}-\pi )\text{e}}^{-\lambda }\text{ and Pr}(\text{Y}={\text{y}}_{\text{i}})\;=(\text{1}-\pi )\;\frac{{\text{e}}^{-\lambda }\lambda^{{\text{y}}_{\text{i}}}}{{\text{y}}_{\text{i}}!};\text{ y}=\text{1},\;\text{2},\;\text{3},\;\ldots \end{array}$$ (6)

### 2.3 Mann-Kendall trend test

The Mann-Kendall Trend Test is a non-parametric method used to statistically detect if there is a monotonic trend in time series data [23]. As a non-parametric test, it makes no assumptions about the underlying distribution of the data. The trend may be either upward or downward [23]. Although the trend may or may not be linear, a monotonic upward (downward) trend indicates that the variable continuously rises (falls) with time [24]. To determine if malaria cases are significantly decreasing, a Mann-Kendall trend test is appropriate for this assessment. The steps to conduct this test is in the S1 Appendix.

### 2.4 Test of Sen’s slope estimator

Sen’s slope is a non-parametric method used for the performance of checking the statistical linear relationships [25]. It is used to calculate the magnitude of trends in the long-term temporal data [25]. It has the advantage over the linear regression slope that it does not rely on distributional assumption and gross data series errors and outliers do not affect in much [26]. Positive Sen's slope indicates an upward trend and negative Sen’s slope indicates a downward trend [26].

### 2.5 Spatial data and spatial autocorrelation

Data that refers to a particular geographic region or location, either directly or indirectly, is referred to as spatial data [27]. This reference can be mainly done by three types geo-statistical data, areal data and point-referenced data. We are observing malaria cases (due to disaster) in 64 districts of Bangladesh which are defined by their administrative boundaries. This indicates that we are using areal data type among the three types of spatial data.

Let’s delve into the concept of autocorrelation first to understand spatial autocorrelation. Autocorrelation is a powerful measure that evaluates how a variable correlates with itself, whether through time (temporal autocorrelation) or within spatial contexts (spatial autocorrelation) [28]. Spatial autocorrelation is an assessment of the correlation of a variable in reference to spatial location of the variable, i.e., it is the correlation of a variable with itself over space. If the observations ${\text{y}}_{\text{1}},{\text{y}}_{\text{2}},\ldots ,{\text{y}}_{\text{n}}\;$ of a geo-referenced variable Y display interdependence over space, the data are said to be spatially autocorrelated [29]. When spatial autocorrelation exists, the value of an observation is related to the values of the same variable in its neighboring observations thus making violation of independent observations [28]. It is possible to assess the geographic dependency of values for the same variable across different locations using spatial autocorrelation indices [28]. Spatial autocorrelation is positive when similar values of the variable being studied are geographically clustered. Conversely, spatial autocorrelation is negative when dissimilar values are geographically distributed, indicating that local areas differ more than those farther apart [28].

### 2.6 Global Moran’s I

To effectively measure spatial autocorrelation, two prominent indices stand out: Moran’s I and Geary’s C. An adaption of Pearson's correlation coefficient, Moran's I is calculated by comparing each observed area i to its neighboring areas using the weights, ${\text{w}}_{\text{ij}}$, from the proximity matrix for all j = 1,2, …, n [30]. The global Moran’s I takes the form


$$\begin{array}{c} \text{I}=\frac{\text{n}}{\sum_{\text{i≠j}}{\text{w}}_{\text{ij}}\;}\;\frac{\sum_{\text{i}=\text{1}}^{\text{n}}\sum_{\text{j}=\text{1}}^{\text{n}}{\text{w}}_{\text{ij}}({\text{Y}}_{\text{i}}-\overset{―}{\text{Y}})({\text{Y}}_{\text{j}}-\overset{―}{\text{Y}})}{\sum_{\text{i}=\text{1}}^{\text{n}}{\left({\text{Y}}_{\text{i}}-\overset{―}{\text{Y}}\right)}^{\text{2}}} \end{array}$$ (7)

where ${\text{Y}}_{\text{i}}$; i = 1,2, …, n is the random sample from the n areal units and $\overset{―}{\text{Y}}$ is the sample mean and in our case n = 64 [30].

As a correlation measure, Moran’s I lies in the interval [−1, 1] where a negative value indicates negative spatial autocorrelation, a positive value indicates positive correlation, and zero indicates no spatial correlation [30].

### 2.7 Geary’s C statistic

We can also use the Geary C statistic to measure spatial autocorrelation as a substitute for Moran’s I. The formula of Geary C statistic is [30].


$$\begin{array}{c} \text{C}=\frac{\left(\text{n}-\text{1}\right)}{\text{2}\sum_{\text{i≠j}}{\text{w}}_{\text{ij}}}\;\frac{\sum_{\text{i}=\text{1}}^{\text{n}}\sum_{\text{j}=\text{1}}^{\text{n}}{\text{w}}_{\text{ij}}{\left({\text{Y}}_{\text{i}}-{\text{Y}}_{\text{j}}\right)}^{\text{2}}}{\sum_{\text{i}=\text{1}}^{\text{n}}{\left({\text{Y}}_{\text{i}}-\overset{―}{\text{Y}}\right)}^{\text{2}}}\; \end{array}$$ (8)

where ${\text{Y}}_{\text{i}}$; i=1,2,…,n is the random sample from the n areal units and $\overset{―}{\text{Y}}$ is the sample mean and ${\text{w}}_{\text{ij}}$ is the value of the spatial weight of unit i and j.

The primary distinction between Moran's I and Geary's C statistic lies in the range of their values. Moran's I can take any value from −1 to +1, which is typical for correlation measures. In contrast, Geary C's value falls between 0 and 2 [31]. There is positive autocorrelation if the value is between 0 and 1 [31]. Furthermore, it suggests little spatial autocorrelation if it falls under C ≥ 1 [31]. Conversely, it signifies the existence of negative autocorrelation if it falls between 1 and 2 [31].

But it is essential to conduct a hypothesis test for spatial autocorrelation before proceeding with the calculations of these indices. The testing procedure is in the S1 Appendix.

### 2.8 Getis-Ord general G

Getis-Ord General G measures the concentration of high or low values for a given study area [32]. It is used to determine whether high-value or low-value clustering is present [32]. Being a global statistic, a single statistic is calculated for the entire study [33]. The formula to calculate Getis-Ord General G is [33]


$$\begin{array}{c} \text{G}=\frac{\sum_{\text{i}=\text{1}}^{\text{n}}\sum_{\text{j}=\text{1}}^{\text{n}}{\text{w}}_{\text{ij}}{\text{Y}}_{\text{i}}{\text{Y}}_{\text{j}}}{\sum_{\text{i}=\text{1}}^{\text{n}}\sum_{\text{j}=\text{1}}^{\text{n}}{\text{Y}}_{\text{i}}{\text{Y}}_{\text{j}}} \end{array}$$ (9)

Here, ${\text{Y}}_{\text{i}}$, ${\text{Y}}_{\text{j}}$ are the value at unit i and j from the n areal units

and ${\text{w}}_{\text{ij}}$ is the value of the spatial weight of unit i and j.

The Getis-Ord General G statistic evaluates clustering by summing the products of neighboring values to identify concentrations of high or low values, whereas Global Moran I assesses spatial autocorrelation based on the covariance among neighboring observations [34]. A G value larger than its expected value indicates potential clustering of hot spots, whereas a G value smaller than its expected value indicates potential clustering of cold spots [34]. The expected value is calculated using the following formula [33]


$$\begin{array}{c} \text{E}[\text{G}]=\frac{\sum_{\text{i}=\text{1}}^{\text{n}}\sum_{\text{j}=\text{1}}^{\text{n}}{\text{w}}_{\text{ij}}}{\text{n}(\text{n}-\text{1})}\; \end{array}$$ (10)

### 2.9 Local Moran’s I

Analyzing the relationship between an area's value and that of its neighboring regions is crucial for a comprehensive understanding of local dynamics. Local Indicators of Spatial Association (LISA) are particularly useful for addressing these types of tasks [28].

Local Moran’s I is one of the most used LISA. Local Moran's I finds local hotspots and cold spots by taking into account the spatial relationships between each observation and its neighbors [31].

For the ${\text{i}}^{\text{th}}$ region Local Moran I is calculated as [35]


$$\begin{array}{c} {\text{I}}_{\text{i}}=\frac{\text{n}({\text{Y}}_{\text{i}}-\overset{―}{\text{Y}})}{\sum_{\text{j}}{\left({\text{Y}}_{\text{j}}-\overset{―}{\text{Y}}\right)}^{\text{2}}}\sum_{\text{j}}{\text{w}}_{\text{ij}}\left({\text{Y}}_{\text{j}}-\overset{―}{\text{Y}}\right) \end{array}$$ (11)

The Local Moran I (and all other LISA values) are usually plotted to illustrate local associations, including high-high, high-low, low-high and low-low relationships with neighboring areas.

### 2.10 Getis-Ord Gi*

The Getis-Ord Gi* statistic is a local spatial statistic used to identify statistically significant spatial clusters of high values (hot spots) and low values (cold spots) [36]. It compares the value at each location with the values of its neighboring locations [34]. Areas surrounded by similarly high values are classified as hotspots, while areas surrounded by low values are classified as cold spots. It is calculated as [37]


$$\begin{array}{c} {\text{G}}_{\text{i}}^{*}=\frac{\sum_{\text{j}=\text{1}}^{\text{n}}{\text{w}}_{\text{ij}}{\text{Y}}_{\text{j}}-\overset{―}{\text{Y}}\sum_{\text{j}=\text{1}}^{\text{n}}{\text{w}}_{\text{ij}}}{\text{S}\sqrt{\frac{\text{n}{\sum_{\text{j}=\text{1}}^{\text{n}}{\text{w}}_{\text{ij}}}^{\text{2}}-{\left(\sum_{\text{j}=\text{1}}^{\text{n}}{\text{w}}_{\text{ij}}\right)}^{\text{2}}}{\text{n}-\text{1}}}} \end{array}$$ (12)

Here, ${\text{Y}}_{\text{j}}$ is the value at unit j from n spatial units

and ${\text{w}}_{\text{ij}}$ is the value of the spatial weight of unit i and j

and $\overset{―}{\text{Y}}$ is the sample mean

and $\text{S}=\sqrt{\frac{\sum_{\text{j}=\text{1}}^{\text{n}}{\text{Y}}_{\text{j}}^{\text{2}}}{\text{n}}-{\left(\;\overset{―}{\text{Y}}\right)}^{\text{2}}}$.

For each unit, the Gi* statistic is reported as a z-score [38]. Significant positive z-scores identify clusters of high values (hot spots), where higher z-scores denote stronger clustering [38]. Significant negative z-scores identify clusters of low values (cold spots), with more negative values indicating greater clustering intensity [38].

The Local Moran’s I statistic, cannot effectively discriminate between hot spots (i.e.,high-high clustering) and cold spots (i.e., low-low clustering) [39]. In contrast, the Getis-Ord Gi* statistic cannot identify spatial outliers [39]. Since the Getis-Ord Gi* focuses solely on hotspots and coldspots, it is generally easier for non-spatial analysts or non-statisticians to interpret compared to Local Moran’s I. However, Moran’s I is more powerful because it can distinguish both clusters and outliers.

### 2.11 Bivariate Local Moran’s I

The Bivariate Local Moran’s I identify localized spatial associations between two continuous numeric variables [40]. Unlike global measures, it detects statistically significant spatial correlations at individual spatial units [40]. The bivariate local Moran’s I at ith area is calculated as [41]


$$\begin{array}{c} {\text{I}(\text{i})}_{\text{xy}}=\frac{\left({\text{Y}}_{\text{i}}-\overset{―}{\text{Y}}\right)}{\frac{\sum_{\text{k}=\text{1}}^{\text{n}}{\left({\text{Y}}_{\text{k}}-\overset{―}{\text{Y}}\right)}^{\text{2}}}{\text{n}-\text{1}}}\sum_{\text{j}=\text{1}}^{\text{n}}{\text{w}}_{\text{ij}}\left({\text{X}}_{\text{j}}-\overset{―}{\text{X}}\right) \end{array}$$ (13)

Here, ${\text{Y}}_{\text{i}}$, ${\text{X}}_{\text{i}}$ are the values at unit i of the variables of interest,

$\overset{―}{\text{Y}}\;$and $\overset{―}{\text{X}}$ denote the respective means of the variables of interest.,

${\text{w}}_{\text{ij}}$ is the value of the spatial weight of unit i and j.

These spatial statistics were used to identify the spatial cluster high-high, low-low and spatial outliers high-low, low–high, where one variable is spatially correlated with another variable of the surrounding areas [42].

### 2.12 Bayesian hierarchical spatial models

A spatial model is a model that allows location information [43]. To analyze and interpret spatial patterns and relationships, Bayesian spatial models leverage statistical techniques that combine prior knowledge with the likelihood of observed data. The advantages of Bayesian approaches in spatial modeling lies in their hierarchical models. These models are structured in such a way that parameters at one level directly influence those at another, making them the most prevalent and powerful tools in spatial analysis. This analysis utilizes Poisson-Gamma (PG) model, Poisson-Lognormal (PLN) model, Conditional Auto Regression (CAR) model, Convolution model. The PG model serves as a baseline by accounting for extra-Poisson variation by capturing unstructured heterogeneity, while the PLN model provides greater flexibility when variability was substantial and not well captured by a single dispersion parameter although they are not including any spatial dependency. Then, a CAR model is introduced which allows the malaria risk in each district to be dependent on its neighboring districts through an adjacency structure. This was further expanded by the convolution model, which more clearly distinguishes between spatial dependency and random noise by combining unstructured district-specific heterogeneity with spatially structured effects. Zero-inflated versions of the CAR and convolution models were used to handle sparse data with a high number of zero counts. It allowed them to separate structural zeros from random case absences while maintaining spatial dependence.

### 2.13 Poisson-Gamma model

In real-life scenarios, it is often difficult for the mean and variance to be exactly equal indicating overdispersion, which highlights the limitations of using a Poisson model for count data. To address the issue of overdispersion, the negative binomial model is a useful alternative. In the context of spatial analysis, the negative binomial distribution can be viewed as a mixed model, as it incorporates random effects for each region, which are distributed according to the gamma distribution [44]. This model is well known as the Poisson-Gamma (PG) model [44].

According to this model, Malaria case counts in each district are assumed to be randomly distributed, following a Poisson distribution


$$\begin{array}{c} \text{i}.\text{e}.,\;{\text{y}}_{\text{i}}\sim \text{Poisson}\left({\text{e}}_{\text{i}}\theta_{\text{i}}\right)\text{ and }\theta_{\text{i}}\sim \text{Gamma}(\text{a},\text{b}) \end{array}$$ (14)

with the assumption that


$$\begin{array}{c} \lambda_{\text{i}}={\text{e}}_{\text{i}}\theta_{\text{i}};\;i=\text{1},\text{2},\ldots ,\text{6}\text{4} \end{array}$$ (15)

must be constant throughout each district [31]. The model is structured as a two-level model. Here, ei is the population in ${\text{i}}^{\text{th}}$ district and $\theta_{\text{i}}$ is the prevalence rate of malaria in that district which is assumed to be random and follow a gamma prior distribution with parameters a and b because of the existence of unobserved heterogeneity [31] i.e.,


$$\begin{array}{c} \theta_{\text{i}}\sim \text{Gamma}(\text{a},\text{b}) \end{array}$$ (16)

Following the result of the conjugacy between Poisson and gamma distributions [44],we can write


$$\begin{array}{c} \theta_{\text{i}}\sim \text{Gamma}(\text{a}+{\text{y}}_{\text{i}},\text{ b}+{\text{e}}_{\text{i}}) \end{array}$$ (17)

The main drawback of this model is its assumption that the data are distributed independently, which overlooks spatial correlation. The Poisson-gamma model has faced criticism for this limitation, as well as for the difficulty of incorporating additional variables into the model [44]. This has led to the development of more efficient models that take spatial correlation into account.

### 2.14 Poisson-Lognormal model

The Poisson- Lognormal (PLN) model is an alternative to the Poisson-Gamma (PG) model. Instead of assuming that prevalence rate follows a gamma distribution, in this model we assume that $\theta_{\text{i}}$ is associated with a linear predictor that has a normal distribution for its random effects component, ${\text{v}}_{\text{i}}$ [31]. The model is formulated as


$$\begin{array}{c} {\text{y}}_{\text{i}}\sim \text{Poisson }\left({\text{e}}_{\text{i}}\theta_{\text{i}}\right)\;;\text{ i}=\text{1},\text{2},\ldots ,\text{6}\text{4},\text{ and }\text{log}\theta_{\text{i}}=\alpha +{\text{v}}_{\text{i}} \end{array}$$ (18)

$$\begin{array}{c} {\text{v}}_{\text{i}}\sim \text{Normal}(\text{0},\sigma_{\text{v}}^{\text{2}}) \end{array}$$ (19)

α is the overall prevalence rate, which accounts for the extra Poisson fluctuation in the prevalence rate of malaria cases in area i [31].

The advantage of PLN over PG is that it can incorporate covariate easily, i.e.,


$$\begin{array}{c} \text{log}\theta_{\text{i}}=\alpha +{\text{x}}_{\text{i}}^{\prime }\beta +{\text{v}}_{\text{i}}\; \end{array}$$ (20)

Although spatial autocorrelation is not yet taken into consideration by this model, this drawback prompted the creation of more effective models that consider spatial correlation.

### 2.15 CAR model

Conditional Auto Regression is referred to by the acronym CAR [30]. Conditioning based on all the other is what the conditional in CAR represents [30]. Auto-regression means regression on itself [30].

CAR models incorporate a random-effects component that controls for spatial autocorrelation. The model is structured as follows


$$\begin{array}{c} {\text{y}}_{\text{i}}\sim \text{Poisson}\left({\text{e}}_{\text{i}}\theta_{\text{i}}\right)\text{ and }\text{log}\theta_{\text{i}}=\alpha +{\text{u}}_{\text{i}},\;{\text{u}}_{\text{i}}|{\text{u}}_{\text{j},\text{i≠j}}\sim \text{Normal}(\overset{―}{{\text{u}}_{\text{i}}},\sigma_{\text{i}}^{\text{2}}) \end{array}$$ (21)

$$\begin{array}{c} \overset{―}{{\text{u}}_{\text{i}}}=\frac{\text{1}}{\sum_{\text{j}=\text{1}}^{\text{n}}{\text{w}}_{\text{ij}}}\sum_{\text{j}=\text{1}}^{\text{n}}{\text{w}}_{\text{ij}}{\text{u}}_{\text{j}}\text{ and }\sigma_{\text{i}}^{\text{2}}=\frac{\sigma_{\text{u}}^{\text{2}}}{\sum_{\text{j}=\text{1}}^{\text{n}}{\text{w}}_{\text{ij}}} \end{array}$$ (22)

Here, the CAR is specified for the random effect ${\text{u}}_{\text{i}}$, i.e., each ${\text{u}}_{\text{i}}$ is conditional on the others ${\text{u}}_{\text{j}}$ for j = 1, 2, …, 64 but with j≠i [30]. The random effect is characterized by a normal distribution, where the mean and variance are weighted by adjacent regions using spatial weight matrix (defined by Queen contiguity in this analysis). As like PLN, this model can add covariate easily.

### 2.16 Convolution model

The convolution model is a modification of the CAR model. The modification made to the CAR model is that to incorporate a “nugget” effect, such as term ${\text{v}}_{\text{i}}$ [30]. In order to account for the spatial dependency, this model incorporates a spatial random effect ${\text{u}}_{\text{i}}$ and an unstructured exchangeable component ${\text{v}}_{\text{i}}$ to describe uncorrelated noise [45]. The model is structured as follows


$$\begin{array}{c} y_{i}\sim Poisson\left(e_{i}\theta_{i}\right)and\;log\theta_{i}=\alpha +u_{i}+v_{i},\;{\text{u}}_{\text{i}}|{\text{u}}_{\text{j},\text{i≠j}}\sim \text{Normal}\left(\overset{―}{{\text{u}}_{\text{i}}},\sigma_{i}^{2}\right),{\text{v}}_{\text{i}}\sim \text{Normal}(\text{0},\;\sigma_{v}^{2}) \end{array}$$ (23)

$$\begin{array}{c} \overset{―}{{\text{u}}_{\text{i}}}=\frac{\text{1}}{\sum_{\text{j}=\text{1}}^{\text{n}}{\text{w}}_{\text{ij}}}\sum_{\text{j}=\text{1}}^{\text{n}}{\text{w}}_{\text{ij}}{\text{u}}_{\text{j}}\text{and }\sigma_{\text{i}}^{\text{2}}=\frac{\sigma_{\text{u}}^{\text{2}}}{\sum_{\text{j}=\text{1}}^{\text{n}}{\text{w}}_{\text{ij}}} \end{array}$$ (24)

i.e., this model breaks down district-level random effects into correlated heterogeneity components ${\text{u}}_{\text{i}}$ and uncorrelated heterogeneity components ${\text{v}}_{\text{i}}$ which means that ${\text{u}}_{\text{i}}$ effects vary predictably between locations and assumed to follow a CAR model and ${\text{v}}_{\text{i}}$ indicates effects that vary randomly across locations and assumed to follow a normal distribution as like PLN model [31,44]. This model can also incorporate covariate easily. This analysis used queen-based contiguity for defining spatial weight matrix.

### 2.17 Zero inflated spatial models

To accommodate excess zeros while preserving spatial dependence, Zero-Inflated extensions of the CAR and convolution models were specified.

For Zero Inflated CAR model for district i, the number of malaria cases ${\text{y}}_{\text{i}}$ follows


$$\begin{array}{c} y_{i}\sim ZIP\left(e_{i}\theta_{i}\right)\;and\;log\theta_{i}=\alpha +u_{i},\;u_{i}|u_{j,i\text{≠}j}\sim CAR(\overset{―}{u_{i}},\sigma_{i}^{\text{2}}) \end{array}$$ (25)

For Zero Inflated Convolution model for district i, the number of malaria cases ${\text{y}}_{\text{i}}$ follows


$$\begin{array}{c} {\text{y}}_{\text{i}}\sim \text{ZIP}\left({\text{e}}_{\text{i}}\theta_{\text{i}}\right)\text{and}\text{log}\theta_{\text{i}}=\alpha +{\text{u}}_{\text{i}}+{\text{v}}_{\text{i}},{\text{u}}_{\text{i}}|{\text{u}}_{\text{j},\text{i≠j}}\sim \text{CAR}(\overset{―}{{\text{u}}_{\text{i}}},\sigma_{\text{i}}^{\text{2}}),\;{\text{v}}_{\text{i}}\sim \text{Normal}(\text{0},\;\sigma_{\text{v}}^{\text{2}}) \end{array}$$ (26)

We can easily add covariate here using the following equation,


$$\begin{array}{c} \text{log}\theta_{\text{i}}=\alpha +{\text{u}}_{\text{i}}+\text{X}\beta_{\text{i}},\text{for CAR} \end{array}$$ (27)

$$\begin{array}{c} \text{log}\theta_{\text{i}}=\alpha +{\text{u}}_{\text{i}}+{\text{v}}_{\text{i}}+\text{X}\beta_{\text{i}}\;,\text{ for Convolution} \end{array}$$ (28)

### 2.18 Prior specification and MCMC implementation

The variance parameters are defined as the inverse of the corresponding precision parameters, such that $\sigma_{u}^{\text{2}}=\text{1}/\tau_{u}\;$ and $\sigma_{v}^{\text{2}}=\text{1}/\tau_{v}$. Gamma hyperpriors were assigned to all precision parameters, e.g., $\tau_{U}\sim \text{Gamma}(\text{1},\text{0}.\text{01}),\;$ $\tau_{V}\sim \text{Gamma}(\text{1},\text{0}.\text{1})$ to allow weakly informative, data-driven estimation of spatial and unstructured variability consistent with standard Bayesian disease mapping practice [46]. In the MCMC implementation, precision parameters are sampled directly and the variance components are computed at each iteration as deterministic transformations. For the Poisson–Gamma model, Gamma hyperpriors were assigned (a = 4, b = 6) to preserve conjugacy in the hierarchical count model. Diffuse Normal priors were assigned to regression coefficients to reflect minimal prior information, Uniform(0,1) for the zero-inflation probability, and flat priors for intercepts.

All models were implemented in WinBUGS via R using the R2WinBUGS package. We used two MCMC chains with 660,000 iterations, a burn-in of 300,000, and thinning of 5 to reduce autocorrelation. Convergence and sampling adequacy were assessed through visual inspection of trace plots, Gelman–Rubin diagnostics, and calculation of the effective sample size for each parameter to ensure sufficient independent posterior samples.

The Gelman–Rubin statistic ($\hat{R}$) assesses chain convergence, with values below 1.1 indicating adequate convergence across chains [47]. Mixing efficiency and the impact of within-chain autocorrelation were evaluated using the effective sample size (n_eff), ensuring that n_eff was at least 5m (equivalent to a minimum of 10 effective draws per chain), as recommended by Bayesian Data Analysis [47].

### 2.19 Ethics statement

Since the data were obtained from Bangladesh Bureau of Statistics, obtaining individual consent was not required. Therefore, ethical approval was not applicable for this study.

## 3. Result and discussion

**Fig 2:**
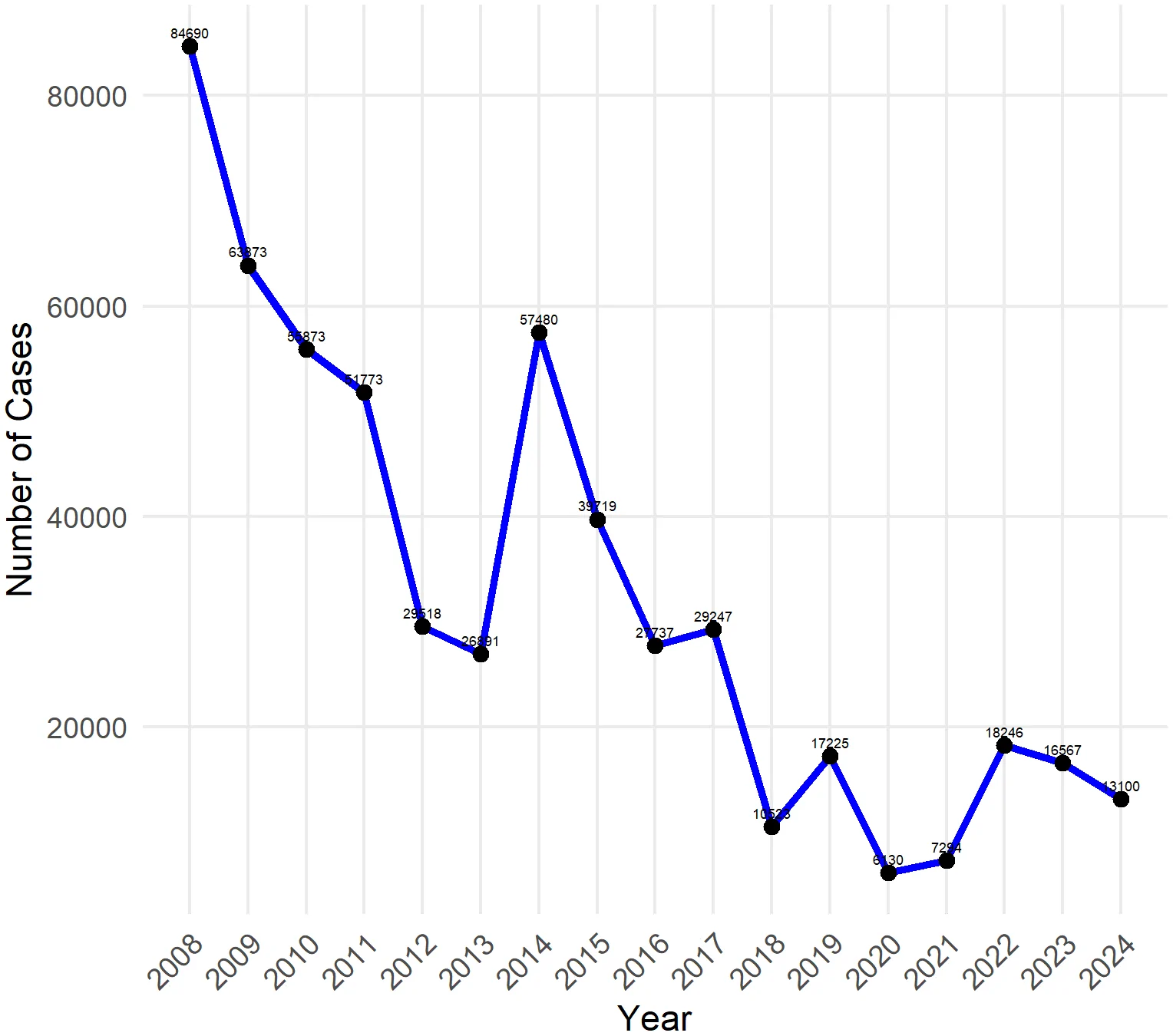
Annual confirmed malaria cases in Bangladesh reported by NMEP and WHO.

As shown in Fig 2, Malaria cases dropped steadily from 84,690 in 2008–26,891 in 2013, then spiked to 57,480 in 2014 before declining to 6,130 by 2020.

After a rise in 2021–2022, cases are now decreasing again, remaining below the 2014 peak. We then perform a two-sided Mann Kendall trend test and test of Sen’s slope estimator to determine if the decline is significant or not.

The negative τ value from Table 1 indicates a downward trend in the data over time, with a value of −0.691 suggesting a strong decrease. Additionally, the p-value is very small (< 0.001), indicating that the result is highly statistically significant. Therefore, we can reject the null hypothesis of no monotonic trend and conclude that there is a significant and strong decreasing monotonic trend in malaria cases over time in Bangladesh. The Sen’s slope estimator from Table 1 also indicates a decreasing trend in malaria cases over the years, with an estimated decline of approximately 3,779 cases per year. Moreover, the p-value is very small, suggesting that the result is statistically significant. This robust, non-parametric estimate confirms that annual malaria cases has steadily decreased despite short-term fluctuations.

**Table 1:** Mann–Kendall Trend Test and Sen’s slope estimator results for malaria cases.

Statistic	Value	p-value
Kendall’s τ	−0.691	<0.001
Sen’s slope	−3779.024	<0.001

**Fig 3:**
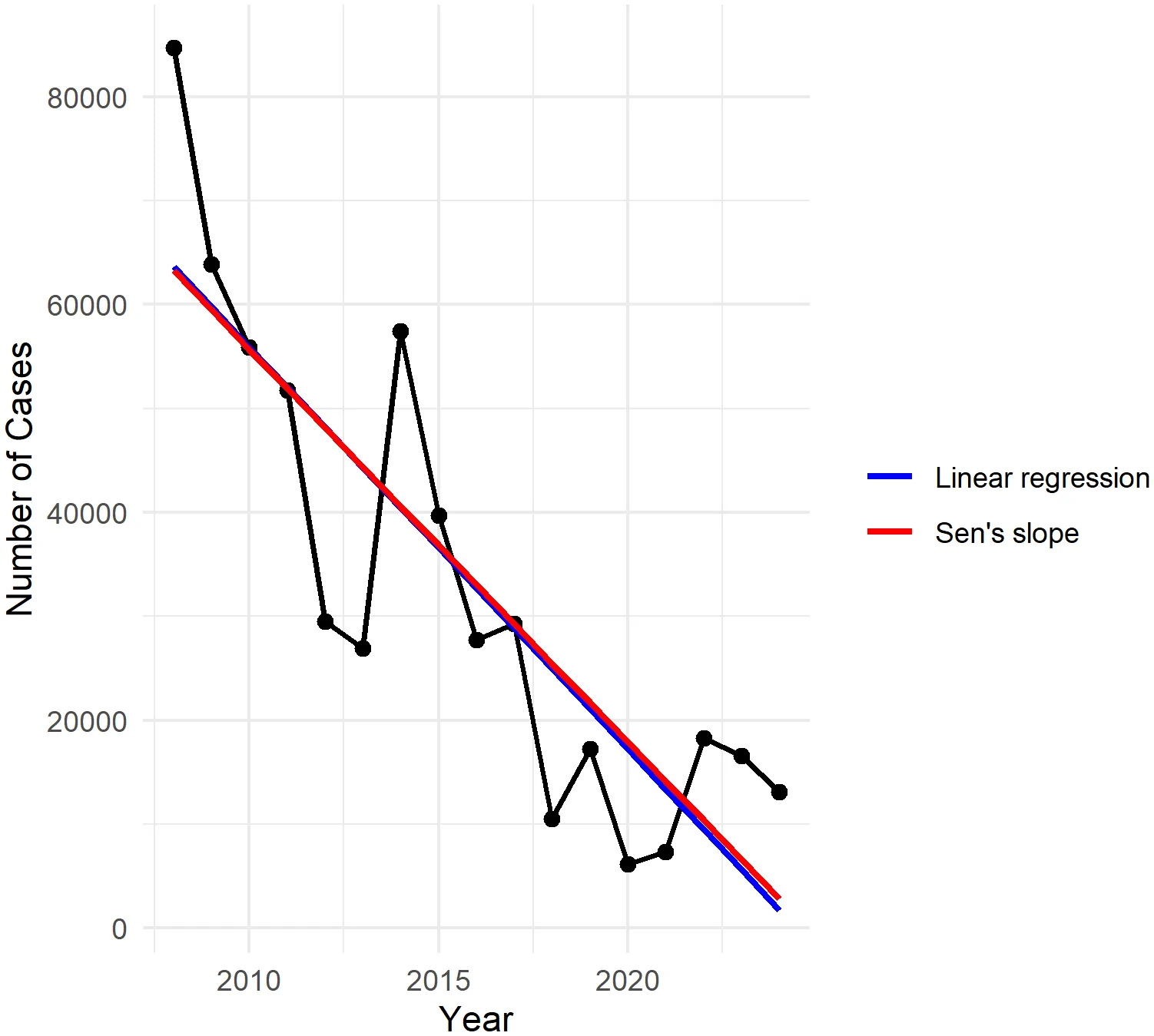
Trend in annual confirmed malaria cases over years. Raw data (black line), Linear regression (blue line), Sen's slope (red line).

Sen's slope estimator and linear regression in Fig 3 both show a significant declining trend in malaria cases over time. The strong declining trend appears to be unaffected by short-term fluctuations.

**Fig 4:**
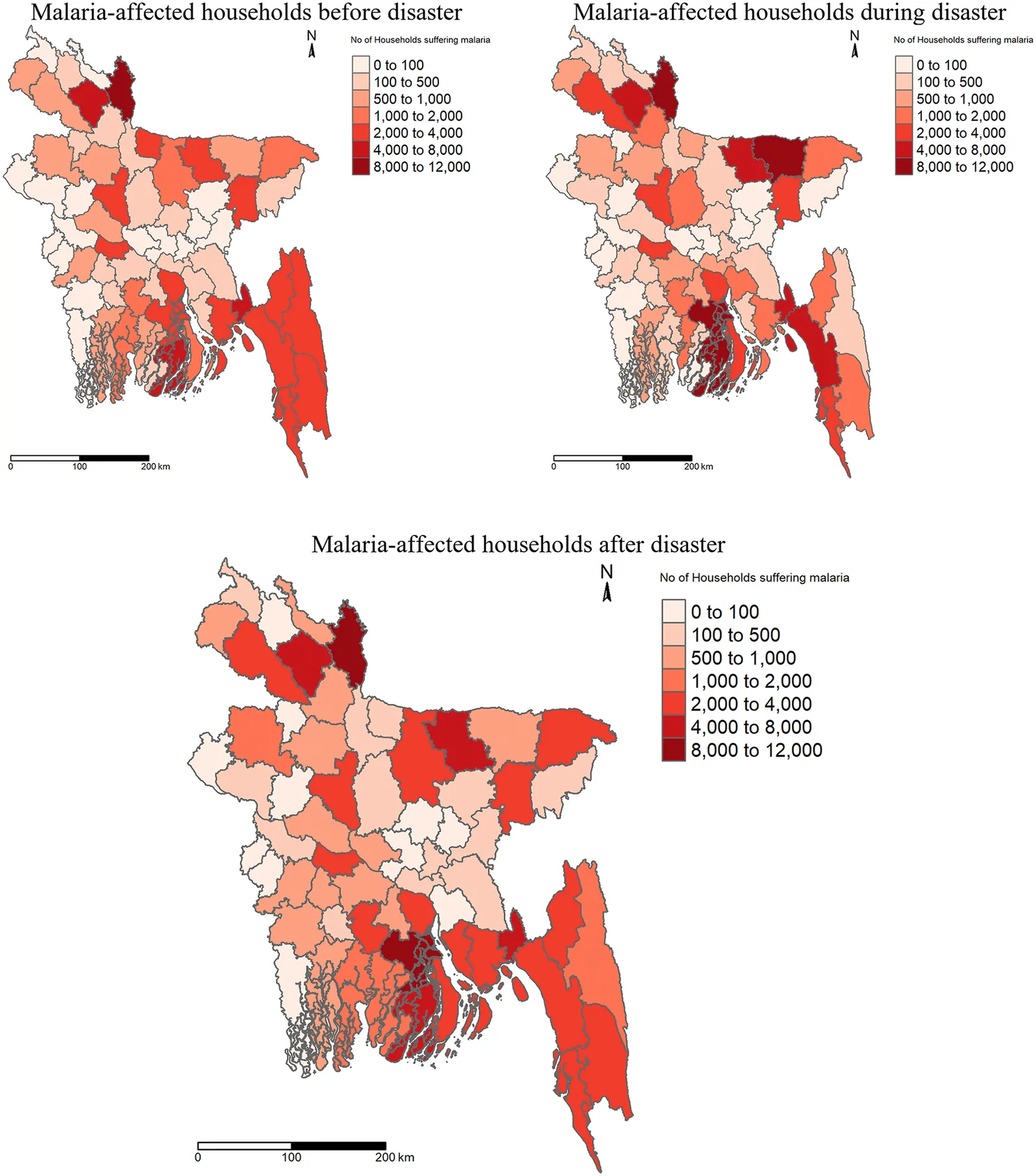
Malaria-affected households across pre, during, and post-disaster in Bangladesh. Administrative boundary data were obtained from the GADM (www.gadm.org), which permits the use of derived maps in academic publications under open licenses. Maps were generated by the authors using R. The figure is an original visualization and contains no proprietary basemap layers; it is compliant with CC BY 4.0 licensing.

Our analysis now focuses on the period from 2015 to 2020 in order to gain a clear understanding of the spatial dependency of reported malaria cases. Accordingly, we utilize the number of households affected by malaria before, during, and after the disaster illustrated in Fig 4.

Before the disaster (Fig 4, upper left), high malaria burden (2,000–4,000 affected households) was concentrated in the southeastern hill tracts, parts of the northeast, and some central and southern districts, including Bandarban, Rangamati, Khagrachhari, Cox’s Bazar, Chittagong, Barisal, Noakhali, Hobiganj, Netrakona, Shariatpur, Rajbari, Sherpur, and Sirajganj. Feni, Patuakhali, and Rangpur recorded higher burdens (4,000–8,000 households), while most other central and northern districts showed lower levels.

During the disaster (Fig 4, upper right), several districts—Bhola, Cox’s Bazar, Dinajpur, Hobiganj, Rajbari, Shariatpur, and Sirajganj—remained in the 2,000–4,000 range. Chittagong, Feni, Netrakona, and Rangpur experienced increased burdens (4,000–8,000 households), while Barisal

Kurigram, Patuakhali, and Sunamganj showed the highest impact (8,000–12,000 households).

After the disaster (Fig 4, bottom), districts with 2,000–4,000 affected households were dispersed across the northeast, central floodplains, coastal areas, and southeastern hills, including Bandarban, Bhola, Chittagong, Cox’s Bazar, Dinajpur, Hobiganj, Khagrachhari, Noakhali, Rajbari, Shariatpur, Sirajganj, and Sylhet. Higher burdens (4,000–8,000 households) persisted in Feni, Netrakona, Patuakhali, and Rangpur, while Barisal and Kurigram continued to exhibit the highest malaria burden (8,000–12,000 households). Other districts showed comparatively lower impacts. Notably, Cox’s Bazar, Hobiganj, Rajbari, Shariatpur, and Sirajganj consistently exhibited similar malaria burden levels (2,000–4,000 affected households) across all three maps.

It is worth noting that regions with a higher number of households affected by malaria are often located near one another, suggesting a potential spatial dependency. To explore this further, we will analyze the distribution of the population suffering from malaria, particularly due to disasters.

**Fig 5:**
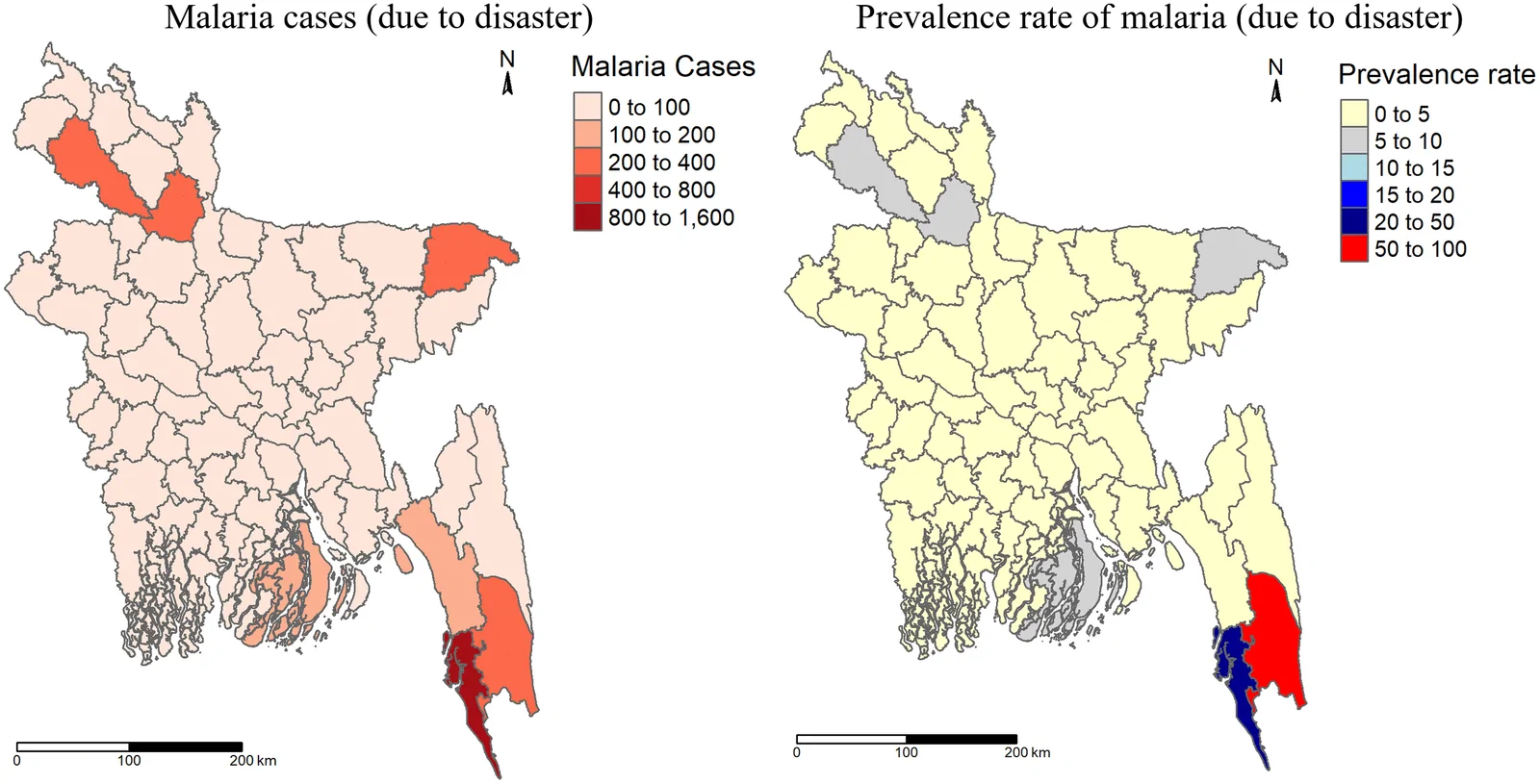
District-level malaria cases due to disaster(left) and prevalence rates of malaria due to disaster(right) in Bangladesh. Administrative boundary data were obtained from the GADM (www.gadm.org), which permits the use of derived maps in academic publications under open licenses. Maps were generated by the authors using R. The figure is an original visualization and contains no proprietary basemap layers; it is compliant with CC BY 4.0 licensing.

In (Fig 5,left) Pale beige regions correspond to areas with the lowest malaria incidence, reporting fewer than 100 cases. A gradual darkening of red shades indicates increasing malaria burdens, with mid-range districts reporting between 200 and 800 cases. Deep red regions, such as Cox’s Bazar and Bandarban, represent the highest malaria burden, making them the most severely affected districts in the country. Other regions like Sylhet, Dinajpur, Gaibandha, and Chittagong also show moderate to high case counts, indicated by medium to dark red shades, suggesting a significant public health concern, though less severe than the southernmost districts. The majority of the country, especially central and western districts, exhibit low or negligible malaria incidence, reflecting a geographically concentrated pattern of disease burden in the southeastern and northeastern regions.

(Fig 5,right) displays malaria prevalence rates, illustrating the proportion of affected individuals relative to the population within each district. The color palette ranges from light yellow to red, representing increasing rates of prevalence. Notably, Bandarban stands out as a significant hotspot, marked in bright red, followed closely by Cox's Bazar and Chittagong, which exhibit significant blue shading. In contrast to (Fig 5,left) this one provides a per capita perspective, highlighting the relative disease burden rather than merely the raw number of cases. This approach is particularly important for assessing health equity and resource allocation because it adjusts for population size, revealing areas that may be underserved or disproportionately affected, despite having smaller absolute case numbers. The maps reveal clusters of high malaria burden in southern Bangladesh, particularly in Bandarban, Cox's Bazar, and Chittagong. These districts are geographically connected, indicating positive spatial autocorrelation, where high prevalence areas are near others with similarly high rates (suspected hotspots). In contrast, central and western regions report consistently low cases. The southern hill tracts and coastal areas show both high case counts and high prevalence. This spatial pattern suggests that malaria is not randomly distributed but clustered in specific regions. To analyze this spatial dependency, Moran's I and Geary's C statistics are calculated using a queen-contiguity, row-standardized spatial weights matrix. And the results of these statistics are shown in Table 2.

**Table 2:** Moran I, Geary C and Getis-Ord General G statistic output under randomization.

Summary Statistic	Statistic	Expectation	Variance	p-value	Standard Deviate
Moran I	0.211	−0.016	0.002	<0.001*	4.651
Geary’C	0.601	1.000	0.028	<0.05*	2.400
Getis-Ord General G	0.217	0.071	0.004	<0.05*	2.308

N:B: p-value less than 0.05 and 0.001 is indicated by * and ** respectively.

Table 2 shows that the result of Global Moran I is 0.221 which indicates positive spatial autocorrelation for the number of people suffering from malaria due to disaster between districts. The observed spatial autocorrelation is statistically significant (p < 0.001), providing strong evidence against the null hypothesis of spatial randomness. The result of Geary C (from Table 2) is 0.601(<1) which signifies positive spatial autocorrelation for the number of people suffering from malaria due to disaster between districts. This value is statistically significant with a p-value less than 0.005 which suggests a significant deviation from randomness.

To assess hotspots, we utilize Getis-Ord General G using a queen-contiguity and binary spatial weights shown in Table 2. The Getis-Ord General G statistic value of 0.217 is significantly higher than its expected value of 0.071, along with a p-value of 0.0105. This suggests the presence of hot spots where districts exhibit a higher-than-expected concentration of malaria cases due to disasters, reinforcing the evidence of positive spatial correlation found with Moran I and Geary C. As the global spatial autocorrelation measures indicate the existence of clusters between the districts of malaria affected population, we also assess the local clustering.

**Fig 6:**
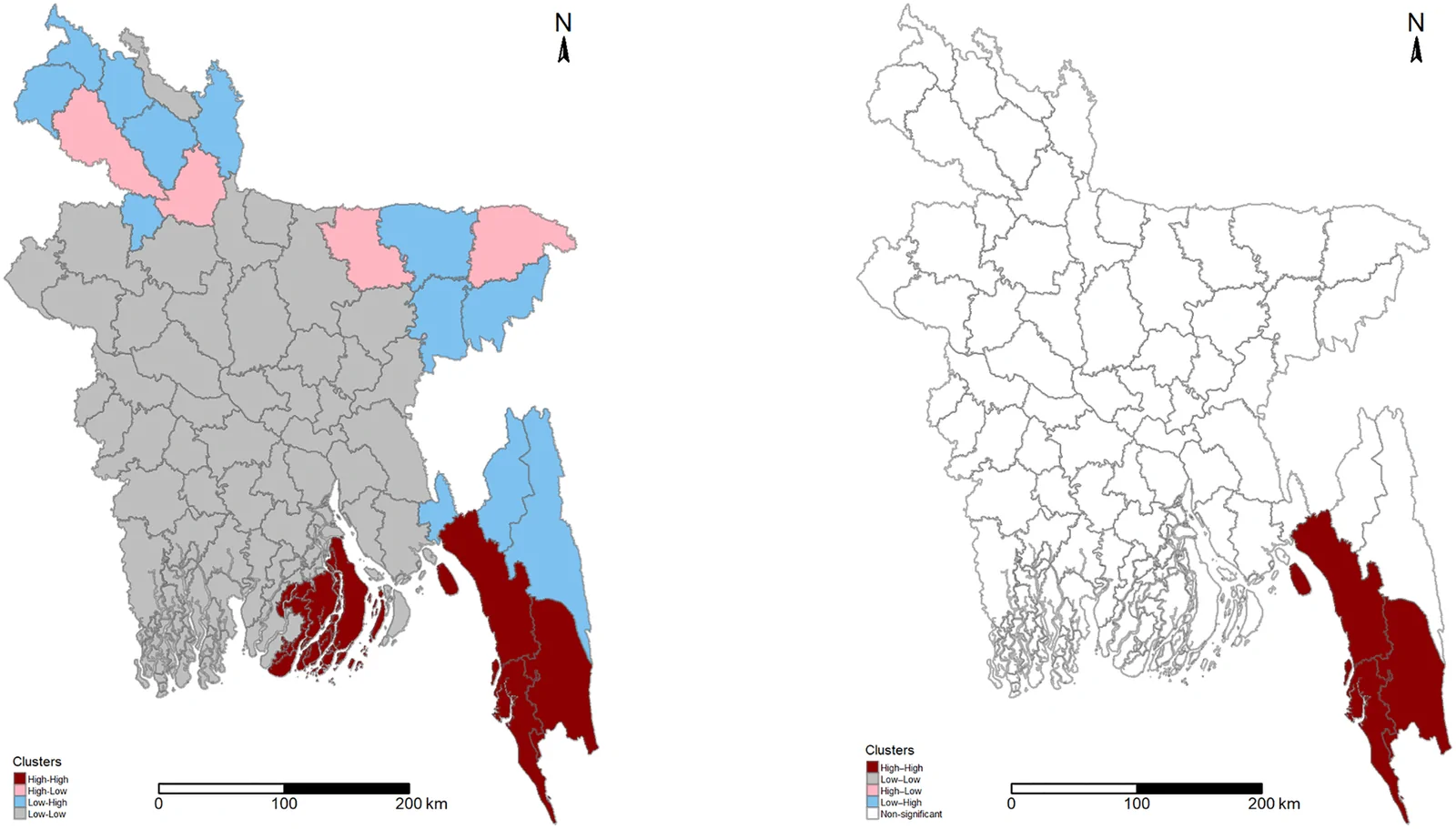
Local Moran’s I (LISA) cluster map of district-level malaria (due to disaster) vulnerability in Bangladesh. Red: (High–High), Gray: (Low–Low), Light Pink: (High–Low), Light Blue: (Low–High), white: (non-significant) districts. Administrative boundary data were obtained from the GADM (www.gadm.org), which permits the use of derived maps in academic publications under open licenses. Maps were generated by the authors using R. The figure is an original visualization and contains no proprietary basemap layers; it is compliant with CC BY 4.0 licensing.

The LISA cluster map using Local Moran's I is also being used to identify local spatial auto-correlation that is areas where values are similar (or dissimilar) to their neighbors. The maps are typically used in spatial analysis to identify clusters and outliers. In Fig 6 dark red color indicates a spatial cluster that is high-high which means that there are districts with high local Moran I value, ${\text{I}}_{\text{i}}$ surrounded by high local Moran I value, ${\text{I}}_{\text{i}}$ -strong positive spatial autocorrelation particularly in southern coastal and hilly regions i.e. Cox’s Bazar, Chittagong, Bandarban, Bhola, Patuakhali, Barguna. In Fig 6 gray color indicates a spatial cluster that is low-low which means that districts with low local Moran I value, ${\text{I}}_{\text{i}}$ surrounded by low local Moran I value, ${\text{I}}_{\text{i}}$ districts -another type of positive spatial autocorrelation particularly in central and western region of Bangladesh. In Fig 6 light pink color indicates a spatial outlier that is high-low which means that high local Moran I value, ${\text{I}}_{\text{i}}$ surrounded by low local Moran I value, ${\text{I}}_{\text{i}}$; while a light blue color also indicates a spatial outlier that is low-high which means that low local Moran I value, ${\text{I}}_{\text{i}}$ surrounded by high local Moran I value, ${\text{I}}_{\text{i}}$. These potential outliers are situated in the northern and north-eastern region of Bangladesh such as Sylhet, Sunamganj, Moulvibazar, Habiganj, Panchagarh, Nilphamari, Rangpur, Kurigram, Gaibandha, Joypurhat.

The right map of Fig 6 filters out non-significant cases and shows only statistically significant clusters which is only high-high clusters in this case, mainly concentrated in the southern districts (e.g., Cox's Bazar and surrounding areas). No low-low, high-low, or low-high clusters are shown, indicating they were not statistically significant at the chosen level of 0.05.

**Fig 7:**
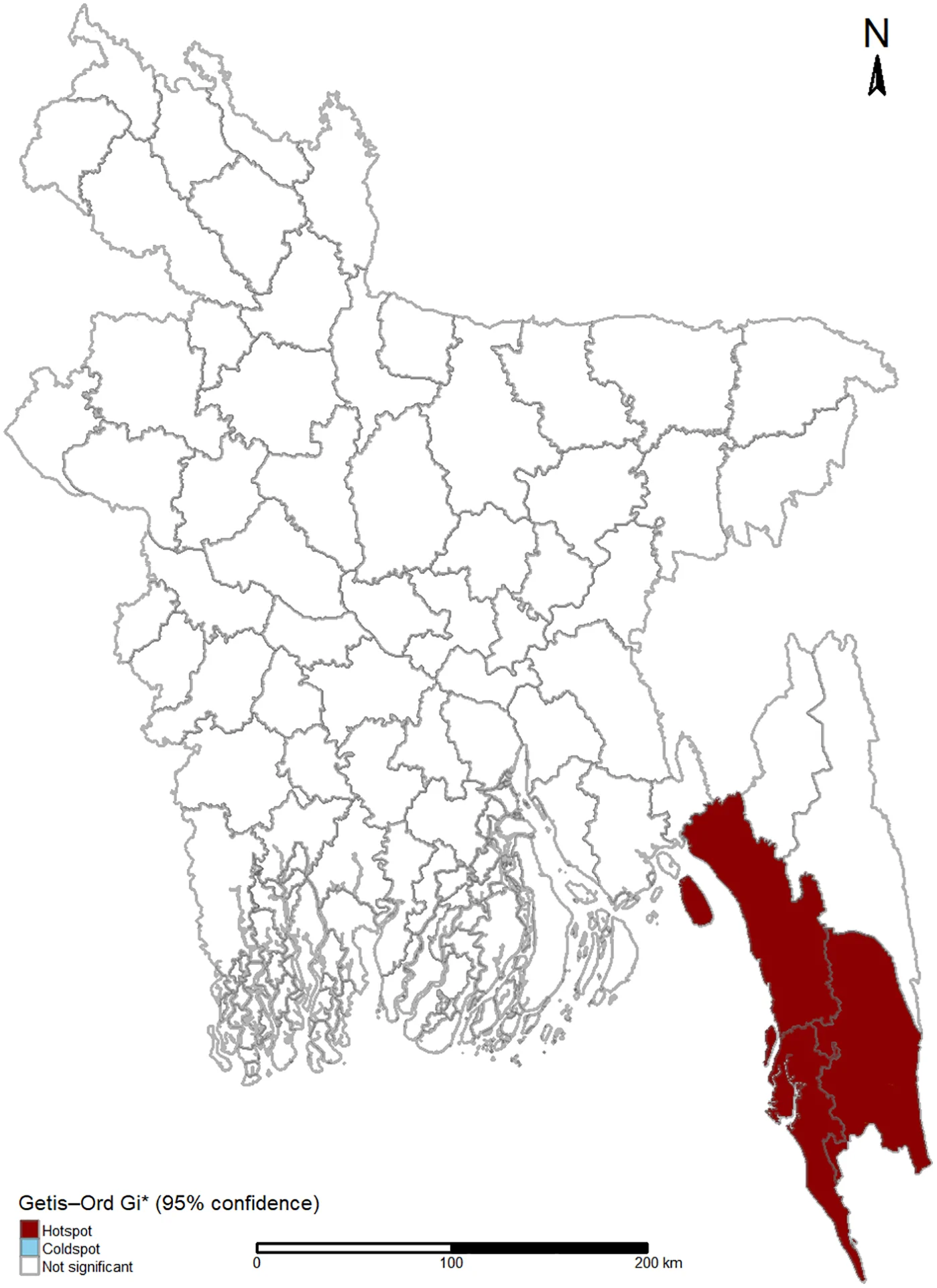
Getis-Ord Gi* cluster map of district-level malaria (due to disaster) vulnerability in Bangladesh. Red: (Hotspot), Light Blue: (Cold spot), white: (non-significant) districts. Administrative boundary data were obtained from the GADM (www.gadm.org), which permits the use of derived maps in academic publications under open licenses. Maps were generated by the authors using R. The figure is an original visualization and contains no proprietary basemap layers; it is compliant with CC BY 4.0 licensing.

We further investigate this local clustering using Getis-Ord Gi* statistic which is shown in Fig 7. This reveals a statistically significant spatial clustering of malaria cases due to disaster in southeastern Bangladesh, particularly in Cox’s bazar and Bandarban. These areas emerge as hotspots. However, the majority of districts in central, northern, and western Bangladesh show non-significant spatial patterns, and no statistically significant cold spots were found. And this result totally aligns with the local Moran’s I clustering. Prior to model specification, exploratory analysis of the district-level malaria counts was conducted. 82.8125% of districts reported zero cases, indicating potential zero inflation. Additionally, the variance (28920.13) of malaria counts exceeded the mean (44.84), suggesting overdispersion. The estimated zero-inflation probability, $\hat{\pi }$ is 0.817, which closely matches the observed proportion of zero counts (82.8%), confirming substantial zero inflation in the data.

Table 3 display the summary statistics of posterior estimates, including 95% credible interval. A credible interval implies that the true parameter would lie within the lower and upper limits, and we are 95% confident about this inclusion.

**Table 3:** Summary Statistics of Poisson-Gamma, Poisson-Lognormal, CAR, Convolution, CAR-ZIP, Convolution-ZIP models. Here, Variance components ($\sigma_{u}^{2},\sigma_{v}^{2}$) represent structured and unstructured variability, respectively. Precision parameters ($\tau_{u},\tau_{v}$) represent the corresponding precision terms, $p_{D}$ is the effective number of parameters, and DIC refers to the Deviance Information Criterion used for model comparison.

Posterior quantity	Poisson-Gamma	Poisson-Lognormal	CAR	Conv.	CAR_ZIP	Conv._ZIP
Shape parameter,$\hat{\text{a}}$	0.054 (1.00, 110000)	–	–	–	–	–
Rate parameter,$\hat{\text{b}}$	0.082 (1.00, 10000)	–	–	–	–	–
**Posterior mean of malaria(due to disaster)**	**44.79** (1.00, 10000)	**44.86 (1.00, 140000)**	**88.44 (1.02, 55000)**	**79.24 (1.00, 140000)**	**47.92 (1.00, 141506)**	**44.84 (1.00, 140000)**
variance	28850.0 (1.00, 140000)	–	–	–	–	–
overall model intercept,$\hat{\alpha }$	–	−9.04 (1.08, 89)	−11.56 (1.00, 20000)	−11.42 (1.29, 1300)	0.27 (1.00, 140000)	1.131 (1.00, 10000)
**Unstructured variance,** $\hat{\sigma_{𝐯}^{2}}$	**–**	**76.923**	**–**	**2.179** (1.98, 183)	**–**	**1.957 (1.00, 57000)**
Unstructured precision,$\hat{\tau_{\text{v}}}$	–	0.013 (1.02, 150)	–	7.484 (1.34, 2154)	–	0.661 (1.01, **57000)**
**Spatial variance,** $\hat{\sigma_{𝐮}^{2}}$	**–**	**–**	**189.400 (1.00, 140000)**	**10.600 (1.65, 10559)**	**6.451 (1.02, 140000)**	**0.229 (1.08, 1800)**
Spatial precision,$\hat{\tau_{\text{u}}}$	–	–	0.006 (1.00, 140000)	8.279 (1.18, 7596.631)	0.207 (1.00, 31457)	89.540 (1.00, 1800)
**95% Credible Interval(Mean)**	**(43.16,46.44)**	**(43.21,46.51)**	**(5.20,381.4)**	**(6.30,316.00)**	**(22.3387.67)**	**(43.22,46.5)**
Posterior mean deviance, $\overset{―}{\text{D}}$	92.0	90.3	5667.6	4481.7	266.3	86.348
Effective parameters, ${\text{p}}_{\text{D}}$	11.0	15.0	5592.2	4407.17	92031.6	11.400
**DIC**	**103.0**	**105.3**	**11259.8**	**8888.17**	**92297.8**	**97.748**

N.B. Bold values are used for the posterior mean, variance components ($\sigma_{u}^{\text{2}},\sigma_{v}^{\text{2}}$), 95% credible interval of posterior mean and DIC to improve readability. Values in parentheses after each estimate denote the Gelman–Rubin convergence diagnostic ($\hat{\text{R}}$) and the effective sample size (${\text{n}}_{\text{eff}}$). The CAR, Convolution, and CAR-ZIP models exhibited substantially higher DIC values and wider credible intervals compared to the Convolution-ZIP model, indicating poor fit for the sparse zero-inflated malaria data. These models are included for comparative evaluation only and are not recommended for inference. All conclusions are based on the Convolution-ZIP model.

It also shows the DIC values of six different models, two of which are non-spatial and others are spatial. Model comparison was conducted using the DIC, where lower values indicate better model fit. The calculation formula of DIC is in the appendix. Since DIC is composed of the posterior mean deviance ($\bar{D}$) and the effective number of parameters ($p_{D}$), differences in DIC reflect changes in both model fit and complexity and are therefore interpreted on a relative basis. As a rule of thumb, DIC differences greater than 5–10 are generally considered meaningful evidence favoring the model with the lower DIC [48]. The Convolution-ZIP model has lowest DIC value, exhibits stable convergence and adequate effective sample sizes. So, among the fitted models, Convolution-ZIP is the best model. This suggest that the sparse zero-inflated malaria data contains both correlated and uncorrelated heterogeneity and the fitted model is capable of capturing both types.

Convergence of the MCMC chains of convolution-ZIP model was also assessed using trace plots and kernel density plots which are in appendix. These plots support satisfactory convergence of the model.Due to authorized sign-in barriers, we did not have access to the original population affected by malaria. Instead, we utilized available data from the BBS, which shows the distribution of individuals suffering from malaria as the primary disease due to disaster incidents. This indicates that, in addition to the spatially correlated and uncorrelated heterogeneity, there may also be an impact from the disaster on the data. Therefore, it is essential to analyze this impact as well.From literature review we gain insight that, Anopheles mosquitoes require water to complete their life cycle. Therefore, it is relevant to consider disasters related to water, such as floods, cyclones, and storms. We will focus specifically on cyclones, as literature review indicates that the districts most affected by cyclones include Chittagong, Patuakhali, Barisal, Noakhali, and Khulna [49]. This pattern exhibits the same pattern with the population of people suffering from malaria due to disaster, which are also reported high in the southern coastal and hilly regions. However, due to the unavailability of consistent district-level data on cyclone frequency across all 64 districts, we use rainfall as a proxy measure of cyclone exposure. We acknowledge that rainfall captures only one dimension of cyclone impact and does not fully represent storm intensity, duration, timing. Here, we employ annual district-level average rainfall (in millimeters) obtained from the Bangladesh Water Development Board for the period 2017–2020 and aggregate these values across years to construct a cumulative rainfall measure. Districts with higher cumulative rainfall during this period are assumed to have experienced greater cyclone-related exposure.

**Fig 8:**
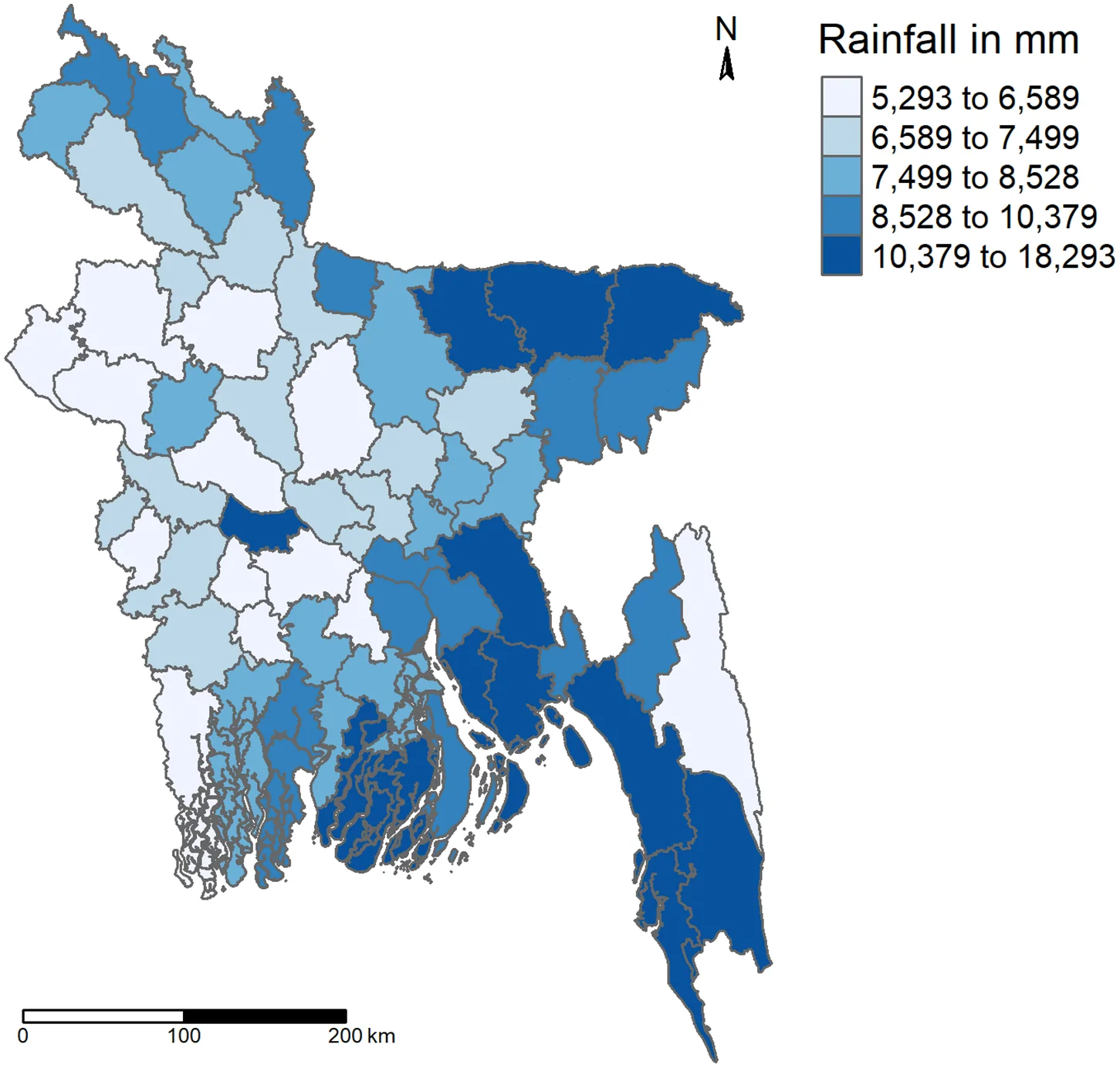
Spatial distribution of average annual rainfall (mm) across districts of Bangladesh. Administrative boundary data were obtained from the GADM (www.gadm.org), which permits the use of derived maps in academic publications under open licenses. Maps were generated by the authors using R. The figure is an original visualization and contains no proprietary basemap layers; it is compliant with CC BY 4.0 licensing.

**Fig 9:**
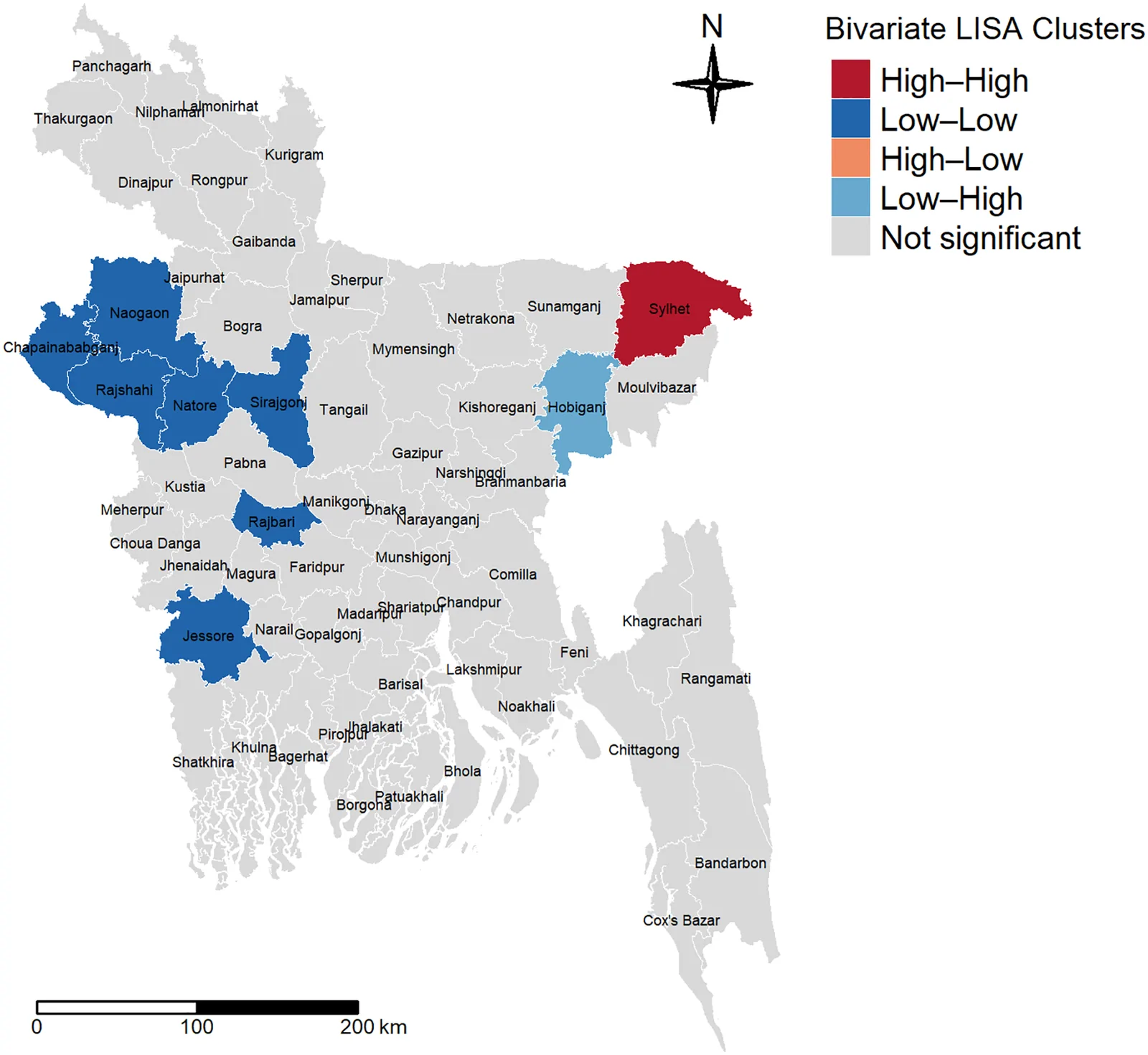
Bivariate LISA map of malaria due to disaster and rainfall across districts of Bangladesh. Red: (High–High), Blue: (Low–Low), Light orange: (High–Low), Light Blue: (Low–High), off-white: (non-significant) districts. Administrative boundary data were obtained from the GADM (www.gadm.org), which permits the use of derived maps in academic publications under open licenses. Maps were generated by the authors using R. The figure is an original visualization and contains no proprietary basemap layers; it is compliant with CC BY 4.0 licensing.

Fig 8 shows the district-wise total of average rainfall (in millimeters) classified into five categories. Rainfall is highest in the northeastern districts of Sylhet and Sunamganj as well as in the southeastern districts of Chittagong Cox’s Bazar, and Bandarban, and across several southern coastal districts, including Bhola, Patuakhali, and Barguna. Notably, some of these high-rainfall districts also report high malaria cases due to disasters, suggesting a potential spatial association. This hypothesis is further examined using a Bivariate LISA analysis, as shown in Fig 9.

Fig 9, a distinct High–High cluster in the northeast (Sylhet) highlights a strong association between high rainfall and increased malaria cases due to disaster. This pattern shows a correlation between how heavy rainfall creates mosquito breeding habitats and elevates post-disaster exposure. Low–Low clusters dominate the western and southwestern districts (e.g., Rajshahi, Naogaon, Natore, Sirajganj, Jessore), indicating regions where both rainfall and disaster-related malaria remain low. These areas experience drier conditions and consequently face a lower transmission risk. Low–High clusters in parts of the east-central region (Hobiganj) indicate lower malaria cases despite being surrounded by high-rainfall districts. The absence of High–Low clusters suggests that high malaria cases due to disaster rarely occurs in low-rainfall environments, reinforcing rainfall as an important driver of disaster-related malaria risk.

So we incorporate the rainfall as a covariate. Table 4 shows the Convolution model with Zero Inflated Poisson likelihood without and with covariate total of average rainfall (in mm).

**Table 4:** Summary statistics of convolution ZIP model without and with covariate. Here, Variance components ($\sigma_{u}^{2},\sigma_{v}^{2}$) represent structured and unstructured variability, respectively, $p_{D}$ is the effective number of parameters, and DIC refers to the Deviance Information Criterion used for model comparison.

Models	Mean	$\hat{\alpha }$	$\hat{\beta }$	$\hat{\sigma_{v}^{2}}$	$\hat{\sigma_{u}^{2}}$	$\overset{―}{D}$	$p_{D}$	DIC
Conv_ZIP((without covariate)	44.84 (1.00, 140000)	1.131 (1.00, 10000)	–	1.957 (1.00, 57000)	0.229 (1.08, 1800)	86.35	11.4	97.748
Conv_ZIP(with covariate)	44.84 (1.00, 140000)	−0.872 (1.02, 2200)	1.875 (1.01, 34000)	1.613 (1.07, 2300)	0.103 (1.02, 3400)	86.46	11.5	97.970

N.B. Values in parentheses after each estimate denote the Gelman–Rubin convergence diagnostic ($\hat{\text{R}}$) and the effective sample size (${\text{n}}_{\text{eff}}$).

The estimated coefficient for total average rainfall is positive, indicating that higher rainfall is associated with increased disaster-related malaria risk. However, inclusion of rainfall in the convolution Zero-Inflated Poisson model resulted in a marginal increase in DIC (ΔDIC = 97.970 − 97.748 ≈ 0.2), suggesting no meaningful improvement in overall model fit. The minimal difference in DIC indicates that both models provide essentially comparable explanatory performance.

Although the bivariate LISA analysis identified localized spatial clustering between rainfall and disaster-related malaria, the regression results suggest that much of this spatial association may already be accounted for by the structured and unstructured spatial random effects. Therefore, rainfall does not emerge as a strong independent global predictor within the spatial modeling framework.

**Fig 10:**
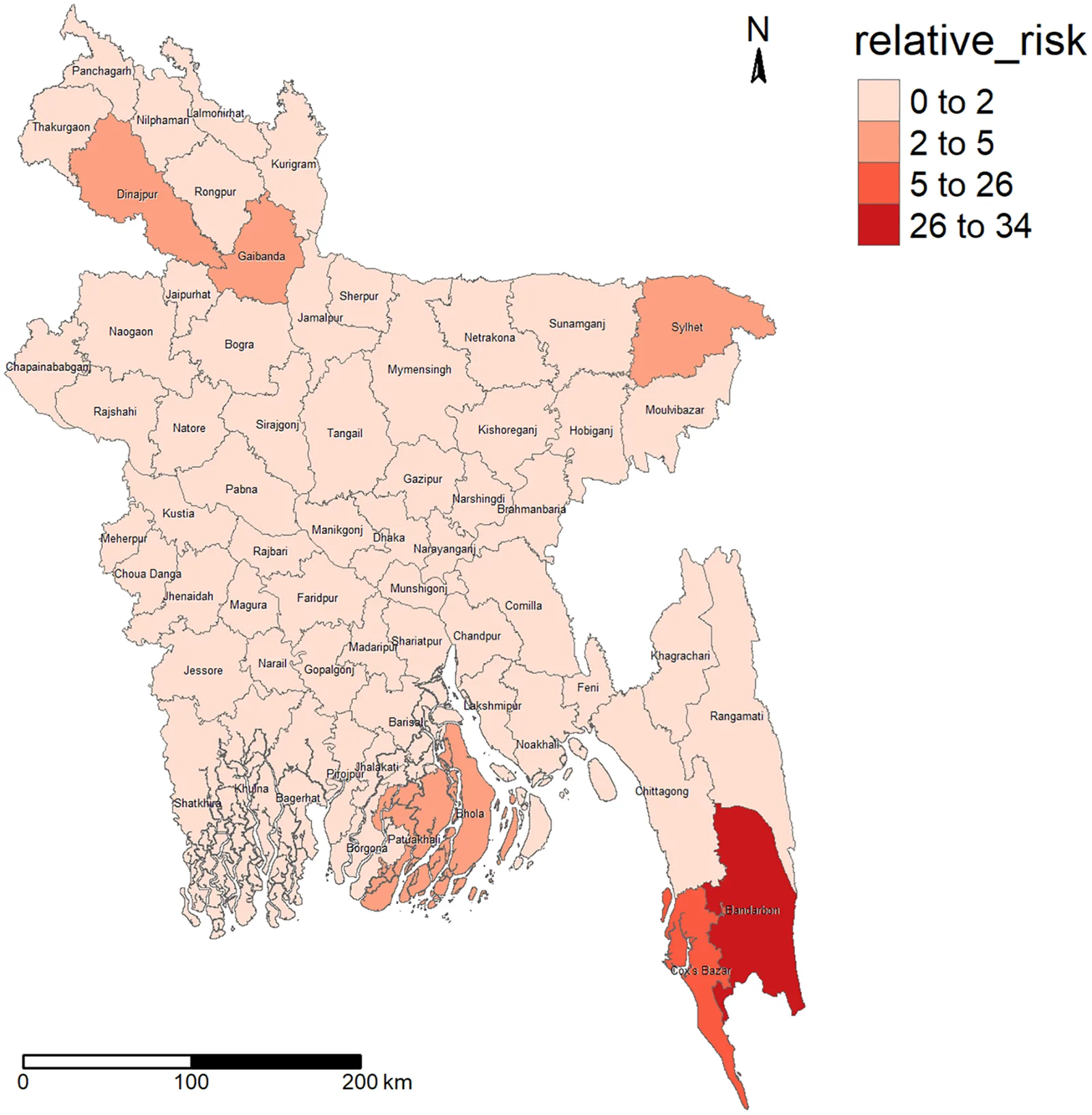
Mapping (Relative Risk) of Malaria due to disaster using Conv. ZIP model. Administrative boundary data were obtained from the GADM (www.gadm.org), which permits the use of derived maps in academic publications under open licenses. Maps were generated by the authors using R. The figure is an original visualization and contains no proprietary basemap layers; it is compliant with CC BY 4.0 licensing.

As the model without rainfall covariate has lower DIC (i.e., it outperformed other models), it is used for mapping the relative risk of malaria across all districts of Bangladesh. For mapping, we used the ratio of malaria case in each district predicted by the Zero-Inflated Poisson model and the expected malaria case in each district and showed it in Fig 10. In **Fig *10*** light beige (0–2) represents very low malaria risk, covering most central, western, and southwestern districts of Bangladesh. Light orange (2–5) indicates low-to-moderate risk across several northern and coastal districts, including Barguna, Patuakhali, Bhola, Sylhet, Dinajpur, and Gaibandha, suggesting localized but noticeable malaria transmission in these areas. Orange (5–26) reflects moderate risk particularly in Cox’s Bazar**.** Dark orange to dark red (26–34) marks high to extremely high malaria risk, concentrated primarily in Bandarban, the principal malaria hotspots in the country.

The southeastern hill tracts, particularly Cox’s Bazar and Bandarban, are the primary malaria hotspots.

**Fig 11:**
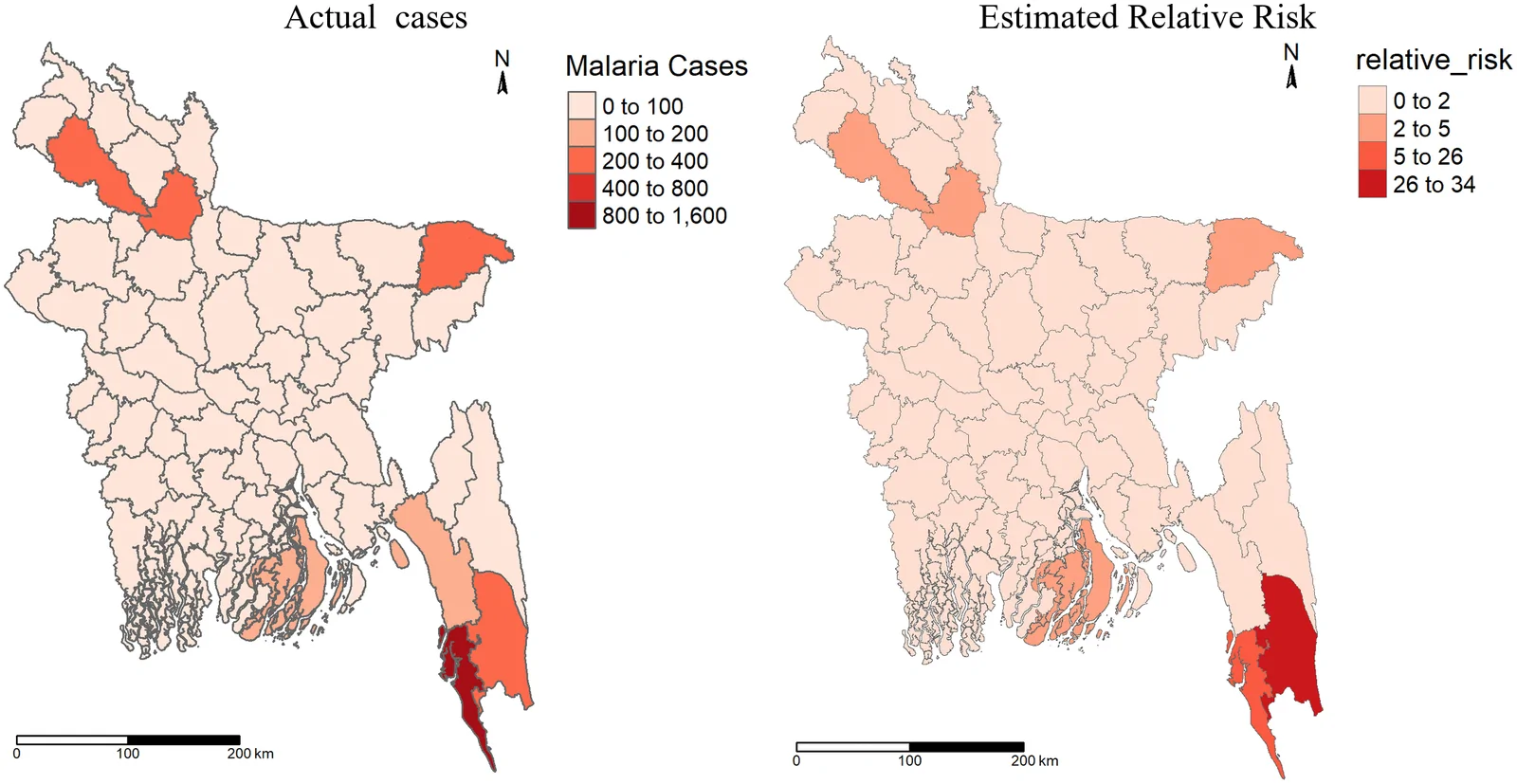
Comparison of Actual Malaria case and predicted Mapping (Relative Risk) of Malaria. Administrative boundary data were obtained from the GADM (www.gadm.org), which permits the use of derived maps in academic publications under open licenses. Maps were generated by the authors using R. The figure is an original visualization and contains no proprietary basemap layers; it is compliant with CC BY 4.0 licensing.

A side-by-side comparison (Fig 11) of the actual malaria cases and the risk mapping shows that the estimated relative risk closely matches the observed data. This demonstrates that our model provides an excellent fit and accurately captures the underlying pattern in the data. We confidently identify low-risk and high-risk areas based on their relative risk confidence intervals.

**Fig 12:**
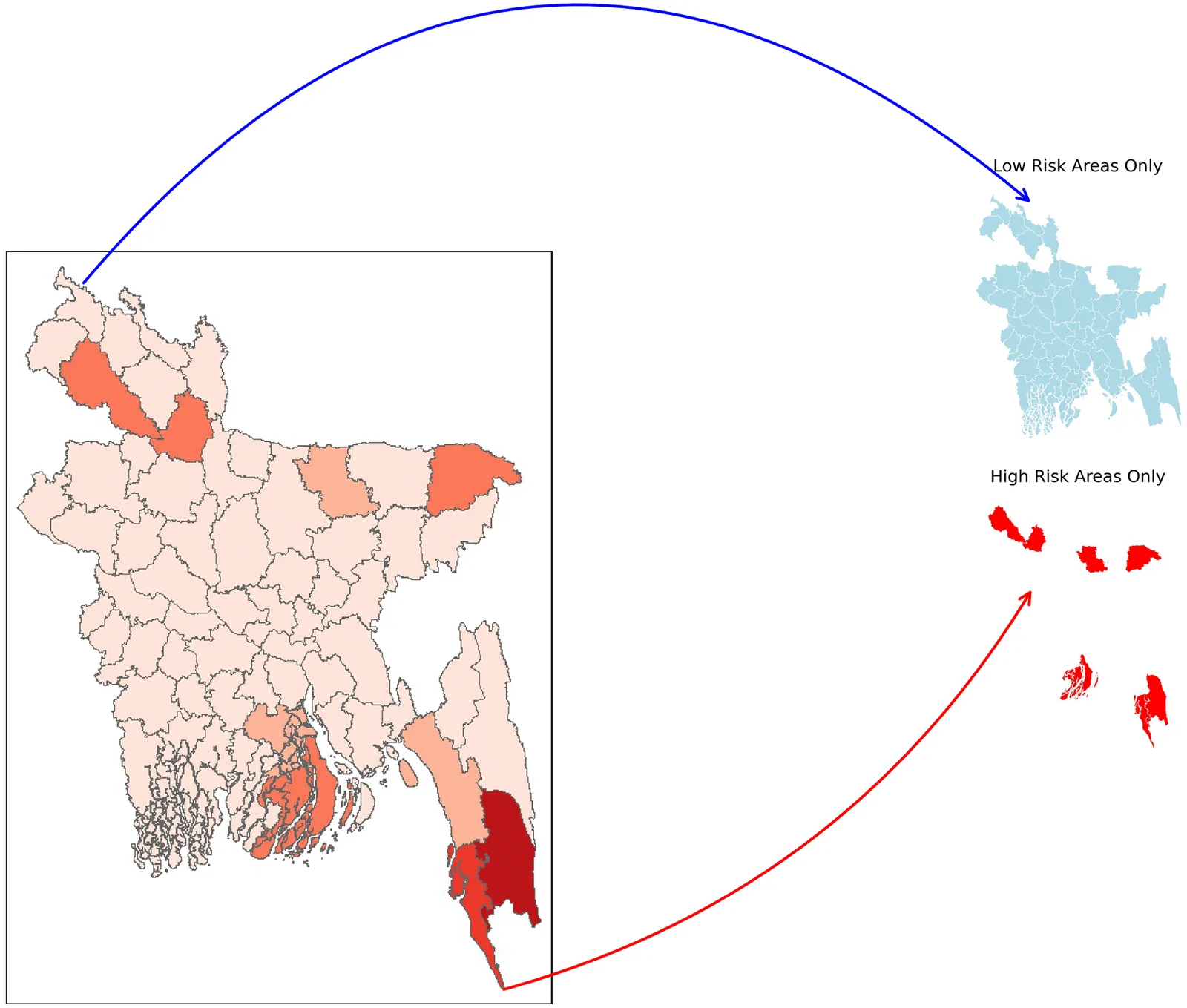
High and Low risk areas of malaria mapping. Administrative boundary data were obtained from the GADM (www.gadm.org), which permits the use of derived maps in academic publications under open licenses. Maps were generated by the authors using R. The figure is an original visualization and contains no proprietary basemap layers; it is compliant with CC BY 4.0 licensing.

Districts were classified based on their 95% credible intervals for relative risk. Areas were considered high risk when the lower bound of the credible interval exceeded 1 (LCL > 1), and low risk when the upper bound of the credible interval was below 1 (UCL < 1). Districts whose credible intervals included 1 were regarded as not significantly different from the baseline risk. Fig 12 clearly illustrates this classification, which is crucial for effective intervention strategies. We identify 54 districts as low-risk areas and 8 districts as high-risk areas and 2 district as not significant from baseline risk. The relative risks and confidence intervals for high-risk areas are detailed in **Table 5** which are necessary for policy intervention.

**Table 5:** Relative risk and confidence interval of high-risk areas.

Area	Relative Risk (LCL,UCL)	Area	Relative Risk (LCL,UCL)	Area	Relative Risk (LCL,UCL)
Bandarbon	34.36 (30.49,38.44)	Cox's Bazar	25.67 (24.26,27.10)	Netrakona	1.70 (1.33,2.13)
Bhola	4.35 (3.67,5.08)	Dinajpur	4.60 (4.06,5.17)	Patuakhali	3.67 (3.01,4.38)
Gaibanda	4.70 (4.09,5.36)	Sylhet	4.64 (4.14,5.16)		

The central map (Fig 12, left) shows the district-wise malaria risk across Bangladesh. The two side maps provide a zoomed-in view, highlighting low-risk and high-risk areas. This figure is designed to clearly distinguish and spatially separate high-risk and low-risk districts for malaria in Bangladesh. These visualizations will help guide targeted public health interventions, improve resource allocation, and focus preventive strategies on high-burden regions of relative malaria risk.

## 4. Conclusion

This nationwide spatial analysis shows that the malaria burden in Bangladesh has declined overall, and this declining trend is statistically significant. However, despite this national progress, specific districts continue to exhibit elevated relative vulnerability. Persistent high-risk clusters were identified primarily in the southeastern hill districts and in selected northern areas, indicating that spatial heterogeneity remains even as the country advances toward malaria elimination goals.

The Global Moran’s I value of 0.211 and Geary’s C value of 0.601 indicate a statistically significant positive spatial autocorrelation (moderate). This clustering suggests that geographically targeted interventions may be more effective than uniform national strategies. The Getis–Ord General G statistic value of 0.217 further suggests clustering of high values (hotspots), meaning that districts with elevated malaria vulnerability are spatially concentrated.

Local Indicators of Spatial Association (Local Moran’s I and Getis–Ord Gi*) identified clear high–high clusters and hotspots in the southeastern region, particularly in Cox’s Bazar and Bandarban. These findings are consistent with the well-documented concentration of malaria in the Chittagong Hill Tracts (CHT), where ecological suitability, forest proximity [50], and cross-border movement [51] contribute to sustained transmission risk.

Although we identified the presence of spatial dependency, we fitted both non-spatial and spatial models to better understand and quantify this structure. The purpose of including non-spatial models was to demonstrate the comparative advantage of spatial modeling. Anticipating that progress toward malaria elimination would lead to an increasing number of zero counts in district-level data, we employed a Zero-Inflated Poisson (ZIP) likelihood for posterior estimation. As expected, the spatial convolution model with ZIP likelihood outperformed the alternative specifications. This confirms that even modest spatial autocorrelation can meaningfully influence model performance when spatially structured and unstructured heterogeneity are properly accounted for.

Because the study used data on the distribution of population suffering from malaria during disaster contexts, we attempted to capture environmental influence using rainfall as a covariate. Rainfall showed a positive association with malaria vulnerability, consistent with established evidence that increased rainfall creates favorable breeding conditions for Anopheles mosquitoes and often precedes seasonal peaks in malaria incidence [52,53]. However, model comparison based on DIC indicated that inclusion of rainfall did not improve overall model fit; in fact, the model without rainfall yielded a lower DIC value. This suggests that rainfall, when considered alone and within the current spatial framework, explains limited additional variation beyond that already captured by spatial random effects. Therefore, while rainfall remains biologically plausible as a contributing factor, its independent contribution in this spatial model appears modest.

Our findings of persistent high-risk clusters in southeastern and selected northern districts align with prior evidence that malaria in Bangladesh exhibits marked spatial heterogeneity, with stable hotspots in the Chittagong Hill Tracts over multiple years despite overall declines in transmission [9,12]. Previous hotspot analyses have similarly identified the CHT and adjacent districts as consistently high-burden zones and recommended intensified control efforts to interrupt residual transmission. Ecological and occupational exposures, including forest-based livelihoods and slash-and-burn (jhum) cultivation practices, further contribute to elevated exposure risk in these regions [10]. As national case counts decline, a relatively small number of districts account for a substantial share of the remaining burden. These findings support geographically targeted strategies—such as intensified surveillance, active case detection, vector control, and community engagement—to address residual vulnerability in high-risk zones. This approach is consistent with the NMEP’s stratified elimination framework, which prioritizes intensified interventions in high-transmission districts—particularly in the Chittagong Hill Tracts—while progressively transitioning lower-burden districts toward interruption of local transmission and maintenance of non-endemic status [1]. The spatial clustering identified in this analysis reinforces the relevance of such phased and geographically focused strategies as Bangladesh advances toward malaria elimination goals.

This study has important limitations related to data availability. Because routine district-level malaria surveillance data from the NMEP were not publicly accessible, this analysis relied on disaster-associated malaria burden reported by BBS as a proxy measure. Although this dataset provides valuable insight into geographic vulnerability, it does not directly measure confirmed malaria transmission intensity and may underrepresent asymptomatic or non-disaster-related cases. The use of aggregated district-level data also limits ecological inference and masks finer spatial heterogeneity within districts. For these reasons, improved public access to routine surveillance data is essential for future research. Future research should incorporate climatic extremes, human mobility data, and spatiotemporal Bayesian modeling frameworks to better capture dynamic transmission processes and validate spatial risk patterns.

Because the outcome variable reflects disaster-associated malaria burden rather than confirmed surveillance-based transmission incidence, the findings should not be interpreted as direct measures of malaria transmission intensity. Instead, the results identify districts with relatively higher vulnerability under the available data framework. While these spatial patterns are relevant for informing geographically targeted strategies, caution is warranted when linking them directly to transmission dynamics or elimination thresholds.

The National Strategic Plan for Malaria Elimination emphasizes geographically stratified and phased intervention in recognition of malaria’s increasingly focal nature in Bangladesh, particularly in the Chittagong Hill Tracts. The spatial heterogeneity identified in this analysis is consistent with that strategic direction. By demonstrating that a limited number of districts continue to exhibit elevated vulnerability despite overall national decline, this study reinforces the rationale for intensified action in high-risk areas alongside sustained surveillance in lower-burden districts. Continued implementation of such geographically targeted and evidence-based approaches will be critical to consolidating gains achieved to date and advancing Bangladesh’s malaria elimination goals in a focused and sustainable manner.

## Supporting information

S1 Appendix Supplementary methods and Bayesian model diagnostics. (PDF)
