# Supplementary material for: Spatial Analysis of Malaria in Bangladesh: Insights from Bayesian Disease Mapping Models
Source: PLoS One. 2026 Aug 24;21(8):e0353483. doi: 10.1371/journal.pone.0353483 (PMC13502707; doi:10.1371/journal.pone.0353483)
Supplement: Supplementary file 1 [file pone.0353483.s001.pdf]

S1 Appendix. Supplementary methods and Bayesian model diagnostics.

A. Mann Kendall Trend test

The hypothesis under this test is

H_0: There is no monotonic present and

H_1: There is monotonic trend present

The steps to conduct the test is

Step-1: List the data in chronological order $(y, y_2, \dots, y_n)$ collected specifying the measurements taken at each time point $t_1, t_2, \dots, t_n$

Step-2: Determine the sign of all $\frac{n*(n-1)}{2}$ possible differences $y_j - y_k$ where j > k.

Step-3: Let sign $(y_j - y_k)$ be an indicator function that takes on the values -1, 0, 1, i.e.,

$$\mathrm{sign}(y - y_k) = \begin{cases} 1 \ \text{if } y_j > y_k \\ 0 \ \text{if } y_j = y_k \\ -1 \ \text{if } y_j < y_k \end{cases}$$

Step-4: Compute

$$S = \sum_{k-1}^{n-1} \sum_{j-k+1}^{n} \mathrm{sign}(y_j - y_k)$$

which is the number of positive differences minus the number of negative differences. If S is a positive number, observations obtained later in time tend to be larger than observations made earlier. If S is a negative number, then observations made later in time tend to be smaller than observations made earlier.

Step-5: As $n > 10$ in this case, compute the variance of S as

$$\mathrm{Var}(S) = \frac{1}{18}\left[n(n-1)(2n+5) - \sum_{p-1}^{g} t_p(t_p - 1)(2t_p + 5)\right]$$

Here, g is the number of tied groups and t_p is the number of observations in the pth group.

Step-6: Now, compute the Mann-Kendall test statistic Z_{MK} as

$$z_{MK} = \begin{cases} \frac{S-1}{\sqrt{Var(S)}} \text{ if } S > 0 \\ 0 \text{ if } S = 0 \\ \frac{S+1}{\sqrt{Var(S)}} \text{ if } S < 0 \end{cases}$$

A positive (negative) value of Z_{MK} indicates that the data tend to increase(decrease) with time.

Step-7: Reject the null hypothesis and conclude that upward, downward or general monotonic trend is present if $z_{MK} \geq z_{1-\alpha}$, $Z_{MK} \leq -Z_{1-\alpha}$, $|Z_{MK}| \geq Z_{1-\frac{\alpha}{2}}$ respectively. Here, α is the probability of rejecting the null hypothesis when it is true and $Z_{1-\alpha}$ is the $100(1-\alpha)^{th}$ percentile in the standard normal distribution.

B. Spatial weight matrix

A crucial element of any cross-sectional study of spatial dependency is the use of spatial weights. They are a crucial component in the creation of spatial autocorrelation.

The proximity matrix (weight matrix), represented by W, is of the order n × n, made up of weights that indicate how strongly the various areal units are associated spatially. The weight matrix is expressed as

$$W = \begin{bmatrix} w_{11} & w_{12} & \cdots & w_{1n} \\ w_{21} & w_{22} & \cdots & w_{2n} \\ \vdots & \vdots & \ddots & \vdots \\ w_{n1} & w_{n2} & \cdots & w_{nn} \end{bmatrix}$$

each of entry w_{ij} contains the strength of spatial association between the units i and j, for i, j = 1,2, … , n. w_{ii} is set to zero for all i=1,2, … , n and w_{ij} is assigned either 0 or 1 where i≠ j.

$$w_{ij} = \begin{cases} 1 \text{ if i and j share a common boundary} \\ 0 \text{ otherwise} \end{cases}$$

In the context of grid-based areal data, the proximity matrix is determined by touching areal units, it is helpful to indicate whether "queen" based neighbors or "rook" based neighbors (as in a chess

game) are being utilized. While "rook" based neighbors employ the more stringent requirement that both areal units share an edge, "queen" based neighbors refer to any touching areal units. This is exactly the same as in chess. To better understand this, consider the image

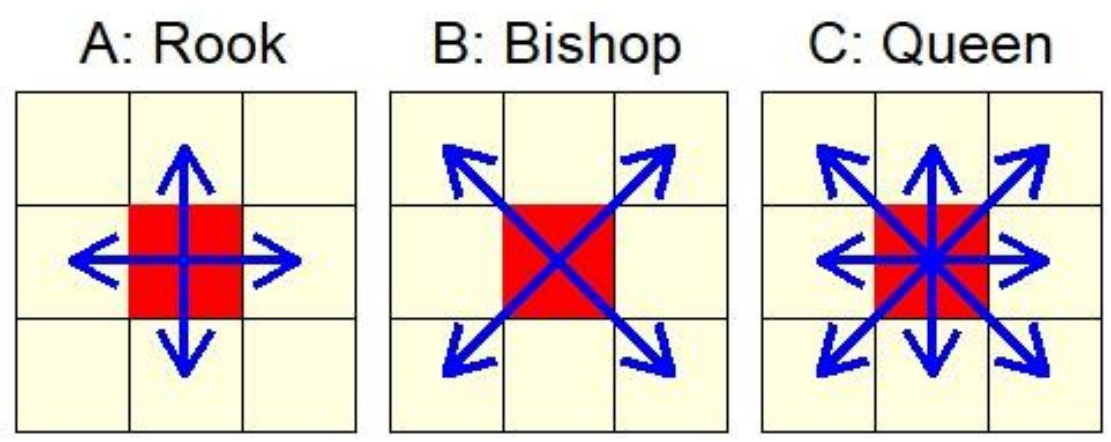

Figure 1: Spatial Weight Matrix Neighborhood criteria.

C. Test of spatial autocorrelation

The hypothesis to be tested is

H_0: Spatial autocorrelation is absent and H_1: Spatial autocorrelation is present

If the null hypothesis is rejected, indicating the presence of spatial autocorrelation we can then use Global Moran's I or Geary's C in this purpose. Now, calculate the test statistic

$$z = \frac{X - E[X]}{\sqrt{Var(X)}}$$

where,

X is the observed value of the chosen statistic (Global Moran's I or Geary's C), E(X) is the expected value under the null hypothesis of the chosen statistic, Var (X) is the variance under the null hypothesis of the chosen statistic.

By comparing the z-score to the standard normal distribution, get the p-value for the observed data. If the p-value is less than the chosen significance level, reject the null hypothesis of randomness; otherwise accept it.

- For Moran's I

  Under the null hypothesis of no spatial autocorrelation, observations Y_i are independently and identically distributed, and is asymptotically normally distributed with mean

$$E[I] = \frac{-1}{n-1}$$

and variance $\quad \mathrm{Var}[I] = \frac{n^2(n-1)S_1 - n(n-1)S_2 - 2S_0^2}{(n+1)(n-1)^2 S_0^2}$

Here, $\quad S_0 = \sum_{i \neq j} w_{ij}$,

$$S_1 = \frac{1}{2}\sum_{i \neq j}\left(w_{ij} + w_{ji}\right)^2 ,$$

$$S_2 = \sum_k\left(\sum_j w_{kj} + \sum_i w_{ik}\right) .$$

- For Geary's C

Geary C has a theoretical expected value 1 i.e. $E(C) = 1$ and the variance is

$$Var(C) = \frac{(n-1)S_1(n^2 - 3n + 3 - k(n-1))}{S_0^2\, n(n-2)(n-3)} + \frac{(n^2 - 3 - k(n-1)^2)}{n(n-2)(n-3)} - \frac{(n-1)S_2(n^2 + 3n - 6 - k(n^2 - n + 2))}{4\, S_0^2\, n(n-2)(n-3)}$$

here,

$$S_1 = \frac{1}{2}\sum_{i=1}^{n}\sum_{j=1}^{n}\left(w_{ij} + w_{ji}\right)^2,$$

$$S_2 = \sum_{i=1}^{n}\left(\sum_{i=1}^{n} w_{ij} + \sum_{j=1}^{n} w_{ij}\right)^2 ,$$

$$k = \frac{n\sum_{i=1}^{n}(Y_i - \overline{Y})^2}{(\sum_{i=1}^{n}(Y_i - \overline{Y})^2)^2}$$

D. DIC

In Bayesian statistics, the Deviance Information Criterion (DIC) is a method for choosing models that balances complexity with model fit. It evaluates model performance using the posterior

distribution, combining model fit and complexity into a single result. To be specific, it is the Bayesian version of the Akaike Information Criterion (AIC). The AIC is defined as

$$\mathrm{AIC} = 2\mathrm{k} - 2\ln\hat{\mathrm{L}}$$

where, k is the number of parameters in the model and $\ln\hat{\mathrm{L}}$ is the log of maximum likelihood.

And DIC is defined as $DIC = \overline{D} + p_D$

where, $\overline{D}$ is the posterior mean of the deviance and $\mathrm{Deviance} = -2\log(\mathrm{p}(\mathrm{y}|\hat{\theta}))$

here, y comprises all the stochastic nodes given the data and $\hat{\theta}$ comprises the immediate stochastic parents of y. In simpler words, it is the log-likelihood evaluated at bayes estimate $\hat{\theta}$. p_D is the effective number of parameters. According to Gelman, p_D is half of the variance of deviance. p_D represents a penalty term for model complexity. Models with low DIC values are preferred as they indicate both a good fit to the data (low $\overline{D}$) and a simpler model (low p_D).

E. Trace plot assessing for convergence of the Conv.ZIP(best fitted model) parameters

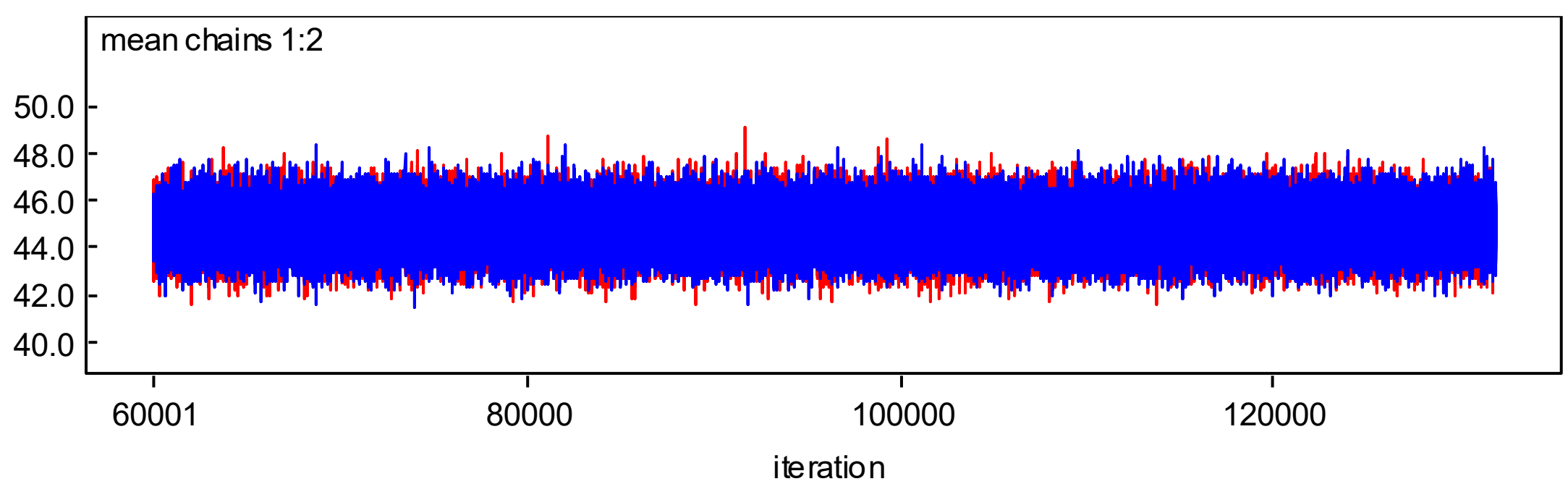

Figure 2:Trace plot of Posterior mean of malaria (due to disaster)

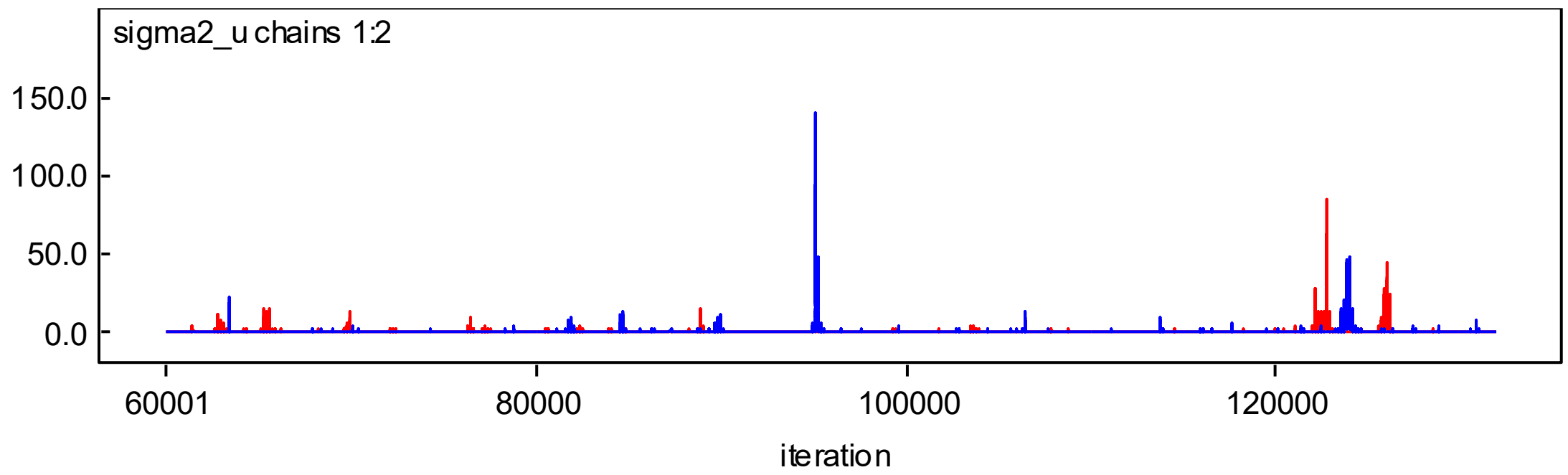

Figure 3:Trace plot of Spatial variance,$\widehat{\sigma_u^2}$

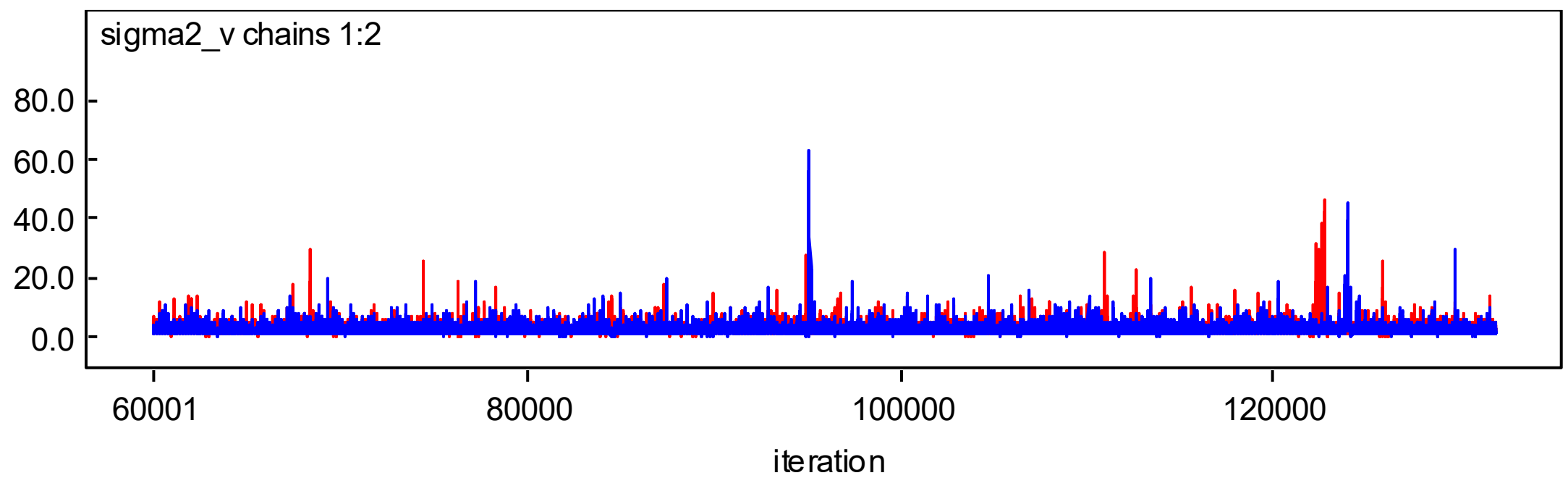

Figure 4:Trace plot of Unstructured variance,$\widehat{\sigma_v^2}$

F. Kernel Density plot of the Conv.ZIP(best fitted model) parameters

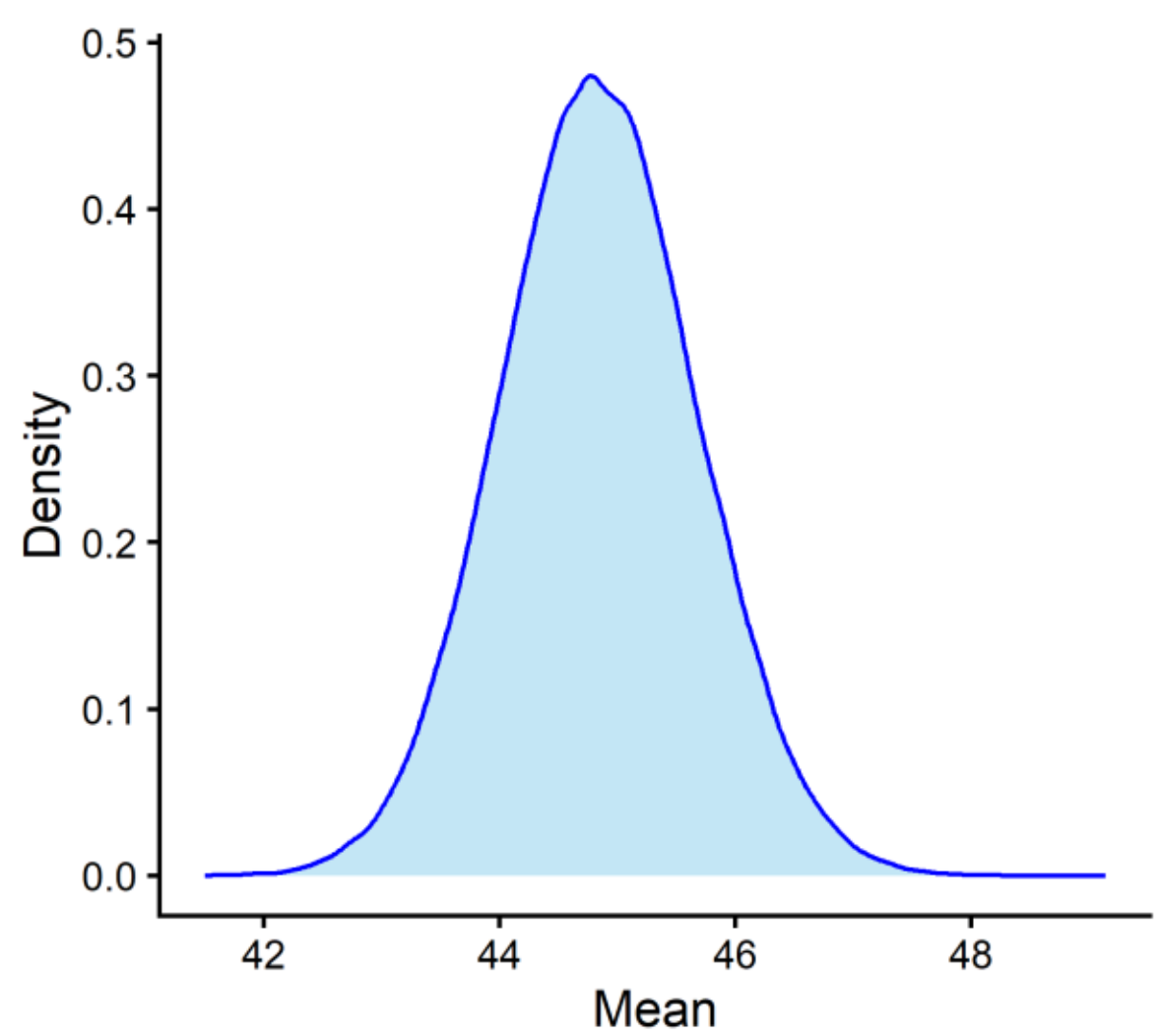

Figure 5:Kernel Density plot of Posterior mean of malaria (due to disaster)

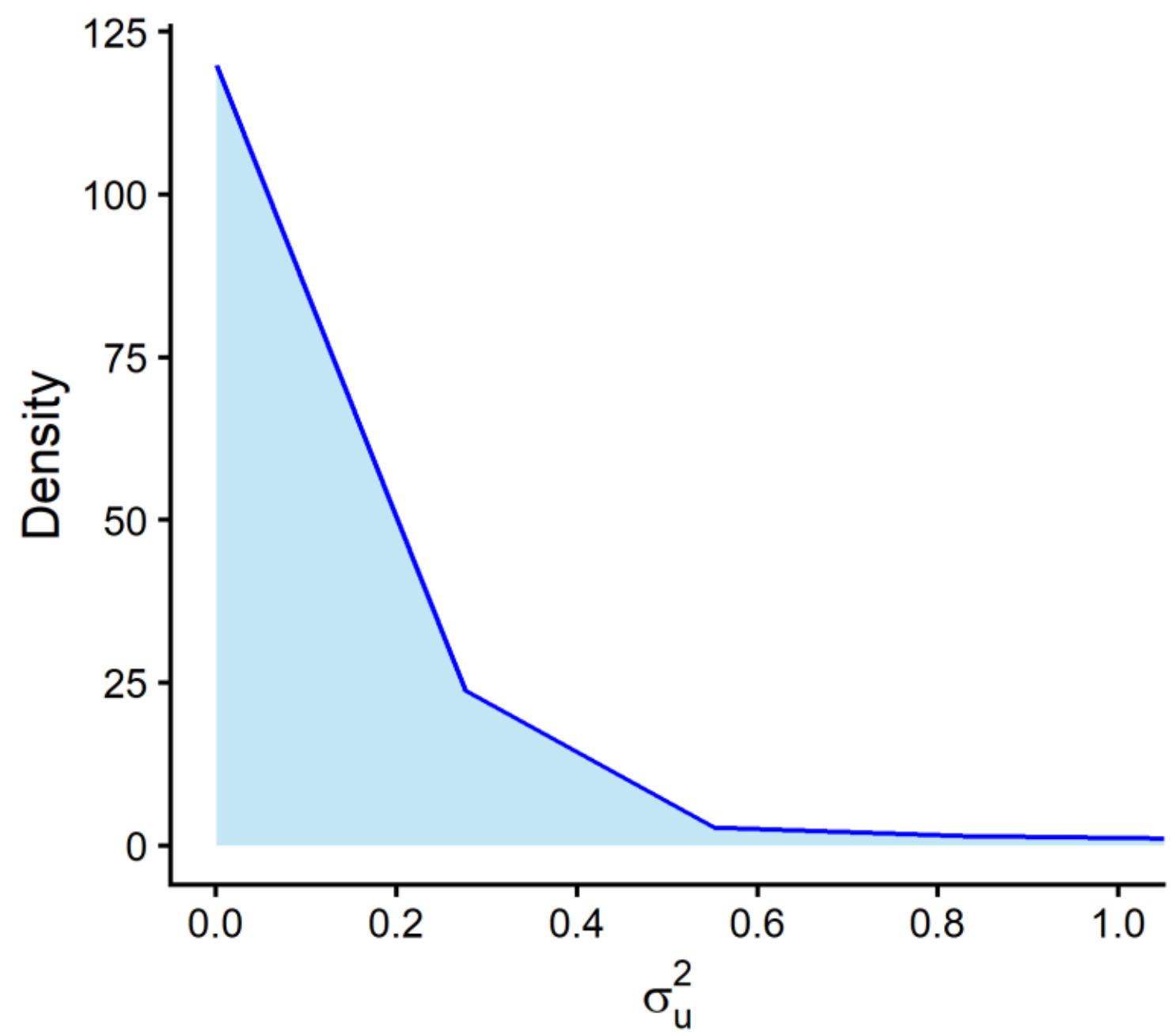

Figure 6: Kernel Density plot of Spatial variance, $\widehat{\sigma_u^2}$

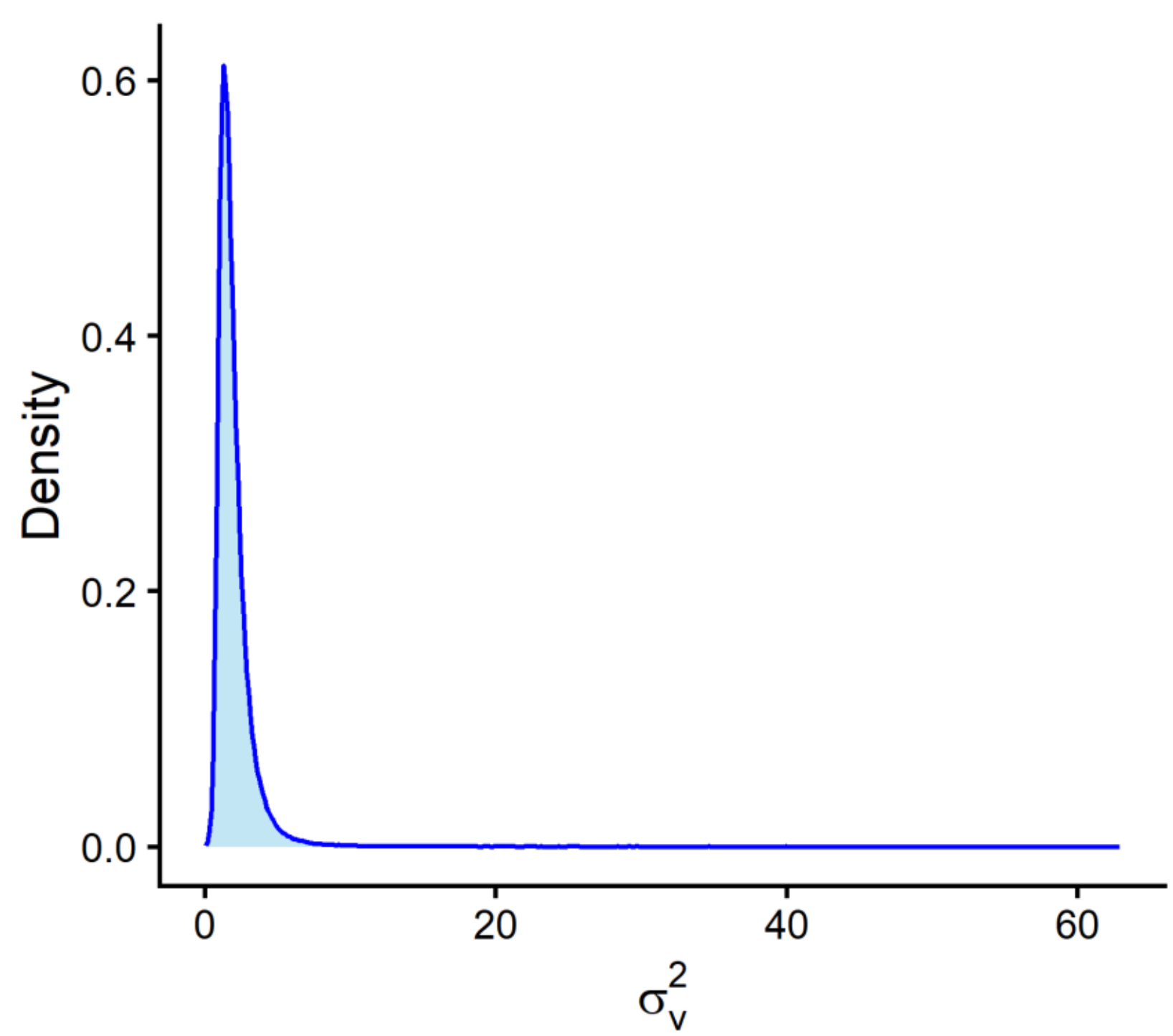

Figure 7: Kernel density plot of Unstructured variance, $\widehat{\sigma_v^2}$
