# Supplementary material for: Spatial Analysis of Malaria in Bangladesh: Insights from Bayesian Disease Mapping Models
Source: PLoS One. 2026 Aug 24;21(8):e0353483. doi: 10.1371/journal.pone.0353483 (PMC13502707; doi:10.1371/journal.pone.0353483)
Supplement: Supplementary file 2 [file pone.0353483.s002.pdf]

Spatial Analysis of Malaria in Bangladesh-insights from Bayesian Disease Mapping models

Specific comments for the authors to consider.

Abstract

Authors can point out any specific software used for analysis. The abstract does not require any sub headings. Authors can consider writing the abstract as a whole paragraph integrating each sections critically. The abstract lack clear research aim that needs to be identified.

Introduction

The introduction lacks coherent literature review. The paper also needs a conceptual/ theoretical framework for better understanding the spatial dependencies. The introduction also does not include any review on previous methods used for such kinds of spatial analysis. The research gaps identified is shallow, need better inclusion of south Asian studies for validation of the research such as

1. Barket, S. E., Karim, M. R., & Salan, M. S. A. (2025). Bayesian spatio-temporal disease mapping of COVID-19 cases in Bangladesh. *Plos one*, *20*(2), e0316621.

Authors also require to present the context of understanding the concentration of diseases for Bangladesh more critically. The write-up is little bit mixed-up and authors are recommended to polish the writing and stick to single agenda per paragraph.

Bayesian analysis improves estimates by combining prior knowledge with data, unlike the frequentist approach, which uses only the data- in this paragraph two types of issues are discussed, the benefits of the model and critical role of spatial analysis; which needs to be expanded with more review.

'Although numerous studies have examined malaria cases in Bangladesh, none have taken a comprehensive approach covering the entire country' – how is your research comprehensive, need justification?

While the article references several studies, the literature review could be expanded to include more recent research findings and theoretical frameworks that support the study's context. Consider integrating a theoretical framework that explains the relationship between public health and socio-economic factors.

Materials and Methods

The population data of each district must be taken from BBS (official statistics)- i.e. urban area report/ Census report.

In case of population growth, BBS considered exponential population growth rate during 2022 census which should be considered in your research. Assuming geometric population growth needs proper validation and scholarly references that led you to decide. How did the authors come up with the equations? Please provide numbers of the equation and cite in the text properly, as necessary.

The authors have used projected population but did not clarify the process of projection/ equation.

We need data validation process, cleaning process, attribute management process in detailed in the method section.

Clearly state the time frame you are working with in the method section. Provide a table of data sources for greater interpretability.

The method section does not include any description of the study area. People without the knowledge of Bangladesh needs to be clarified about the geographical context. Please do as appropriate.

The method section includes a number of statistical models and their application. However, the sections fundamentally aim to provide reader the process how did the authors use the formulas and tools, in detail (where needed)- which is currently missing to a broad sense. Please re-write the method section following a coherent and objective wise use of tools/statistical methods. Many of the texts is irrelevant to the paper as this article is not about the methodological contribution rather application of these methods for identification of patterns.

Include how these statistical methods are used in what extent in this section.

Provide a logical flow of methodology to understand the steps for general readers.

The method section does not clearly identify how the spatial maps are prepared and by which means?

The method section lacks coherence. Need a story of methods/ tolls used to get what. Split the method section by research aims/ objectives and then connect the aims/objectives in the end.

Data Analysis

The figures need to be sourced (the data source)

The spatial maps are not readable. Also, they are not in standard format. Include appropriate legend size, north sign, scale of the maps and increase the size of the maps. Please prepare the maps in ArcGIS/QGIS platform.

'Before the disaster (Figure 2 - upper left), the map indicated that the southern coastal such as Barguna, Bhola, and Patuakhali and southeastern hilly regions such as Bandarban, Rangamati, and Khagrachhari—along with Cox's Bazar and Chittagong had the darkest color. In contrast, several central and northern districts (exception in Rangpur and Kurigram) displayed lighter colors, reflecting lower affected'- P.13. define the disaster at first instance. General reader does not know the district names. It is required to provide a noted map in the method section or in data analysis section.

*'Therefore, we can reject the null hypothesis of no monotonic trend and conclude that there is a significant and strong decreasing monotonic trend in malaria cases over time in Bangladesh', p.11- W*here did the authors defined the null hypothesis earlier? These are crucial points that reduces the readability.

The analysis section needs restructuring and connect to the theoretical underpinning such as spatial information theory.

Be consistant with the wording such as '*Other regions like Sylhet, Dinajpur, Gaibandha, and Chittagong'*, p.14- regions and districts are different notion for Bangladesh.

The color palette ranges from light yellow to red, representing increasing rates of prevalence- p.14, this is not the way of describing the spatial maps. Please re think.

In contrast to Figure 3-left, this one provides a per capita perspective, highlighting the relative disease burden rather than merely the raw number of cases-p.15. Mark the figure with a, b, c… and cite them properly in the text such as Figure 3 (a).

The maps have may have come out of Geoda platform which is not appropriate to show case in the journal.

Line spacing is different in couple of paragraphs. Please consider checking.

DIC- explain at first instance.

Due to authorized sign-in barriers, we did not have access to the original population affected by malaria.p.16- Texts for limitation.

The English language needs a better modification perhaps. Many of the texts are hard to understand.

Figure 6: relative risk- how did the author calculated the risk? Explain in method section

Figure 7: unacceptable map (distorted scale, different projection, not in professional status)

All the figures/ maps need restructure. It is highly recommended.

Font types, sizes are different in many parts of the article which is unacceptable.

Authors have identified several aims of the research. I would suggest preparing the result section (rename data analysis section) following the research aims/objectives.

Discussion and Conclusion

The research lacks comprehensive discussion in relation with the other researches conducted before, similarly. At current stat, it is more like describing the method and results again. We need critical data insights from the results. Authors also did not consider the public health issues/policies to match up the research findings. The policy dimension, governance structure would have provided critical insights to the analysis. the conclusion must include a compiling statement of the research.

Expand the discussion on the implications of the findings for policymakers and community leaders.

Clarify the study limitations properly and way forward. Organize the conclusion by research aims/objectives. I strongly suggest adding a paragraph to re-praise your claims and synthesize it with the theories.

In short, full articles needs a cleaver restructuring in presenting idea with clarifications and visualizations. Overall, the research has some potentials but need streamlining. Good luck!
