# Supplementary material for: Spatial Analysis of Malaria in Bangladesh: Insights from Bayesian Disease Mapping Models
Source: PLoS One. 2026 Aug 24;21(8):e0353483. doi: 10.1371/journal.pone.0353483 (PMC13502707; doi:10.1371/journal.pone.0353483)
Supplement: Supplementary file 3 [file pone.0353483.s003.pdf]

Reviewer Comments

1. Insufficient focus of the literature review on the core scientific questions

The literature review part does not sufficiently addressed the key scientific concerns of this study. The malaria disease background and transmission mechanisms largely resembles a general background review or conceptual synthesis, rather than a problem-oriented critical review. This research must explicitly examine previous studies concerning the association between the disease and spatial clustering characteristics, spatial hazards, and spatial heterogeneity, while elucidating how to derive an innovative perspective from the established difficulties. Summarize and elucidate the research methodologies, data, and models from a literary criticism standpoint to demonstrate the necessity and significance of this study.

2. Overemphasis on public health and policy background in the Introduction

The literature review in introduction section should not significantly dwell on the public health and policy context of malaria.The Introduction would benefit from a more concise background section and a stronger emphasis on methodological and conceptual gaps in existing spatial malaria research.

3. Lack of a clear and comprehensive research route

Kindly supplement the comprehensive research roadmap, distinctly outlining the sequential advancement of research methodologies until the objectives are attained.

4. Conceptual and methodological concerns regarding the use of disaster-related data

Kindly request the author to ponder the subsequent scientific inquiries:

- Are there any evident limitations in utilizing disaster-related health statistics as a proxy variable for malaria monitoring data?
- Malaria is a chronic endemic disease, but catastrophe data illustrates health implications within discrete event contexts and is influenced by factors such as reporting systems, population migration, and medical disruptions. Although catastrophes may indirectly influence malaria transmission, can the malaria cases reported in disaster statistics be comparable to the typical malaria prevalence levels observed in research?

Consequently, the author must consider: Does the spatial pattern observed in this study correspond to the distribution of catastrophe exposure, and is it a general depiction of malaria risk? So, the author must explicitly delineate the research subject and meticulously analyze the findings.

5. Structural overlap between Data Analysis and Results sections

"Data Analysis" and "Data Results" must remain separated. The roles of Methodology, Analytical procedures, and Result interpretations parts have consistently overlapped and been mixed. This unclear writing is now causing a structural flaw in the scientific study.

6. Recommended restructuring of the manuscript

Kindly consider the writing structure can conducted by the sequence of Data gathering, Methodology , Data analysis, and Results presentation.

7. Avoid pre-explaining results in the Methods and data analysis section

Interpretative statements and explanations of data(model)results should be reserved for the Results and Discussion sections

8. Clarification of the role of the Discussion section

The discussion section does not merely repeat the existing results, but rather elaborates on how the research hypotheses were verified, how reasonable conclusions were derived, and why your analytical model constitutes the optimal combination for disease research in the Bangladeshi region. It also points out the research's value in terms of its reproducibility and reusability for the academic community.
