# Supplementary material for: Spatial Analysis of Malaria in Bangladesh: Insights from Bayesian Disease Mapping Models
Source: PLoS One. 2026 Aug 24;21(8):e0353483. doi: 10.1371/journal.pone.0353483 (PMC13502707; doi:10.1371/journal.pone.0353483)
Supplement: Supplementary file 6 [file pone.0353483.s004.pdf]

Reviewer Response Assessment

Manuscript Title:

Spatial Analysis of Malaria in Bangladesh: Insights from Bayesian Disease Mapping Models

Manuscript ID: PONE-D-25-54031 (Revision 1)

The authors have undertaken a substantial revision of the manuscript in response to the concerns raised. They have added new analyses, extended MCMC iterations substantially, and provided formal convergence diagnostics. However, some of my previous concerns remain incompletely addressed or reveal new issues that require further attention.

Below is a point-by-point evaluation of the authors' responses to my original comments.

Comment 1: Introduction Style and Literature Review

Assessment: Partially addressed. The Introduction has been improved and now reads more professionally.

However, the literature review still relies heavily on the same set of regional studies cited in the original version. The authors have not added meaningful engagement with broader spatial epidemiology literature beyond Bangladesh.

Comment 2: Outcome Variable Justification

Assessment: Partially addressed. Two issues remain:

(i) The authors do not specify whether "malaria due to disaster" cases are laboratory-confirmed, clinically diagnosed, or self-reported.

(ii) The Methods section (Page 4) states the data cover "between 2015 and 2020" but the analysis section (Page 16 onward) uses a single aggregated measure and this aggregation over time is not captured in the Methods section.

Furthermore, the temporal alignment between the disaster-attributed cases (2015-2020) and the rainfall covariate (2017-2020) is unclear.

Comment 3: Prior Distributions

Assessment: Addressed. The prior specifications are now reported.

Comment 4: Trace Plots and Convergence

Assessment: Addressed. Trace plots for the final model and convergence metrics included.

Comment 5: MCMC Details

Assessment: Addressed. The MCMC details are now provided.

Comment 6: Numerical Convergence Diagnostics

Assessment: Partially addressed. For the preferred Conv._ZIP model, the effective sample sizes are adequate (e.g., 140,000 for the mean). However, the Poisson-Lognormal and Conv. models have very low effective sample sizes (as low as 89 and 183), yet they remain in Table 3. The inclusion of models with clear convergence problems in Table 3 is problematic.

Comment 7: Model Comparison: DIC and Parameter Estimates

Assessment: Inadequately addressed. The presence of multiple poorly fitting models in Table 3 undermines confidence in the model comparison framework. Extremely large DIC values and implausible variance estimates persist.

Recommendation: Either add the following footnote to Table 3:

> *The CAR, Convolution, and CAR_ZIP models exhibited substantially higher DIC values and wider credible intervals compared to the Conv._ZIP model, indicating poor fit for the sparse zero-inflated malaria data. These models are included for completeness only and are not recommended for inference. All conclusions are based on the Conv._ZIP model (without rainfall covariate).*

Or remove the poorly performing models from Table 3 entirely, presenting only: Poisson-Gamma, Poisson-Lognormal (non-spatial baselines), and Conv._ZIP (best fit).

Comment 8: Kernel Density Plots

Assessment: Acceptable, but with concerns. The authors removed the kernel density plots entirely from the revised Appendix, which means readers cannot evaluate posterior distributions for themselves. Consider adding kernel density plots for the final model to the Appendix.

Comment 9: Figure Quality and Relative Risk Values

Assessment: Satisfactory The maps look better now, although it is still a bit difficult to fully assess the resolution from the files provided. To get this issue resolved, authors may write `R codes` to save the images directly.
The explanation for small RR values is technically correct. Also, the authors have now removed Table 3.5 (low-risk areas) entirely, which was a reasonable decision given the implausible values.

Comment 10: Covariate Construction

Assessment: Addressed. This clarification is now included in the manuscript.

Comment 11: Repeated Equations

Assessment: Addressed. The equations are well structured.

Comment 12: ZIP Model Definition and Zero Inflation Evidence

Assessment: Addressed but new issues raised. The ZIP model structure is now defined, and evidence of zero inflation and overdispersion is presented. However, the results for the zero-inflation component (e.g., the estimated probability π of an excess zero) are still not reported in Tables 3 or 4. The authors mention a Uniform(0,1) prior for zero-inflation probability in Section 2.18, but the posterior estimate is never presented or discussed.

Comment 13: Overinterpretation of Malaria Elimination

Assessment: Partially addressed. The Discussion and Conclusion have been substantially rewritten with more cautious language. However, the Abstract still states "Bangladesh is making significant progress toward malaria elimination" and "many districts already achieving zero cases" based on disaster-attributed data, which remains an overstatement. Revise the Abstract to reflect disaster-attributed data limitations.

Comment 14: Missing Access Dates

Assessment: Addressed. The references now include access dates.

Comment 15: Additional issues raised regarding Table 3 and 4

(i) The way the $\hat{R}$ and n_{eff} values are reported in parentheses in Tables 3 and 4 is confusing. Consider reporting the Gelman-Rubin values to two decimal places (e.g., 1.02) leaving a space after the comma for clarity. For example, write "(1.02, 140000)" instead of "(1,140000)".

(ii) The caption of Table 3 defines τ_u^2 as "the variance of unstructured heterogeneity" but the corresponding row label reads "Spatial precision". This is a contradiction. Additionally, Table 4 includes τ_u^2 in the caption, but no column named τ_u^2 appears in the table. Both captions must be corrected for consistency.

(iii) For the Conv._ZIP model, $\sigma_v^2 = 1.957$ and $\tau_v^2 = 0.661$. If $\tau^2 = 1/\sigma^2$, then $1/1.957 = 0.511$, not 0.661. Similarly, $\sigma_u^2 = 0.229$ and $\tau_u^2 = 89.540$, but $1/0.229 = 4.367$; therefore, we would expect the value to be approximately 4.367, not 89.540. The authors should clarify whether τ^2 is defined as $1/\sigma^2$ or estimated as a separate parameter.

(iv) The footnote of Table 3 states that bold values denote "parameters of significant importance" but no statistical criterion is provided to define what constitutes "significant importance".

In conclusion, the authors have made a substantial effort to address the methodological concerns, particularly by extending MCMC runs, adding convergence diagnostics, and expanding the spatial analyses. However, critical issues remain unaddressed. With these additional revisions, the manuscript could be suitable for publication.
