# Supplementary material for: Spatial Analysis of Malaria in Bangladesh: Insights from Bayesian Disease Mapping Models
Source: PLoS One. 2026 Aug 24;21(8):e0353483. doi: 10.1371/journal.pone.0353483 (PMC13502707; doi:10.1371/journal.pone.0353483)
Supplement: Supplementary file 7 [file pone.0353483.s006.pdf]

Reviewer Response Assessment

Manuscript Title:

Spatial Analysis of Malaria in Bangladesh: Insights from Bayesian Disease Mapping Models

Manuscript ID: PONE-D-25-54031 (Revision 2)

The authors have carefully addressed all of the concerns raised in the previous round of review. Below is a point by point evaluation.

Comment 1: Introduction Style and Literature Review

Assessment: Fully addressed. The authors added references to spatial epidemiology studies from India, Nepal, and sub-Saharan Africa.

Comment 2: Outcome Variable Justification

Assessment: Fully addressed.

(i) The manuscript now explicitly states that the BDRS data are administratively reported and do not distinguish between laboratory-confirmed, clinically diagnosed, or self-reported cases. This limitation is clearly noted.

(ii) The Methods section now clarifies that the malaria data represent a single cumulative statistic for 2015–2020. The temporal mismatch with rainfall (2017–2020) is acknowledged and justified as a cross-sectional aggregated analysis.

Comment 3: Prior Distributions

Assessment: Fully addressed. Prior specifications are reported in Section 2.18.

Comment 4: Trace Plots and Convergence

Assessment: Fully addressed. Trace plots and convergence metrics for the final model are included.

Comment 5: MCMC Details

Assessment: Fully addressed. MCMC settings are clearly reported.

Comment 6: Numerical Convergence Diagnostics

Assessment: Fully addressed. The authors retained poorly converged models in Table 3 but added a clear footnote stating that these models are included only for comparative evaluation and are not recommended for inference.

Comment 7: Model Comparison: DIC and Parameter Estimates

Assessment: Fully addressed. The same footnote statement addresses this concern.

Comment 8: Kernel Density Plots

Assessment: Fully addressed. Kernel density plots for the final model have been added to the Appendix.

Comment 9: Figure Quality and Relative Risk Values

Assessment: Fully addressed. Figures are now exported at 300 dpi from R. The explanation for small RR values is satisfactory and the low-risk table has been removed.

Comment 10: Covariate Construction

Assessment: Fully addressed. Rainfall construction is clearly described.

Comment 11: Repeated Equations

Assessment: Fully addressed. Equations are now well structured and numbered.

Comment 12: ZIP Model Definition and Zero Inflation Evidence

Assessment: Fully addressed. The posterior estimate of the zero-inflation probability ($\hat{\pi} = 0.817$) is now reported in the Results section and its interpretation is provided.

Comment 13: Overinterpretation of Malaria Elimination

Assessment: Fully addressed. The Abstract has been revised to reflect that the findings represent relative risk patterns based on disaster-related data hence the issue of overstatement has been corrected.

Comment 14: Missing Access Dates

Assessment: Fully addressed.

Comment 15: Additional issues regarding Tables 3 and 4

Assessment: Fully addressed.

(i) Formatting of $\hat{R}$ and n_{eff} values has been corrected to two decimal places with clear spacing.

(ii) Table captions have been revised for consistency; contradictions removed.

(iii) The authors clarified that variance is computed as $E[1/\tau]$ rather than $1/E[\tau]$ which explains the non-reciprocal values. This is a statistically sound explanation and is now stated in Section 2.18.

(iv) The footnote has been revised to indicate that bold formatting is for readability hence the concern is addresed.

Overall Recommendation

Accept. The authors have thoroughly addressed all concerns raised earlier. The manuscript in my opinion, is now methodologically sound, transparent about its limitations and clearly written.
