# Supplementary material for: Spatial Analysis of Malaria in Bangladesh: Insights from Bayesian Disease Mapping Models
Source: PLoS One. 2026 Aug 24;21(8):e0353483. doi: 10.1371/journal.pone.0353483 (PMC13502707; doi:10.1371/journal.pone.0353483)
Supplement: Supplementary file 4 [file pone.0353483.s005.docx]

**Response to Reviewers**

Manuscript Title: Spatial Analysis of Malaria in Bangladesh: Insights from Bayesian Disease Mapping Models

Manuscript ID: PONE-D-25-54031 (Revision 1)

Journal: PLOS ONE

We sincerely thank the reviewer for the careful evaluation of our revised manuscript and for the constructive comments provided. We appreciate the reviewer’s insightful suggestions, which have helped us further improve the clarity, rigor, and transparency of our work. Below, we address each point in detail.

# **Comment #1: Introduction Style and Literature Review**

**Comment:** The Introduction has been improved and now reads more professionally. However, the literature review still relies heavily on the same set of regional studies cited in the original version. The authors have not added meaningful engagement with broader spatial epidemiology literature beyond Bangladesh.

**Response:** We have revised the Introduction to incorporate broader spatial epidemiology literature beyond Bangladesh. Specifically, we have added references to studies from India, Nepal, and sub-Saharan Africa that apply spatial statistical methods to malaria and demonstrate spatial clustering, ecological heterogeneity, and hotspot persistence.

# **Comment #2: Outcome Variable Justification**

**Comment 2(i):** The authors do not specify whether “malaria due to disaster” cases are laboratory confirmed, clinically diagnosed, or self-reported.

**Response:** The “malaria due to disaster” data was collected from Bangladesh Disaster-related Statistics 2021(BDRS-2021) report published by the Bangladesh Bureau of Statistics (BBS). This is administratively reported cases for the period 2015–2020 and does not distinguish specifically between laboratory-confirmed, clinically diagnosed, or self-reported cases.

**Comment 2(ii):** The Methods section (Page 4) states the data cover “between 2015 and 2020” but the analysis section (Page 16 onward) uses a single aggregated measure and this aggregation over time is not captured in the Methods section.

Furthermore, the temporal alignment between the disaster-attributed cases (2015-2020) and the rainfall covariate (2017-2020) is unclear.

**Response:** The malaria dataset from the BDRS-2021 report represents a single district-level cumulative statistic aggregated over the period 2015–2020. This clarification has now been explicitly added to the Methods (Data source) like this “Importantly, the reported values represent a single cumulative (aggregated) statistic for the entire period 2015–2020. Therefore, each district is represented by one value reflecting the total burden over the period.” to improve transparency regarding temporal aggregation.

For rainfall, we use district-level annual rainfall data for 2017–2020, as data for 2015–2016 are not available, and construct a cumulative rainfall measure over this period. This is done to ensure consistency with the aggregated structure of the malaria outcome variable. Since the malaria data is a fully aggregated outcome over 2015–2020 and the analysis is conducted at the district level as a cross-sectional framework, the rainfall measure is used as an aggregated environmental exposure over the available and most consistent period, and this temporal limitation does not affect the validity of the model structure.

# **Comment #6: Numerical Convergence Diagnostics**

**Comment:** For the preferred Conv. ZIP model, the effective sample sizes are adequate (e.g., 140,000 for the mean). However, the Poisson-Lognormal and Conv. models have very low effective sample sizes (as low as 89 and 183), yet they remain in Table 3. The inclusion of models with clear convergence problems in Table 3 is problematic.

**Response:** The CAR, Convolution, and CAR ZIP models are retained in Table 3 to provide a comprehensive comparison of alternative spatial and zero-inflated specifications, allowing assessment of model performance under different assumptions. To avoid any ambiguity, we have added a table footnote recommended by the reviewer.

# **Comment #7: Model Comparison: DIC and Parameter Estimates**

**Comment:** The presence of multiple poorly fitting models in Table 3 undermines confidence in the model comparison framework. Extremely large DIC values and implausible variance estimates persist. Either add the recommended footnote to Table 3.

**Response:** We have added the recommended footnote to Table 3.

# **Comment #8: Kernel Density Plots**

**Comment:** The authors removed the kernel density plots entirely from the revised Appendix, which means readers cannot evaluate posterior distributions for themselves. Consider adding kernel density plots for the final model to the Appendix.

**Response:** Kernel density plots of the final model have been added to the Appendix to allow readers to assess posterior distributions.

# **Comment #9: Figure Quality and Relative Risk Values**

**Comment:** The maps look better now, although it is still a bit difficult to fully assess the resolution from the files provided. To get this issue resolved, authors may write R codes to save the images directly.

**Response:** Thank you for the suggestion. All figures in the revised manuscript have been generated and exported directly from R in TIFF format at 300 dpi resolution, consistent with journal guidelines. We believe the current figure quality is appropriate for publication.

# **Comment #12: ZIP Model Definition and Zero Inflation Evidence**

**Comment:** The ZIP model structure is now defined, and evidence of zero inflation and overdispersion is presented. However, the results for the zero-inflation component (e.g., the estimated probability *π* of an excess zero) are still not reported in Tables 3 or 4. The authors mention a Uniform (0,1) prior for zero-inflation probability in Section 2.18, but the posterior estimate is never presented or discussed.

**Response:** The posterior estimate of the zero-inflation probability (π) has now been reported and discussed in the Results section, demonstrating the presence of excess zeros in the data.

# **Comment #13: Overinterpretation of Malaria Elimination**

**Comment:** The Discussion and Conclusion have been substantially rewritten with more cautious language. However, the Abstract still states “Bangladesh is making significant progress toward malaria elimination” and “many districts already achieving zero cases” based on disaster-attributed data, which remains an overstatement. Revise the Abstract to reflect disaster-attributed data limitations.

**Response:** The Abstract has been revised to avoid overstatement and to more accurately reflect the nature of the data used in this study. Specifically, we clarified that the district-level malaria variable represents disaster-associated aggregated counts and is used as a proxy indicator of relative malaria vulnerability rather than a direct measure of malaria transmission. Also, the revised Abstract now explicitly states that the findings should be interpreted as relative spatial risk patterns under data limitations.

# **Comment #15: Additional issues raised regarding Table 3 and 4**

**Comment 15(i):** The way the $\hat{R}$ and *n*_eff_ values are reported in parentheses in Tables 3 and 4 is confusing.
Consider reporting the Gelman-Rubin values to two decimal places (e.g., 1.02) leaving a space after the comma for clarity. For example, write “(1.02, 140000)” instead of “(1,140000)”.

**Response:** The formatting has been corrected to improve clarity, with appropriate spacing and decimal representation.

**Comment 15(ii):** The caption of Table 3 defines *τ_u_*^2^ as “the variance of unstructured heterogeneity” but the corresponding row label reads “Spatial precision”. This is a contradiction. Additionally, Table 4 includes *τ_u_*^2^ in the caption, but no column named *τ_u_*^2^ appears in the table. Both captions must be corrected for consistency.

**Response:** Thank you for this helpful suggestion. We have revised the captions of Tables 3 and 4. The revised captions ensure that all notation is clearly defined and consistently used across tables, addressing the reviewer’s concern.

**Comment 15(iii):** For the Conv. ZIP model, *σ_v_*^2^ = 1*.*957 and *τ_v_*^2^ = 0*.*661. If *τ*^2^ = 1*/σ*^2^, then 1*/*1*.*957 = 0*.*511, not 0*.*661. Similarly, *σ_u_*^2^ = 0*.*229 and *τ_u_*^2^ = 89*.*540, but 1*/*0*.*229 = 4*.*367; therefore, we would expect the value to be approximately 4*.*367, not 89*.*540. The authors should clarify whether *τ*^2^ is defined as 1*/σ*^2^ or estimated as a separate parameter.

**Response:** Thank you for this important observation. In our model, variance is obtained at each MCMC iteration as $\sigma^{2}=1/\tau$, and the reported posterior mean corresponds to the average of these transformed values, i.e., $E[1/\tau]$. The precision parameter is summarized separately as $E[\tau]$. Since the averaging is performed after transformation, the reported variance corresponds to mean(1/τ), not 1/mean(τ). Therefore, the two quantities are not exact reciprocals, which explains the observed difference. Also, we have now clarified in the manuscript that variance parameters are defined as the inverse of precision and are computed at each MCMC iteration.

**Comment 15(iv):** The footnote of Table 3 states that bold values denote “parameters of significant importance” but no statistical criterion is provided to define what constitutes “significant importance”.

**Response:** We agree that the previous wording was unclear and could be misinterpreted. We have revised the table footnote to specify that bold formatting is applied to the posterior mean, variance components, 95% credible interval of posterior mean and DIC solely for readability.
