# Supplementary material for: Spatial Analysis of Malaria in Bangladesh: Insights from Bayesian Disease Mapping Models
Source: PLoS One. 2026 Aug 24;21(8):e0353483. doi: 10.1371/journal.pone.0353483 (PMC13502707; doi:10.1371/journal.pone.0353483)
Supplement: Peer review history

# Peer review: Decision Letter 0

**DOI:** 10.1371/journal.pone.0353483.r001

**Authors:** Denekew Bitew Belay

26 Dec 2025

Dear Dr.  Karim,

Thank you for submitting your manuscript to PLOS ONE. After careful consideration, we feel that it has merit but does not fully meet PLOS ONE’s publication criteria as it currently stands. Therefore, we invite you to submit a revised version of the manuscript that addresses the points raised during the review process.

Please submit your revised manuscript by Feb 09 2026 11:59PM. If you will need more time than this to complete your revisions, please reply to this message or contact the journal office at plosone@plos.org. When you're ready to submit your revision, log on to https://www.editorialmanager.com/pone/ and select the 'Submissions Needing Revision' folder to locate your manuscript file.

- A letter that responds to each point raised by the academic editor and reviewer(s). You should upload this letter as a separate file labeled 'Response to Reviewers'.
- A marked-up copy of your manuscript that highlights changes made to the original version. You should upload this as a separate file labeled 'Revised Manuscript with Track Changes'.
- An unmarked version of your revised paper without tracked changes. You should upload this as a separate file labeled 'Manuscript'.

If you would like to make changes to your financial disclosure, please include your updated statement in your cover letter. Guidelines for resubmitting your figure files are available below the reviewer comments at the end of this letter.

If applicable, we recommend that you deposit your laboratory protocols in protocols.io to enhance the reproducibility of your results. Protocols.io assigns your protocol its own identifier (DOI) so that it can be cited independently in the future. For instructions see: https://journals.plos.org/plosone/s/submission-guidelines#loc-laboratory-protocols. Additionally, PLOS ONE offers an option for publishing peer-reviewed Lab Protocol articles, which describe protocols hosted on protocols.io. Read more information on sharing protocols at https://plos.org/protocols?utm_medium=editorial-email&utm_source=authorletters&utm_campaign=protocols.

We look forward to receiving your revised manuscript.

Kind regards,

Denekew Bitew Belay, Ph.D

Academic Editor

PLOS One

Journal Requirements:

If the reviewer comments include a recommendation to cite specific previously published works, please review and evaluate these publications to determine whether they are relevant and should be cited. There is no requirement to cite these works unless the editor has indicated otherwise.

Additional Editor Comments:

When submitting your revision, we need you to address these additional requirements.

1.Please ensure that your manuscript meets PLOS ONE's style requirements, including those for file naming. The PLOS ONE style templates can be found at

https://journals.plos.org/plosone/s/file?id=wjVg/PLOSOne_formatting_sample_main_body.pdf and

https://journals.plos.org/plosone/s/file?id=ba62/PLOSOne_formatting_sample_title_authors_affiliations.pdf

2. Please note that your Data Availability Statement is currently missing the repository name and/or the DOI/accession number of each dataset OR a direct link to access each database. If your manuscript is accepted for publication, you will be asked to provide these details on a very short timeline. We therefore suggest that you provide this information now, though we will not hold up the peer review process if you are unable.

3. Please ensure to list all authors and all affiliations as per our author instructions and clearly indicate the corresponding author.

4. We note that Figures 2-8 in your submission contain map images which may be copyrighted. All PLOS content is published under the Creative Commons Attribution License (CC BY 4.0), which means that the manuscript, images, and Supporting Information files will be freely available online, and any third party is permitted to access, download, copy, distribute, and use these materials in any way, even commercially, with proper attribution. For these reasons, we cannot publish previously copyrighted maps or satellite images created using proprietary data, such as Google software (Google Maps, Street View, and Earth). For more information, see our copyright guidelines: http://journals.plos.org/plosone/s/licenses-and-copyright.

We require you to either (1) present written permission from the copyright holder to publish these figures specifically under the CC BY 4.0 license, or (2) remove the figures from your submission:

1. You may seek permission from the original copyright holder of Figures 2-8 to publish the content specifically under the CC BY 4.0 license.

We recommend that you contact the original copyright holder with the Content Permission Form (http://journals.plos.org/plosone/s/file?id=7c09/content-permission-form.pdf) and the following text:

“I request permission for the open-access journal PLOS ONE to publish XXX under the Creative Commons Attribution License (CCAL) CC BY 4.0 (http://creativecommons.org/licenses/by/4.0/). Please be aware that this license allows unrestricted use and distribution, even commercially, by third parties. Please reply and provide explicit written permission to publish XXX under a CC BY license and complete the attached form.”

Please upload the completed Content Permission Form or other proof of granted permissions as an "Other" file with your submission.

In the figure caption of the copyrighted figure, please include the following text: “Reprinted from [ref] under a CC BY license, with permission from [name of publisher], original copyright [original copyright year].”

2. If you are unable to obtain permission from the original copyright holder to publish these figures under the CC BY 4.0 license or if the copyright holder’s requirements are incompatible with the CC BY 4.0 license, please either i) remove the figure or ii) supply a replacement figure that complies with the CC BY 4.0 license. Please check copyright information on all replacement figures and update the figure caption with source information. If applicable, please specify in the figure caption text when a figure is similar but not identical to the original image and is therefore for illustrative purposes only.

The following resources for replacing copyrighted map figures may be helpful:

USGS National Map Viewer (public domain): http://viewer.nationalmap.gov/viewer/

The Gateway to Astronaut Photography of Earth (public domain): http://eol.jsc.nasa.gov/sseop/clickmap/

Maps at the CIA (public domain): https://www.cia.gov/library/publications/the-world-factbook/index.html and https://www.cia.gov/library/publications/cia-maps-publications/index.html

NASA Earth Observatory (public domain): http://earthobservatory.nasa.gov/

Landsat: http://landsat.visibleearth.nasa.gov/

USGS EROS (Earth Resources Observatory and Science (EROS) Center) (public domain): http://eros.usgs.gov/#

Natural Earth (public domain): http://www.naturalearthdata.com/

5. Please ensure that you refer to Figure 1,5 and 6 in your text as, if accepted, production will need this reference to link the reader to the figure.

6. We note you have included a table to which you do not refer in the text of your manuscript. Please ensure that you refer to Table 3.1 and Table 3.2 in your text; if accepted, production will need this reference to link the reader to the Table.

[Note: HTML markup is below. Please do not edit.]

Reviewers' comments:

Reviewer's Responses to Questions

**Comments to the Author**

1. Is the manuscript technically sound, and do the data support the conclusions?

Reviewer #1: Yes

Reviewer #2: Yes

Reviewer #3: Partly

Reviewer #4: Yes

Reviewer #5: No

Reviewer #6: No

**********

2. Has the statistical analysis been performed appropriately and rigorously?

Reviewer #1: Yes

Reviewer #2: Yes

Reviewer #3: I Don't Know

Reviewer #4: Yes

Reviewer #5: Yes

Reviewer #6: No

**********

3. Have the authors made all data underlying the findings in their manuscript fully available?

The PLOS Data policy

Reviewer #1: Yes

Reviewer #2: Yes

Reviewer #3: Yes

Reviewer #4: No

Reviewer #5: Yes

Reviewer #6: No

**********

4. Is the manuscript presented in an intelligible fashion and written in standard English?

Reviewer #1: Yes

Reviewer #2: Yes

Reviewer #3: Yes

Reviewer #4: Yes

Reviewer #5: No

Reviewer #6: Yes

**********

Reviewer #1: General Comments

The manuscript describes a Bayesian spatial model for data related to malarial infections in Bangladesh. The manuscript has a lot of relevance in the domain of public healthcare, but unfortunately, it needs a lot of improvement and modifications in terms of data, methodology, interpretation of results, and manuscript composition.

Specific Comments

1. Title and Abstract

- Include a colon in the title after "Bangladesh": "Spatial Analysis of Malaria in Bangladesh: Insights from Bayesian Disease Mapping Models".

- There appears to be a discrepancy between a citation for a report from "Bangladesh Bureau of Statistics" in the abstract, and other, mostly news article citations in methods. This major discrepancy needs to be taken care of.

- The abstract refers to using rain as a covariate, but it does not report that it was a significant predictor, for example. Summarize this result.

2. Introduction

- Instead of starting with a story ("There is a saying that 'to break a butterfly upon a wheel'."), introduce a direct explanation of how much malaria affects the public health of Bangladesh and the goal of elimination.

- The literature review would benefit from being more specific concerning the knowledge gap: there has been a lack of recent, country-wide spatial analyses using data which covers all malaria parasite species, not just P. falciparum.

- The last paragraph needs to identify specifically the objectives of the study.

3. Methods

Data Sources:

- Major Issue: The mention of sources for cases of malaria is not acceptable in a research article. Relying on “the leading newspaper, the Daily Sun” and a published paper for a secondary analysisin[11] is not trustworthy. The sources you use should be official data from either the National Malaria Elimination Program (NMEP) or the Directorate General of Health Services (DGHS). If this becomes genuinely impossible, this would become a major limitation.

- Explain, with justification, why “malaria (due to disaster)” data in the BBS 2021 report is used as a proxy for total malaria spatial burden. This represents a major limitation.

- To project population, you would identify the years when data for initial population size (`P_0`) and final population size (`P_n`) are needed for calculation of geometric rate of growth.

Statistical Methods:

- In Section 2.11, justify how the adjacency matrix (`w_ij`) was defined for CAR and CONVOLUTION models in terms of queen contiguity or distance-based definition.

While introducing models like Poisson Gamma, CAR models, etc., it is important to mention briefly for what purpose a particular model is used.

- Ethics Statement: While the statement on page 31 seems fine, it would be appropriate to change “N/A” in the submission form entry on page 3 to reflect that it involves publicly available, aggregated statistical data.

4. Results

- Figure 1: The description of this graph needs to mention what data points are being shown.

- Figures 2, 3, 4, 6, 7, 8: Make sure that all maps contain a scale bar, a north arrow, and legible legends. The colors used in the LISA map, in Figure 4, should be defined in the caption.

I think the figures can be improved and present in best way.

- Spatial Autocorrelation: While discussing Moran's I of 0.211, avoid using "small." Instead, say it "indicates positive spatial autocorrelation, which is statistically significant with a p-value of <0.001."

- Table 3.3: There are no descriptive titles for the column headers. Use descriptive names. Symbol definitions are needed in the caption for this and all other tables. Examples are σ_u^2, τ_u^2, p_D.

- Rainfall Covariate: There is no interpretation of results for the rainfall covariate. To report in the text, say that since the coefficient is positive, it indicates a positive relationship between total rainfall and malaria cases. Also, mention that it is a marginal improvement in DIC.

- Tables 3.5 & 3.6: Most of the confidence intervals are given as (0.0, 0.0). This is statistically implausible for confidence intervals of estimates of relative risks. The value of relative risk should not be zero.

5. Discussion & Conclusion

- Start the discussion with a summary statement of key findings, beginning with restating the key finding of identifying specific high-risk districts despite a overall declining trend.

- Major Limitation: A separate paragraph needs to be dedicated to highlighting the major limitation of working with data concerning disaster-associated malarial outbreaks and other unofficial cases.

- Explain the low spatial autocorrelation value of 0.211 and its significance in targeting for intervention.

- The result of rainfall's effect must be qualified in view of the extremely small difference in DIC. Present it as a result that warrants further analysis.

- The recommendation for data access needs to be framed as a necessary step for future research, which arises from the limitation of this study. 6. References and Data - -- Availability ake sure all citations are in the format used in PLOS ONE. Some of them are not complete, for example, [23], [28]. Data Availability Statement (Critical): The present data availability statement on page 31 does not satisfy PLOS ONE's unconditional data availability. You are required to: 1. The analyzed data set with 64 districts used for modeling needs to be deposited in a public repository such as Figshare/Zenodo. 2. Store all of your analysis code in R for WinBUGS in a public repository such as GitHub along with your data deposit. 3. The citation should then be updated with the respective URLs or DOIs for these deposited works. "Will be provided if anyone requires" kinds of sentences are not allowed.

Best,

Reviewer

Reviewer #2: add united nations SDG on the abastract as well as explain and relate to it in the introduction how does your work feed to these goals

the manuscript is well structyured with robust spatial statistical analyisis but the author could improve by adding bivariate LISA analysis and imprving the maps ,

i siuggest the authro run getis ordi GI statistical analysis since the moran i value indicate spatial autocorrelatiom

i also suggest adding the study area map.

Table 3.3: Summary Statistics of Poisson-Gamma, Poisson-lognormal, CAR, Convolution,

CAR_ZIP, Convolution ZIP models :: can be improved by highlighting either through undrlineing or boding variablesmof significance importance

i also suggest adding labelsof all the maps like the one in figure 6

add more literarture in the discussion to support the relevance of the study

Reviewer #3: Please see the review comments. The research needs to be streamlining with theories and must contribute to the existing knowledge. Currently the authors lack a standard review of literature and theoretical understandings. Incorporating theories could make this a better one.

Reviewer #4: Review report

The paper "Spatial Analysis of Malaria in Bangladesh - insights from Bayesian Disease Mapping models" uses advanced spatial statistical approaches to address an essential public health issue that is policy-relevant. The combination of Bayesian disease mapping, spatial autocorrelation diagnostics, zero-inflated Poisson models, and convolution priors is a methodologically solid and ambitious undertaking. The work provides important national insights into malaria risk variability in Bangladesh, with clear implications for malaria elimination strategies aligned with SDG 3.3. Overall, the study is data-driven, analytically rigorous, and shows a thorough understanding of spatial epidemiology modeling. Minor to moderate modifications are recommended to improve scientific robustness, reproducibility, and presentation quality.

1. The abstract effectively describes the background, methods, results, and conclusions. To increase its impact, consider mentioning one or two quantitative performance metrics, such as the ZIP convolution model's DIC improvement or the actual Mann-Kendall τ value. This would quickly demonstrate the study's analytical strength. Minor language tightening is also recommended to eliminate redundancy.

2. Keywords are appropriate and relevant. Consider specifically including "Bangladesh", "Bayesian disease mapping", or "Zero-inflated Poisson model" to increase indexing and discoverability.

3. The introduction presents a comprehensive overview of malaria epidemiology and its geographical implications. The introductory anecdotal examples, such as mosquito metaphors and news allusions, may be condensed to maintain an academic tone. Condensing this part would serve to highlight the research gap, namely the lack of recent nationwide spatial modeling of malaria in Bangladesh.

4. The use of BBS disaster-related malaria data is justified due to access limits. Nonetheless, this constraint should be stated more explicitly as a data proxy, with a brief discussion of how disaster-linked malaria numbers differ from routine monitoring data in volume or spatial bias.

5. While the geometric population growth assumption is plausible, a brief justification or citation demonstrating its relevance to Bangladesh would improve methodological openness.

6. The Mann-Kendall trend test is applied correctly, and the findings are reported clearly. Adding Sen's slope estimate to τ would provide another quantitative measure of the pace of decline.

7. The explanations for Moran's I, Geary's C, and Local Moran's I are detailed and mathematically sound. To increase reproducibility, please define the type of spatial weights matrix utilized (e.g., contiguity-based or distance-based). Whether the weights were row-standardized.

8. The transition from Poisson-Gamma to CAR and convolution models is clearly explained. However, please mention the prior distributions utilized for hyperparameters. The number of MCMC iterations, burn-in time, and convergence diagnostics (such as trace plots or Gelman-Rubin statistics).

9. Using DIC for model comparison is reasonable. Readers who are unfamiliar with Bayesian model selection would benefit from a quick explanation of the magnitude of DIC differences (for example, whether differences greater than 5-10 signal significant progress).

10. Given data constraints, using rainfall as a proxy for cyclone exposure seems sense. However, this assumption should be worded with caution, as rainfall alone may not adequately describe disaster intensity or timeliness.

11. Maps efficiently convey spatial patterns. However, some figures may benefit from greater quality (≥300 dpi). Legends should employ consistent class breaks and decimal precision. North arrows and scale bars should be added when appropriate.

12. The use of confidence intervals to categorize districts as high or low risk is a valid methodological approach. For clarity, state the threshold criterion (e.g., LCL > 1 for high risk) in both the Methods and Results sections.

13. The tables are helpful, but need uniform decimal formatting and better alignment of parameter symbols. Consider shifting large tables to supplementary material to improve readability.

14. The discussion accurately interprets the major findings and relates them to malaria elimination aims. This section might be improved by: more specific comparisons with past geographic malaria studies in Bangladesh and nearby regions, and a deeper understanding of why some northern districts emerge as secondary risk zones.

A separate restrictions paragraph would improve transparency, especially for proxy data use, ecological inference, and spatial resolution constraints.

16. The conclusion is straightforward and policy-relevant. It can be reinforced further by briefly describing future research directions, such as including climatic extremes, mobility data, or spatiotemporal Bayesian models.

Following these minor to moderate adjustments, the manuscript's quality will improve dramatically, attracting more international readers.

Reviewer #5: Authors are kindly reminded to pay attention to how a rigorous scientific research structure should be carried out and to position the research process within a more conducive publication framework. Integrate the research questions to make the research objectives more focused. Please notice the universality and rigor of the data samples. Overall, substantial revision is required before the study can be adequately evaluated.

Reviewer #6: The paper looks at malaria patterns in Bangladesh using Bayesian spatial models. Several model forms are applied including Poisson-based models, CAR, convolution, and a ZIP extension. The aim is to assess spatial clustering, identify high-risk districts, and map relative risk.

The topic is relevant, but there are major problems with the data choice, model implementation, and interpretation of results.

1. The Introduction is not written like a standard scientific paper. It contains stories, metaphors, and cites sources like Wikipedia and Scribbr. The research problem is not clearly stated. The literature review is also weak and needs more discussion of existing malaria and spatial modelling studies.

2. The outcome variable is malaria cases due to disaster from BBS data (see Section 2.1 and the last sentence of page 16 in the analysis). This is not routine malaria surveillance data. Because of this, it is hard to draw proper epidemiological conclusions about malaria risk or its elimination.

This raises some key questions:

* Why was disaster-related BBS data used instead of malaria surveillance data from

DGHS or NMEP?

* What does “malaria due to disaster” actually mean?

* Are these confirmed cases or self reported?

* What was the reasoning behind treating this measure as a proxy for malaria incidence at the district level?

3. The manuscript gives very little information about prior distributions. This is a major omission in a Bayesian analysis.

4. The trace plots shown in the Appendix (Figures 2, 5, 8, 11, 14, 17) do not clearly show convergence for virtually all parameters.

5. Important MCMC details are missing, including number of iterations, burn-in, thinning, number of chains, and the software used.

6. No numerical convergence diagnostics (like R-hat statistics and Effective sample size) are reported. Autocorrelation plots suggest very high autocorrelation for quite a number of the parameters.

7. The reported DIC values raise concerns. Some values are extremely large (for instance,CAR DIC = 26,543 compared with the Convolution ZIP DIC = 98.27), suggesting possible model fitting issues. Some variance estimates are unrealistically large with very wide credible intervals, which again points to convergence problems. A comparison of the other components in Table 3.3 raises similar convergence issues.

8. Kernel density plots in the Appendix (Figures 3, 6, 9, 12, 15, 18) show irregular and sometimes multi-modal distributions, indicating poor convergence or identifiability issues.

9. Several figures, especially spatial maps are difficult to read due to poor resolution and unclear colour scales. Some relative risk values reported seem unrealistic (for instance the number 0.000009 in Table 3.5).

10. Some covariates are poorly defined. For example, it is not clear how rainfall was averaged across time and space.

11. Some equations are repeated and should be properly organised and numbered.

12. The two-part ZIP model is not clearly defined in the methodology. There is also no evidence presented regarding the extent of zero inflation or assessment of overdispersion.

13. The paper discusses malaria elimination, but the outcome variable does not directly measure malaria transmission. The interpretation therefore overstates what the data can support.

14. Many online references are missing access dates (see [2], [4], [5], [15], [17], [18], [19], [20], and [22]).

In summary, while the topic and general modelling framework are appropriate, the paper has serious issues related to data validity, model convergence, and interpretation. These issues need to be addressed before the results can be considered reliable.

**********

what does this mean?). If published, this will include your full peer review and any attached files.

If you choose “no”, your identity will remain anonymous but your review may still be made public.

**Do you want your identity to be public for this peer review?**  For information about this choice, including consent withdrawal, please see our Privacy Policy

Reviewer #1: No

Reviewer #2: **Yes:**  Handsome Bongani Nyoni

Reviewer #3: No

Reviewer #4: No

Reviewer #5: No

Reviewer #6: No

**********

[NOTE: If reviewer comments were submitted as an attachment file, they will be attached to this email and accessible via the submission site. Please log into your account, locate the manuscript record, and check for the action link "View Attachments". If this link does not appear, there are no attachment files.]

To ensure your figures meet our technical requirements, please review our figure guidelines: https://journals.plos.org/plosone/s/figures

You may also use PLOS’s free figure tool, NAAS, to help you prepare publication quality figures: https://journals.plos.org/plosone/s/figures#loc-tools-for-figure-preparation.

NAAS will assess whether your figures meet our technical requirements by comparing each figure against our figure specifications.

Attachment Submitted filename: PONE-D-25-54031_review.pdf

Attachment Submitted filename: Reviewer Comments.pdf

# Author comment: Author response to Decision Letter 1

**DOI:** 10.1371/journal.pone.0353483.r002

20 Mar 2026

Response to Reviewers

Manuscript Title: Spatial Analysis of Malaria in Bangladesh: Insights from Bayesian Disease Mapping Models

Manuscript ID: PONE-D-25-54031

EMID:44506a7af30a78a9

Journal: PLOS ONE

We thank the Academic Editor and reviewers for their careful consideration of our study and constructive comments. In this revised submission, we have undertaken a major revision of the manuscript to address all concerns related to: quality of figures, interpretative clarity, methodological strength and data transparency. We hope that these changes have greatly improved the overall appearance, as well as scientific quality of our study. Every change is traced in the revised document. Here are point-by-point responses to all of the comments.

Response to Additional Editor Comments

PLOS ONE style and file naming requirements

The manuscript has been fully revised to comply with PLOS ONE’s formatting and style guidelines.

Data Availability Statement

The Data Availability Statement has been updated to include the repository name(s) and direct access link(s) to all datasets used in the study

Authors and affiliations

All authors and their respective affiliations are now listed in full, following PLOS ONE author instructions. The corresponding author has been clearly indicated in the manuscript.

Copyright and licensing of map figures (Figures 2–8)

We appreciate the clarification regarding copyright and licensing requirements. We confirm that all map figures (Figures 2–8) were entirely generated by the authors using R software, based exclusively on open-source, publicly available datasets (e.g., administrative boundaries and spatial data from publicly licensed sources).

No proprietary map services, satellite imagery, or software (such as Google Maps, Google Earth, or other copyrighted platforms) were used at any stage.

As such, all figures are original works created by the authors and are fully compatible with publication under the Creative Commons Attribution License (CC BY 4.0).

The figure captions have been updated to explicitly state that the maps were generated by the authors using open-source data and R.

Figure citations in the text

The manuscript text has been revised to ensure that Figures 1, 5, and 6 are explicitly cited and discussed in the relevant sections, enabling proper linking during production.

Table citations in the text

References to Table 3.1 and Table 3.2 have been added to the manuscript text where the results are discussed, ensuring consistency and clarity for readers.

Response to Reviewer #1

Reviewer #1: General Comment

Comment: The manuscript describes a Bayesian spatial model for data related to malarial infections in Bangladesh. The manuscript has a lot of relevance in the domain of public healthcare, but unfortunately, it needs a lot of improvement and modifications in terms of data, methodology, interpretation of results, and manuscript composition.

Response: We appreciate the reviewer for acknowledging the significance of our study. In response to this comment, we have substantially revised the manuscript to improve data transparency, methodological clarity, interpretation of results, and overall presentation.

Reviewer #1: Specific comment-1: Title and Abstract

Comment 1: Include a colon in the title after “Bangladesh”. "Spatial Analysis of Malaria in Bangladesh: Insights from Bayesian Disease Mapping Models".

Response: We agree with the reviewer. As suggested, the title has been changed to "Spatial Analysis of Malaria in Bangladesh: Insights from Bayesian Disease Mapping Models."

Comment 2: There appears to be a discrepancy between a citation for a report from "Bangladesh Bureau of Statistics" in the abstract, and other, mostly news article citations in methods. This major discrepancy needs to be taken care of.

Response: We appreciate you for bringing attention to this crucial matter. The Abstract and Methods sections have been updated to provide a clear and consistent description of the study's data sources. In particular, we now make it clear that:

The Bangladesh Bureau of Statistics' (BBS) Bangladesh Disaster-related Statistics 2021 report provided district-level malaria data;

The World Malaria Reports (2024, 2025) published by the World Health Organization (WHO) and Bangladesh's National Strategic Plan for Malaria Elimination (2021–2025) published by the Asia Pacific Malaria Elimination Network (APMEN) provided the national malaria time-series data.;

The Bangladesh Water Development Board provided the rainfall data.

These changes eliminate any doubt about the source of the data and guarantee coherence between the Abstract and Methods sections.

Comment 3: The abstract refers to using rain as a covariate, but it does not report that it was a significant predictor, for example. Summarize this result.

Response: We appreciate this comment. In the revised Abstract, we now explicitly summarize the role of rainfall based on model comparison. Because rainfall was evaluated using DIC-based model selection rather than parameter-level significance testing, we have carefully avoided over-interpretation. The Results section of the Abstract now states:

“Incorporating district-level total rainfall as a covariate resulted in a marginal improvement in model fit (ΔDIC ≈ 1), suggesting a modest contribution of rainfall.”

Reviewer #1: Specific comment-2: Introduction

Comment-1: Instead of starting with a story ("There is a saying that 'to break a butterfly upon a wheel'."), introduce a direct explanation of how much malaria affects the public health of Bangladesh and the goal of elimination.

Response: We agree with the reviewer and have removed the story-based opening. The Introduction now starts by clearly describing the public health burden of malaria in Bangladesh and highlights the country’s elimination goals, including commitments under SDG 3.3 and the National Malaria Elimination Programme (NMEP) 2024–2030 plan.

Comment-2: The literature review would benefit from being more specific concerning the knowledge gap: there has been a lack of recent, country-wide spatial analyses using data which covers all malaria parasite species, not just P. falciparum.

Response: We thank the reviewer for this comment. The literature review has been substantially refined to clearly articulate the existing knowledge gap. In the revised manuscript, we now explicitly state that prior studies in Bangladesh have predominantly focused on selected high-endemic districts, border regions, or exclusively on Plasmodium falciparum infections. We emphasize that recent nationwide spatial analyses incorporating all malaria parasite species and using updated data are limited. This clarification strengthens the justification for the present study and highlights its contribution to the existing literature.

Comment-3: The last paragraph needs to identify specifically the objectives of the study.

Response: We have revised the last paragraph of the Introduction so and the study objectives are now presented in bullet points and clearly defined. This format ensures that the study goals are explicit and directly connected to our methods and policy relevance.

Reviewer #1: Specific comment-3: Methods

Reviewer #1: Specific comment-3: Methods-Data sources

Comment-1: The mention of sources for cases of malaria is not acceptable in a research article. Relying on “the leading newspaper, the Daily Sun” and a published paper for a secondary analysis are not trustworthy. The sources you use should be official data from either the National Malaria Elimination Program (NMEP) or the Directorate General of Health Services (DGHS). If this becomes genuinely impossible, this would become a major limitation.

Response: We agree with this concern. All references to newspaper reports and secondary summaries have been removed. The revised manuscript relies only on official government and international sources. However, district-level routine malaria surveillance data from DGHS/NMEP are not publicly accessible due to access restrictions. This unavailability is now explicitly stated and treated as a major limitation.

Comment-2: Explain, with justification, why “malaria (due to disaster)” data in the BBS 2021 report is used as a proxy for total malaria spatial burden. This represents a major limitation.

Response: Because routine district-level surveillance data are unavailable, we use the “population suffering from malaria due to disaster” variable from BBS 2021 as a proxy for relative spatial malaria burden. This proxy is justified because climate-related disasters such as floods, cyclones, waterlogging, are known to intensify malaria transmission by creating mosquito breeding sites, disrupting healthcare access, and increasing population displacement. Areas reporting malaria cases due to disaster are therefore likely to have existing malaria transmission that becomes amplified under environmental stress. Consequently, repeated reporting of malaria in disaster contexts reflects underlying spatial vulnerability rather than isolated events.

Comment-3: To project population, you would identify the years when data for initial population size (`P_0`) and final population size (`P_n`) are needed for calculation of geometric rate of growth.

Response: We have clarified that population growth rates are calculated using census data from 2011 (P0) and 2022 (Pₙ), and that this rate is then used to project district populations for 2020. The relevant years and equations are now explicitly stated in the Methods section.

Reviewer #1: Specific comment-3: Methods-Statistical methods

Comment-1: In Section 2.11, justify how the adjacency matrix (`w_ij`) was defined for CAR and CONVOLUTION models in terms of queen contiguity or distance-based definition.

Response: The adjacency matrix was defined using queen contiguity and now it is stated on both these models method description.

Comment-2: While introducing models like Poisson Gamma, CAR models, etc., it is important to mention briefly for what purpose a particular model is used.

Response: The section Bayesian spatial model is revised with Bayesian hierarchical spatial models which contains purpose of each model by clarifying how each address overdispersion, spatial dependence, or excess zeros.

Comment-3: Ethics Statement: While the statement on page 31 seems fine, it would be appropriate to change “N/A” in the submission form entry on page 3 to reflect that it involves publicly available, aggregated statistical data.

Response: Thank you for this helpful observation.

Reviewer #1: Specific comment-4: Results

Comment-1: Figure 1-The description of this graph needs to mention what data points are being shown.

Response: We have revised the caption of Figure to clearly specify the data points shown are the figure presents annual confirmed malaria cases in Bangladesh as reported by NMEP and WHO.

Comment-2: Figures 2, 3, 4, 6, 7, 8: Make sure that all maps contain a scale bar, a north arrow, and legible legends. The colors used in the LISA map, in Figure 4, should be defined in the caption.

I think the figures can be improved and present in best way.

Response: We revised all maps to include scale bars, north arrows, legible legends, and defined LISA colors in its caption.

Comment-3: Spatial Autocorrelation: While discussing Moran's I of 0.211, avoid using "small." Instead, say it "indicates positive spatial autocorrelation, which is statistically significant with a p-value of <0.001."

Response: We have revised the manuscript by removing the term “small” and now state that Moran’s I indicate positive spatial autocorrelation that is statistically significant (p < 0.001), in line with the reviewer’s recommendation.

Comment-4: Table 3.3: There are no descriptive titles for the column headers. Use descriptive names. Symbol definitions are needed in the caption for this and all other tables. Examples are σ_u^2, τ_u^2, p_D.

Response: Table 3.3 has been revised and all statistical symbols (σᵤ², τᵤ², p_D, DIC) are now explicitly defined in the table caption.

Comment-5: Rainfall Covariate: There is no interpretation of results for the rainfall covariate. To report in the text, say that since the coefficient is positive, it indicates a positive relationship between total rainfall and malaria cases. Also, mention that it is a marginal improvement in DIC.

Response: The Results section has been revised to explicitly interpret the rainfall covariate.

Previously we used iteration 18000 with a burn in 5000 and thin 5, now we are using iteration 660000 with a burn-in 300000 and thin 5 which reduces the previous marginal improvent.

Comment-6: Tables 3.5 & 3.6: Most of the confidence intervals are given as (0.0, 0.0). This is statistically implausible for confidence intervals of estimates of relative risks. The value of relative risk should not be zero.

Response: Relative risk (RR) in our study is defined as the ratio of the model-based posterior mean of the fitted count (μ �_i) to the expected count (E_i), i.e., RR_i=μ �_i/E_i.

For areas where the observed count is zero, the model estimates a very small positive fitted value (for example, 0.00015) rather than exactly zero. This occurs because, under a Poisson-based framework, the mean parameter is strictly positive. When such small fitted values are divided by the expected count, the resulting relative risks are correspondingly very small. We now fully concentrated on the high risk areas only.

Reviewer #1: Specific comment-5: Discussion & Conclusion

Comment-1: Start the discussion with a summary statement of key findings, beginning with restating the key finding of identifying specific high-risk districts despite a overall declining trend.

Response: We have revised the discussion opening to emphasize that, despite an overall national decline in malaria, specific districts—primarily in the Chittagong Hill Tracts and selected northern areas—remain high-risk.

Comment-2: Major Limitation: A separate paragraph needs to be dedicated to highlighting the major limitation of working with data concerning disaster-associated malarial outbreaks and other unofficial cases.

Response: A new paragraph has been added to clearly discuss the limitation of using disaster-associated malaria data as a proxy, noting potential underrepresentation of asymptomatic and non-disaster-related cases.

Comment-3: Explain the low spatial autocorrelation value of 0.211 and its significance in targeting for intervention.

Response: We clarified that the Global Moran’s I value of 0.211 indicates moderate but statistically significant spatial autocorrelation, suggesting geographically targeted interventions may be more effective than uniform national strategies.

Comment: The result of rainfall's effect must be qualified in view of the extremely small difference in DIC. Present it as a result that warrants further analysis.

Response: We have revised the text to note that although rainfall showed a positive association with malaria vulnerability, there is a marginal increase in DIC.

Comment: The recommendation for data access needs to be framed as a necessary step for future research, which arises from the limitation of this study.

Response: In response, we now state that “improved public access to routine surveillance data is essential for future research.”

Reviewer #1: Specific comment-5: References and Data Availability

Comment: Make sure all citations are in the format used in PLOS ONE. Some of them are not complete, for example, [23], [28]. Data Availability Statement (Critical): The present data availability statement on page 31 does not satisfy PLOS ONE's unconditional data availability. You are required to: 1. The analyzed data set with 64 districts used for modeling needs to be deposited in a public repository such as Figshare/Zenodo. 2. Store all of your analysis code in R for WinBUGS in a public repository such as GitHub along with your data deposit. 3. The citation should then be updated with the respective URLs or DOIs for these deposited works. "Will be provided if anyone requires" kinds of sentences are not allowed.

Response: We provided the appropriate link for this.

Response to Reviewer #2

Reviewer #2: Specific comment-1

Comment: Add united nations SDG on the abstract as

# Peer review: Decision Letter 1

**DOI:** 10.1371/journal.pone.0353483.r003

**Authors:** Denekew Bitew Belay

24 Apr 2026

Dear Dr. Karim,

Thank you for submitting your manuscript to PLOS ONE. After careful consideration, we feel that it has merit but does not fully meet PLOS ONE’s publication criteria as it currently stands. Therefore, we invite you to submit a revised version of the manuscript that addresses the points raised during the review process.

Please submit your revised manuscript by Jun 08 2026 11:59PM. If you will need more time than this to complete your revisions, please reply to this message or contact the journal office at plosone@plos.org. When you're ready to submit your revision, log on to https://www.editorialmanager.com/pone/ and select the 'Submissions Needing Revision' folder to locate your manuscript file.

- A letter that responds to each point raised by the academic editor and reviewer(s). You should upload this letter as a separate file labeled 'Response to Reviewers'.
- A marked-up copy of your manuscript that highlights changes made to the original version. You should upload this as a separate file labeled 'Revised Manuscript with Track Changes'.
- An unmarked version of your revised paper without tracked changes. You should upload this as a separate file labeled 'Manuscript'.

If you would like to make changes to your financial disclosure, please include your updated statement in your cover letter. Guidelines for resubmitting your figure files are available below the reviewer comments at the end of this letter.

If applicable, we recommend that you deposit your laboratory protocols in protocols.io to enhance the reproducibility of your results. Protocols.io assigns your protocol its own identifier (DOI) so that it can be cited independently in the future. For instructions see: https://journals.plos.org/plosone/s/submission-guidelines#loc-laboratory-protocols. Additionally, PLOS ONE offers an option for publishing peer-reviewed Lab Protocol articles, which describe protocols hosted on protocols.io. Read more information on sharing protocols at https://plos.org/protocols?utm_medium=editorial-email&utm_source=authorletters&utm_campaign=protocols.

As the corresponding author, your ORCID iD is verified in the submission system and will appear in the published article. PLOS supports the use of ORCID, and we encourage all coauthors to register for an ORCID iD and use it as well. Please encourage your coauthors to verify their ORCID iD within the submission system before final acceptance, as unverified ORCID iDs will not appear in the published article. *Only*  the individual author can complete the verification step; PLOS staff *cannot*  verify ORCID iDs on behalf of authors.

We look forward to receiving your revised manuscript.

Kind regards,

Denekew Bitew Belay, Ph.D

Academic Editor

PLOS One

Journal Requirements:

If the reviewer comments include a recommendation to cite specific previously published works, please review and evaluate these publications to determine whether they are relevant and should be cited. There is no requirement to cite these works unless the editor has indicated otherwise.

[Note: HTML markup is below. Please do not edit.]

Reviewers' comments:

Reviewer's Responses to Questions

**Comments to the Author**

Reviewer #1: All comments have been addressed

Reviewer #2: All comments have been addressed

Reviewer #3: (No Response)

Reviewer #6: (No Response)

**********

2. Is the manuscript technically sound, and do the data support the conclusions?

Reviewer #1: Yes

Reviewer #2: Yes

Reviewer #3: Yes

Reviewer #6: Partly

**********

3. Has the statistical analysis been performed appropriately and rigorously?

Reviewer #1: Yes

Reviewer #2: Yes

Reviewer #3: Yes

Reviewer #6: No

**********

4. Have the authors made all data underlying the findings in their manuscript fully available?

The PLOS Data policy

Reviewer #1: Yes

Reviewer #2: Yes

Reviewer #3: Yes

Reviewer #6: Yes

**********

5. Is the manuscript presented in an intelligible fashion and written in standard English?

Reviewer #1: Yes

Reviewer #2: Yes

Reviewer #3: Yes

Reviewer #6: Yes

**********

Reviewer #1: Thank you for revising the paper. I appreciate the authors' efforts to address my previous comments.

Reviewer #2: Good Day Authors , thank you so much for addresing all the highlighted cpmments , the manuscript is now technical and scientific impact sound

Reviewer #3: Dear authors, I'm pleased to see the comprehensive inputs to address the comments. The research seems to be justified at current form. I do appreciate your thoughts. I do not have any queries further.

Reviewer #6: (No Response)

**********

what does this mean?). If published, this will include your full peer review and any attached files.

If you choose “no”, your identity will remain anonymous but your review may still be made public.

**Do you want your identity to be public for this peer review?**  For information about this choice, including consent withdrawal, please see our Privacy Policy

Reviewer #1: **Yes:**  Dr.Alireza Mohammadi, Department of Geographay and Urban Planning, Ardabil, Iran

Reviewer #2: **Yes:**  Handsome bongani nyoni

Reviewer #3: No

Reviewer #6: No

**********

[NOTE: If reviewer comments were submitted as an attachment file, they will be attached to this email and accessible via the submission site. Please log into your account, locate the manuscript record, and check for the action link "View Attachments". If this link does not appear, there are no attachment files.]

To ensure your figures meet our technical requirements, please review our figure guidelines: https://journals.plos.org/plosone/s/figures

You may also use PLOS’s free figure tool, NAAS, to help you prepare publication quality figures: https://journals.plos.org/plosone/s/figures#loc-tools-for-figure-preparation.

NAAS will assess whether your figures meet our technical requirements by comparing each figure against our figure specifications.

Attachment Submitted filename: PONE-D-25-54031R1 Reviewer Report.pdf

# Author comment: Author response to Decision Letter 2

**DOI:** 10.1371/journal.pone.0353483.r004

17 May 2026

Manuscript Title: Spatial Analysis of Malaria in Bangladesh: Insights from Bayesian Disease Mapping Models

Manuscript ID: PONE-D-25-54031 (Revision 1)

Journal: PLOS ONE

We sincerely thank the reviewer for the careful evaluation of our revised manuscript and for the constructive comments provided. We appreciate the reviewer’s insightful suggestions, which have helped us further improve the clarity, rigor, and transparency of our work. Below, we address each point in detail.

Comment #1: Introduction Style and Literature Review

Comment: The Introduction has been improved and now reads more professionally. However, the literature review still relies heavily on the same set of regional studies cited in the original version. The authors have not added meaningful engagement with broader spatial epidemiology literature beyond Bangladesh.

Response: We have revised the Introduction to incorporate broader spatial epidemiology literature beyond Bangladesh. Specifically, we have added references to studies from India, Nepal, and sub-Saharan Africa that apply spatial statistical methods to malaria and demonstrate spatial clustering, ecological heterogeneity, and hotspot persistence.

Comment #2: Outcome Variable Justification

Comment 2(i): The authors do not specify whether “malaria due to disaster” cases are laboratory confirmed, clinically diagnosed, or self-reported.

Response: The “malaria due to disaster” data was collected from Bangladesh Disaster-related Statistics 2021(BDRS-2021) report published by the Bangladesh Bureau of Statistics (BBS). This is administratively reported cases for the period 2015–2020 and does not distinguish specifically between laboratory-confirmed, clinically diagnosed, or self-reported cases.

Comment 2(ii): The Methods section (Page 4) states the data cover “between 2015 and 2020” but the analysis section (Page 16 onward) uses a single aggregated measure and this aggregation over time is not captured in the Methods section.

Furthermore, the temporal alignment between the disaster-attributed cases (2015-2020) and the rainfall covariate (2017-2020) is unclear.

Response: The malaria dataset from the BDRS-2021 report represents a single district-level cumulative statistic aggregated over the period 2015–2020. This clarification has now been explicitly added to the Methods (Data source) like this “Importantly, the reported values represent a single cumulative (aggregated) statistic for the entire period 2015–2020. Therefore, each district is represented by one value reflecting the total burden over the period.” to improve transparency regarding temporal aggregation.

For rainfall, we use district-level annual rainfall data for 2017–2020, as data for 2015–2016 are not available, and construct a cumulative rainfall measure over this period. This is done to ensure consistency with the aggregated structure of the malaria outcome variable. Since the malaria data is a fully aggregated outcome over 2015–2020 and the analysis is conducted at the district level as a cross-sectional framework, the rainfall measure is used as an aggregated environmental exposure over the available and most consistent period, and this temporal limitation does not affect the validity of the model structure.

Comment #6: Numerical Convergence Diagnostics

Comment: For the preferred Conv. ZIP model, the effective sample sizes are adequate (e.g., 140,000 for the mean). However, the Poisson-Lognormal and Conv. models have very low effective sample sizes (as low as 89 and 183), yet they remain in Table 3. The inclusion of models with clear convergence problems in Table 3 is problematic.

Response: The CAR, Convolution, and CAR ZIP models are retained in Table 3 to provide a comprehensive comparison of alternative spatial and zero-inflated specifications, allowing assessment of model performance under different assumptions. To avoid any ambiguity, we have added a table footnote recommended by the reviewer.

Comment #7: Model Comparison: DIC and Parameter Estimates

Comment: The presence of multiple poorly fitting models in Table 3 undermines confidence in the model comparison framework. Extremely large DIC values and implausible variance estimates persist. Either add the recommended footnote to Table 3.

Response: We have added the recommended footnote to Table 3.

Comment #8: Kernel Density Plots

Comment: The authors removed the kernel density plots entirely from the revised Appendix, which means readers cannot evaluate posterior distributions for themselves. Consider adding kernel density plots for the final model to the Appendix.

Response: Kernel density plots of the final model have been added to the Appendix to allow readers to assess posterior distributions.

Comment #9: Figure Quality and Relative Risk Values

Comment: The maps look better now, although it is still a bit difficult to fully assess the resolution from the files provided. To get this issue resolved, authors may write R codes to save the images directly.

Response: Thank you for the suggestion. All figures in the revised manuscript have been generated and exported directly from R in TIFF format at 300 dpi resolution, consistent with journal guidelines. We believe the current figure quality is appropriate for publication.

Comment #12: ZIP Model Definition and Zero Inflation Evidence

Comment: The ZIP model structure is now defined, and evidence of zero inflation and overdispersion is presented. However, the results for the zero-inflation component (e.g., the estimated probability π of an excess zero) are still not reported in Tables 3 or 4. The authors mention a Uniform (0,1) prior for zero-inflation probability in Section 2.18, but the posterior estimate is never presented or discussed.

Response: The posterior estimate of the zero-inflation probability (π) has now been reported and discussed in the Results section, demonstrating the presence of excess zeros in the data.

Comment #13: Overinterpretation of Malaria Elimination

Comment: The Discussion and Conclusion have been substantially rewritten with more cautious language. However, the Abstract still states “Bangladesh is making significant progress toward malaria elimination” and “many districts already achieving zero cases” based on disaster-attributed data, which remains an overstatement. Revise the Abstract to reflect disaster-attributed data limitations.

Response: The Abstract has been revised to avoid overstatement and to more accurately reflect the nature of the data used in this study. Specifically, we clarified that the district-level malaria variable represents disaster-associated aggregated counts and is used as a proxy indicator of relative malaria vulnerability rather than a direct measure of malaria transmission. Also, the revised Abstract now explicitly states that the findings should be interpreted as relative spatial risk patterns under data limitations.

Comment #15: Additional issues raised regarding Table 3 and 4

Comment 15(i): The way the R � and neff values are reported in parentheses in Tables 3 and 4 is confusing.

Consider reporting the Gelman-Rubin values to two decimal places (e.g., 1.02) leaving a space after the comma for clarity. For example, write “(1.02, 140000)” instead of “(1,140000)”.

Response: The formatting has been corrected to improve clarity, with appropriate spacing and decimal representation.

Comment 15(ii): The caption of Table 3 defines τu2 as “the variance of unstructured heterogeneity” but the corresponding row label reads “Spatial precision”. This is a contradiction. Additionally, Table 4 includes τu2 in the caption, but no column named τu2 appears in the table. Both captions must be corrected for consistency.

Response: Thank you for this helpful suggestion. We have revised the captions of Tables 3 and 4. The revised captions ensure that all notation is clearly defined and consistently used across tables, addressing the reviewer’s concern.

Comment 15(iii): For the Conv. ZIP model, σv2 = 1.957 and τv2 = 0.661. If τ2 = 1/σ2, then 1/1.957 = 0.511, not 0.661. Similarly, σu2 = 0.229 and τu2 = 89.540, but 1/0.229 = 4.367; therefore, we would expect the value to be approximately 4.367, not 89.540. The authors should clarify whether τ2 is defined as 1/σ2 or estimated as a separate parameter.

Response: Thank you for this important observation. In our model, variance is obtained at each MCMC iteration as σ^2=1/τ, and the reported posterior mean corresponds to the average of these transformed values, i.e., E[1/τ]. The precision parameter is summarized separately as E[τ]. Since the averaging is performed after transformation, the reported variance corresponds to mean(1/τ), not 1/mean(τ). Therefore, the two quantities are not exact reciprocals, which explains the observed difference. Also, we have now clarified in the manuscript that variance parameters are defined as the inverse of precision and are computed at each MCMC iteration.

Comment 15(iv): The footnote of Table 3 states that bold values denote “parameters of significant importance” but no statistical criterion is provided to define what constitutes “significant importance”.

Response: We agree that the previous wording was unclear and could be misinterpreted. We have revised the table footnote to specify that bold formatting is applied to the posterior mean, variance components, 95% credible interval of posterior mean and DIC solely for readability.

Attachment Submitted filename: Response to Reviewers.docx

# Peer review: Decision Letter 2

**DOI:** 10.1371/journal.pone.0353483.r005

**Authors:** Denekew Bitew Belay

25 Jun 2026

Spatial Analysis of Malaria in Bangladesh-insights from Bayesian Disease Mapping models

PONE-D-25-54031R2

Dear Dr. Karim,

We’re pleased to inform you that your manuscript has been judged scientifically suitable for publication and will be formally accepted for publication once it meets all outstanding technical requirements.

Within one week, you’ll receive an e-mail detailing the required amendments. When these have been addressed, you’ll receive a formal acceptance letter and your manuscript will be scheduled for publication.

An invoice will be generated when your article is formally accepted. Please note, if your institution has a publishing partnership with PLOS and your article meets the relevant criteria, all or part of your publication costs will be covered. Please make sure your user information is up-to-date by logging into Editorial Manager at Editorial Manager® and clicking the ‘Update My Information' link at the top of the page. For questions related to billing, please contact billing support.

If your institution or institutions have a press office, please notify them about your upcoming paper to help maximize its impact. If they’ll be preparing press materials, please inform our press team as soon as possible -- no later than 48 hours after receiving the formal acceptance. Your manuscript will remain under strict press embargo until 2 pm Eastern Time on the date of publication. For more information, please contact onepress@plos.org.

Kind regards,

Denekew Bitew Belay, Ph.D

Academic Editor

PLOS One

Additional Editor Comments (optional):

Reviewers' comments:

Reviewer's Responses to Questions

**Comments to the Author**

Reviewer #6: All comments have been addressed

**********

2. Is the manuscript technically sound, and do the data support the conclusions?

Reviewer #6: Yes

**********

3. Has the statistical analysis been performed appropriately and rigorously?

Reviewer #6: Yes

**********

4. Have the authors made all data underlying the findings in their manuscript fully available?

The PLOS Data policy

Reviewer #6: Yes

**********

5. Is the manuscript presented in an intelligible fashion and written in standard English?

Reviewer #6: Yes

**********

Reviewer #6: The authors have thoroughly addressed all concerns raised earlier. The manuscript

in my opinion, is now methodologically sound, transparent about its limitations and clearly

written.

**********

what does this mean?). If published, this will include your full peer review and any attached files.

If you choose “no”, your identity will remain anonymous but your review may still be made public.

**Do you want your identity to be public for this peer review?**  For information about this choice, including consent withdrawal, please see our Privacy Policy

Reviewer #6: No

**********

Attachment Submitted filename: PONE-D-25-54031R2 Reviewer Report.pdf

# Editor report: Acceptance letter

**DOI:** 10.1371/journal.pone.0353483.r006

**Authors:** Denekew Bitew Belay

PONE-D-25-54031R2

PLOS One

Dear Dr. Karim,

I'm pleased to inform you that your manuscript has been deemed suitable for publication in PLOS One. Congratulations! Your manuscript is now being handed over to our production team.

At this stage, our production department will prepare your paper for publication. This includes ensuring the following:

* All references, tables, and figures are properly cited

* All relevant supporting information is included in the manuscript submission,

* There are no issues that prevent the paper from being properly typeset

You will receive further instructions from the production team, including instructions on how to review your proof when it is ready. Please keep in mind that we are working through a large volume of accepted articles, so please give us a few days to review your paper and let you know the next and final steps.

Lastly, if your institution or institutions have a press office, please let them know about your upcoming paper now to help maximize its impact. If they'll be preparing press materials, please inform our press team within the next 48 hours. Your manuscript will remain under strict press embargo until 2 pm Eastern Time on the date of publication. For more information, please contact onepress@plos.org.

You will receive an invoice from PLOS for your publication fee after your manuscript has reached the completed accept phase. If you receive an email requesting payment before acceptance or for any other service, this may be a phishing scheme. Learn how to identify phishing emails and protect your accounts at https://explore.plos.org/phishing.

If we can help with anything else, please email us at customercare@plos.org.

Thank you for submitting your work to PLOS ONE and supporting open access.

Kind regards,

PLOS ONE Editorial Office Staff

on behalf of

Dr. Denekew Bitew Belay

Academic Editor

PLOS One
